# Supplementary material for: Ability of the ash dieback pathogen to reproduce and to induce damage on its host are controlled by different environmental parameters
Source: PLoS Pathog. 2023 Apr 20;19(4):e1010558. doi: 10.1371/journal.ppat.1010558 (PMC10153702; doi:10.1371/journal.ppat.1010558)
Supplement: S1 Table — (DOCX) [file ppat.1010558.s001.docx]

S1 Table. Studied sites

| Plot | Region  Name | Coordinates | Mean summer temperature (°C, , July, August,  2001-21) | Summer rainfall (mm, July, August, 2001-21) | Year of data collection | | % tree cover |
| --- | --- | --- | --- | --- | --- | --- | --- |
|  |  |  |  |  | Leaf infection | Shoot mortality |  |
| Am | Hauts-de-France  Fréchencourt | 2.43261 ; 49.97479 | 18.0 | 136.0 | 2016-20 | 2017-21 | 76 |
| Ch1 Ch2 Ch3 Ch4 Ch5 Ch6 Gr Se | Grand-Est  Champenoux  Champenoux  Champenoux  Champenoux  Champenoux  Champenoux  Gremecey  Seichamps | 6.34521 ; 48.75327  6.31782 ; 48.73877 6.33035 ; 48.75654 6.35984 ; 48.73698 6.37312 ; 48.74222 6.32135; 48.73938 6.43011 ; 48.81474 6.26910 ; 48.72059 | 19.4 19.4 19.4 19.4 19.4 19.4 19.2 19.6 | 139.1 139.1 139.1  139.1  139.1  139.1 127.8 141.7 | 2015-21 2016-21 2019-21 2019-21 2019-21 2019-21 2014-15 2013-14 | 2016-22 2017-22 2020-22 2020-22 2020-22 2020-22 2015 ^a^ | 97 21 95 3 94 9 100 15 |
| Lu Ro | Auvergne-Rhône-Alpes  Lupé  Roche-sur-Grane | 4.71034 ; 45.37561 4.93764 ; 44.68831 | 21.6 21.5 | 133.2 130.3 | 2016-20 2016-20 | 2017-20 ^a^ 2017-20 ^b^ | 75 63 |
| Co Sa Be Ol1 Ol2  Fe | Nouvelle-Aquitaine  Colombier   Salignac-sur-Charente  Sarrance  Oloron-Sainte-Marie  Oloron-Sainte-Marie  Ance Féas | -0.55205 ; 45.66159 -0.39385 ; 45.66485 -0.58197 ; 43.02852 -0.54005 ; 43.11422 -0.53388 ; 43.12531 -0.65832 ; 43.15170 | 20.4 20.7 15.9 19.1 19.1 20.1 | 92.3 96.1 185.6 155.6 155.6 155.7 | 2018-19 2018-19 2020 2020 2020 2020 | 2019 ^a^ 2019 ^a^ 2021 2021 2021 2021 | 62 91 77 100 100 100 |
| La Po Sc | Bretagne  Landivisiau  Pont-de-Buis Les Quimerch  Scaër | -4.08828 ; 48.52195 -4.07908 ; 48.24803  -3.70625 ; 48.03131 | 16.7 17.2 16.6 | 131.2 140.9 158.4 | 2020 2020 2020 | ^b^ ^b^ ^b^ | 42 95 83 |

^a^ Data not available for shoot mortality because the site was destroyed during the winter

^b^ Data not recorded in spring 2021
