# Supplementary material for: Ability of the ash dieback pathogen to reproduce and to induce damage on its host are controlled by different environmental parameters
Source: PLoS Pathog. 2023 Apr 20;19(4):e1010558. doi: 10.1371/journal.ppat.1010558 (PMC10153702; doi:10.1371/journal.ppat.1010558)
Supplement: S1 Fig — A. Leaf necrosis, B. shoot mortality. (DOCX) [file ppat.1010558.s002.docx]

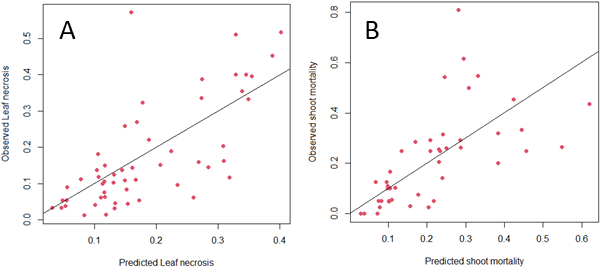


S1 Fig. Cross-validation for the model at the plot level relating climatic and site parameters to leak necrosis and shoot mortality likelihood. A. Leaf necrosis, B. shoot mortality
