# Supplementary material for: Ability of the ash dieback pathogen to reproduce and to induce damage on its host are controlled by different environmental parameters
Source: PLoS Pathog. 2023 Apr 20;19(4):e1010558. doi: 10.1371/journal.ppat.1010558 (PMC10153702; doi:10.1371/journal.ppat.1010558)
Supplement: S2 Fig — Quantile 0.05 (Q5) and 0.95 (Q95) for the estimated probability of leaf necrosis (A, B) and shoot mortality (C, D). The map were computed using the parameter 0.05 and 0.95 quantiles obtained from the Bayesian procedure fit. The shoot mortality is computed for a forest situation (tree cover of 100%). (DOCX) [file ppat.1010558.s003.docx]

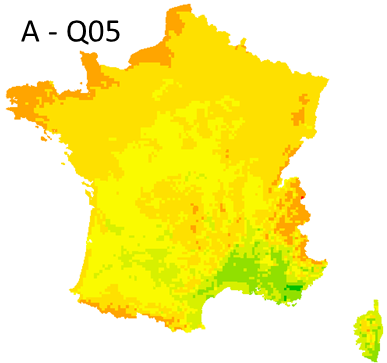

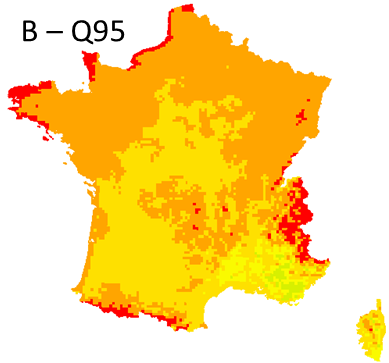


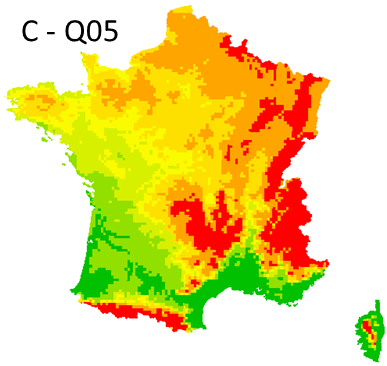

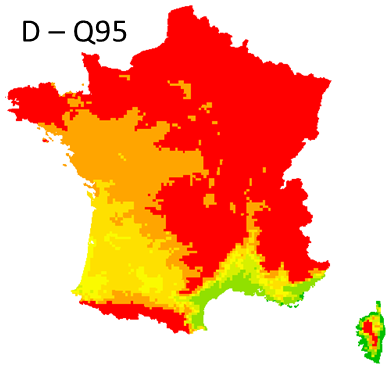


S2 Fig. Quantile 0.05 (Q5) and 0.95 (Q95) for the estimated likelihood of leaf necrosis (A, B) and shoot mortality (C, D). The map were computed using the parameter 0.05 and 0.95 quantiles obtained from the Bayesian procedure fit. The shoot mortality is computed for a forest situation (tree cover of 100%).
