# Supplementary material for: Ability of the ash dieback pathogen to reproduce and to induce damage on its host are controlled by different environmental parameters
Source: PLoS Pathog. 2023 Apr 20;19(4):e1010558. doi: 10.1371/journal.ppat.1010558 (PMC10153702; doi:10.1371/journal.ppat.1010558)
Supplement: Attachment — Submitted filename: PPATHOGENS-D-22-00782_R2.pdf [file ppat.1010558.s008.pdf]

PLOS Pathogens

Ability of the Ash dieback pathogen to reproduce and to induce damage on its host are controlled by different environmental parameters

--Manuscript Draft--

Manuscript Number:	PPATHOGENS-D-22-00782R2
Full Title:	Ability of the Ash dieback pathogen to reproduce and to induce damage on its host are controlled by different environmental parameters
Short Title:	Environmental influence on ash dieback
Article Type:	Research Article
Section/Category:	Plant Pathogens
Keywords:	Hymenoscyphus fraxineus; climate; healthy carrier; latent infection; apothecia production
Corresponding Author:	Benoit Marçais<br>INRAE Centre Grand-Est Nancy: Institut National de Recherche pour l'Agriculture l'Alimentation et l'Environnement Centre Grand-Est Nancy<br>Champenoux, FRANCE
Corresponding Author's Institution:	INRAE Centre Grand-Est Nancy: Institut National de Recherche pour l'Agriculture l'Alimentation et l'Environnement Centre Grand-Est Nancy
First Author:	Benoit Marçais
Order of Authors:	Benoit Marçais
	Arnaud Giraudel
	Claude Husson
Abstract:	Ash dieback, induced by an invasive ascomycete, Hymenoscyphus fraxineus, has emerged in the late 1990s as a severe disease threatening ash populations in Europe. Future prospects for ash are improved by the existence of individuals with natural genetic resistance or tolerance to the disease and by limited disease impact in many environmental conditions where ashes are frequent. Nevertheless, it was suggested that, even in those conditions, ash trees are infected and enable pathogen transmission. We studied the influence of climate and local environment on the ability of H. fraxineus to infect, be transmitted and cause damage on its host. We showed that healthy carrier, i.e. individuals showing no dieback but carrying H. fraxineus, exist and may play a significant role in ash dieback epidemiology. Environment strongly influenced H. fraxineus with different parameters being important depending on the life cycle stage. The ability of H. fraxineus to establish on ash leaves and to reproduce on the leaf debris in the litter (rachises) mainly depended on total precipitations in July-August and was not influenced by local tree cover. By contrast, damages to the host, and in particular shoot mortality was significantly reduced by high summer temperature in July-August and by high autumn average temperature. As a consequence, in many situations, ash trees are infected and enable H. fraxineus transmission while showing limited or even no damages. We also observed a decreasing trend of severity (leaf necrosis and shoot mortality likelihood) with the time of disease presence in a plot that could be significant for the future of ash dieback.
Suggested Reviewers:	Thomas Kirisits<br>BOKU, University of Natural ressources and Life Science, Vienna, Austria<br>thomas.kirisits@boku.ac.at<br>specialist of Ash diaback, large experience in tree diseases
	Joan Webber<br>Alice Holt Research Station: Forest Research<br>joan.webber@forestry.gsi.gov.uk<br>Experience with many tree disease
	Treena Burguess<br>Murdoch University Faculty of Health Sciences: Murdoch University

Powered by Editorial Manager® and ProduXion Manager® from Aries Systems Corporation

	tburgess@murdoch.edu<br>extensive experience with tree disease
	Garbelotto Matteo<br>UC Berkeley: University of California Berkeley<br>matteog@berkeley.edu<br>extensive experience with tree disease
Opposed Reviewers:	
Additional Information:	
Question	Response
**Financial Disclosure**<br><br>Enter a financial disclosure statement that describes the sources of funding for the work included in this submission. Review the submission guidelines for detailed requirements. View published research articles from *PLOS Pathogens* for specific examples.<br><br>This statement is required for submission and **will appear in the published article** if the submission is accepted. Please make sure it is accurate.<br><br>**Unfunded studies**<br>Enter: *The author(s) received no specific funding for this work.*<br><br>**Funded studies**<br><br>Enter a statement with the following details:<br>• Initials of the authors who received each award<br>• Grant numbers awarded to each author<br>• The full name of each funder<br>• URL of each funder website<br>• Did the sponsors or funders play any role in the study design, data collection and analysis, decision to publish, or preparation of the manuscript?<br>• **NO** - Include this sentence at the end of your statement: *The funders had no role in study design, data collection and analysis, decision to publish, or preparation of the manuscript.*<br>• **YES** - Specify the role(s) played.<br><br>* typeset	BM. 1 grants (2014-18), Forest Health Department, French Ministry of Agriculture and Forestry<br>https://agriculture.gouv.fr/le-departement-de-la-sante-des-forets-role-et-missions<br>The funder participated in the data collection, in particular for plots in Brittany and Charente.<br>The funder had no role in study design, analysis, decision to publish, or preparation of the manuscript<br><br>BM. 1 grants (2018-21) H2020 HOMED.<br>https://homed-project.eu/<br>The funder had no role in study design, data collection and analysis, decision to publish, or preparation of the manuscript.
**Competing Interests**	NO authors have competing interests

Use the instructions below to enter a competing interest statement for this submission. On behalf of all authors, disclose any competing interests that could be perceived to bias this work—acknowledging all financial support and any other relevant financial or non-financial competing interests.<br><br>This statement **will appear in the published article** if the submission is accepted. Please make sure it is accurate. View published research articles from *PLOS Pathogens* for specific examples.<br><br>**NO authors have competing interests**<br><br>Enter: *The authors have declared that no competing interests exist.*<br><br>**Authors with competing interests**<br><br>Enter competing interest details beginning with this statement:<br><br>*I have read the journal's policy and the authors of this manuscript have the following competing interests: [insert competing interests here]*<br><br>* typeset<br><br>This statement is required for submission and **will appear in the published article** if the submission is accepted. Please make sure it is accurate and that any funding sources listed in your Funding Information later in the submission form are also declared in your Financial Disclosure statement.	
**Data Availability**<br><br>Authors are required to make all data underlying the findings described fully available, without restriction, and from the time of publication. PLOS allows rare exceptions to address legal and ethical	Yes - all data are fully available without restriction

Powered by Editorial Manager® and ProduXion Manager® from Aries Systems Corporation

concerns. See the PLOS Data Policy and FAQ for detailed information.<br><br>A Data Availability Statement describing where the data can be found is required at submission. Your answers to this question constitute the Data Availability Statement and **will be published in the article**, if accepted.<br><br>**Important:** Stating 'data available on request from the author' is not sufficient. If your data are only available upon request, select 'No' for the first question and explain your exceptional situation in the text box.<br><br>Do the authors confirm that all data underlying the findings described in their manuscript are fully available without restriction?	
**Describe where the data may be found in full sentences. If you are copying our sample text, replace any instances of XXX with the appropriate details.**<br>• If the data are **held or will be held in a public repository**, include URLs, accession numbers or DOIs. If this information will only be available after acceptance, indicate this by ticking the box below. For example: *All XXX files are available from the XXX database (accession number(s) XXX, XXX.*).<br>• For research data submitted to Dryad, enter the following: *The data is available from Dryad repository DOI: XXX*.<br>• If the data are all contained **within the manuscript and/or Supporting Information files**, enter the following: *All relevant data are within the manuscript and its Supporting Information files.*<br>• If neither of these applies but you are able to provide **details of access elsewhere**, with or without limitations, please do so. For example:<br><br>*Data cannot be shared publicly because of [XXX]. Data are available from the XXX Institutional Data Access / Ethics*	Data will be placed on Dryad repository upon acceptance

Committee (contact via XXX) for researchers who meet the criteria for access to confidential data.<br><br>*The data underlying the results presented in the study are available from (include the name of the third party and contact information or URL).*<br>• This text is appropriate if the data are owned by a third party and authors do not have permission to share the data.<br><br>* typeset	
Additional data availability information:	Tick here if the URLs/accession numbers/DOIs will be available only after acceptance of the manuscript for publication so that we can ensure their inclusion before publication.
**Government Employee**<br><br>Are you or any of the contributing authors an employee of the United States government?<br><br>Manuscripts authored by one or more US Government employees are not copyrighted, but are licensed under a CC0 Public Domain Dedication, which allows unlimited distribution and reuse of the article for any lawful purpose. This is a legal requirement for US Government employees.<br><br>This will be typeset if the manuscript is accepted for publication.	No - No authors are employees of the U.S. government.
**Financial Disclosure**<br><br>Enter a financial disclosure statement that describes the sources of funding for the work included in this submission and the role the funder(s) played. This includes grants and any commercial funding of the work or authors.<br><br>This statement will be typeset if the manuscript is accepted for publication.	BM. 1 grants (2014-18), Forest Health Department, French Ministry of Agriculture and Forestry https://agriculture.gouv.fr/le-departement-de-la-sante-des-forets-role-et-missions The funder participated in the data collection, in particular for plots in Brittany and Charente. The funder had no role in study design, analysis, decision to publish, or preparation of the manuscript BM. 1 grants (2018-21) H2020 HOMED. https://homed-project.eu/ The funder had no role in study design, data collection and analysis, decision to publish, or preparation of the manuscript.

Review the submission guidelines and the instructions link below for detailed requirements and guidance.	
**Competing Interests**<br><br>On behalf of all authors, disclose any competing interests that could be perceived to bias this work.<br><br>This statement will be typeset if the manuscript is accepted for publication.<br><br>Review the instructions link below and PLOS Pathogens' competing interests policy to determine what information must be disclosed at submission.	no competing interest
**Data Availability**<br><br>Provide a **Data Availability Statement** in the box below. This statement should detail where the data used in this submission can be accessed. This statement will be typeset if the manuscript is accepted for publication.<br><br>Before publication, authors are required to make all data underlying their findings fully available, without restriction. Review our PLOS Data Policy page for detailed information on this policy. Instructions for writing your Data Availability statement can be accessed via the Instructions link below.	Data are available in DRYAD repository (doi:10.5061/dryad.kh189328x)

Dear editor,

We wish to submit our manuscript “Ability of the Ash dieback pathogen to reproduce and to induce damage on its host are controlled by different environmental parameters” by Marçais et al to Plos Pathogens. We show may that *Hymenoscyphus fraxineus* can establish in environmental conditions (including climate) where it cannot induce noticeable dieback. Additionally, heathy carriers can be important in the disease epidemiology.

We believe that this work represent significant advances in the knowledge about ash dieback, a major tree pathogen affecting European forest. The impact of climate on the different step of the pathogen biological cycle are unraveled. Healthy carrier appear to play a significant role in ash dieback while this is usually not reported as important for plant pathogens. This can be critical in the context of species distribution models where climatic niche are often derived from report of significant disease impact more than just pathogen establish presence. We thus believe this can stimulate research on the epidemiology of tree diseases.

Benoit Marçais

UMR Interaction Arbres/Microorganismes, INRAE-Nancy, France

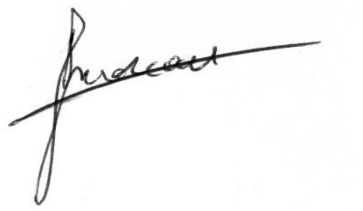

We want to thank you and reviewers for the constructive remarks on the manuscript and we introduced most of them. We tried to remove the typos mistakes as much as possible and hope there isn't much left.

In particular, reviewer 1 pointed out that material and method did not really enable to understand if the same saplings were rated over several years and how that was taken into account in the analysis. We agree this was a major problem. First, we introduced the changes you suggested for the saplings selection and rating over years making now clear that the majority of saplings were rated several years. The overall replacement rate is 18% but it has to be pointed out that the saplings replaced are mostly saplings experiencing dieback. The saplings that show no dieback do not die frequently and usually stay in the sample for all the study period. Then, in the data analysis, we realized that it was not indicated that the dieback status of saplings that is taken into account is the worst dieback rating observed on each sapling during the study period. This is now indicated L260-2. Related to that, the way the model was specified was also not correct (L252-253). The respective probability of leaf necrosis and shoot mortality pl[i] and ps[i] should be indexed on n, the shoot, i the sapling and t the year. This is now done and it can be seen that the ash dieback status DS[i] does not depend on the year t. I hope this clarify properly the methodology.

Abstract:
Line 14: I suggest, "genetic resistance or tolerance to the disease".
Done

Author summary

Having read your paper thoroughly, I think the last sentence of the Author Summary is one of the most speculative points in the paper and could be omitted. It's potentially misleading for a non-specialist reader because in the paragraph starting on line 511, you acknowledge that this could be an artefact because of a recent run of dry summers in France. If you wish to use the space to mention another important conclusion instead, please do.
Done

Short title: Perhaps, "Environmental influences on ash dieback"? You consider environmental variables other than climate.
Done

Introduction

Line 91: "very limited inoculum production occurs on diseased shoots". This is slightly confusing because "shoots" includes the leaves on those shoots. Maybe, "occurs on the bark of diseased shoots" - is that what you mean?
Done

Line 97: I suggest "fallen leaves" to avoid giving the impression that the pathogens undergoes (sexual) reproduction on green leaves.
Done

Line 100: I suggest something like, "could prevent transfer of the pathogen from infected leaves to shoots but still allow sexual reproduction on fallen rachises". Also see lines 255-258.

Done

Lines 109-114: I feel the first sentence of this paragraph suggests that the emphasis of the paper is on discovering if ash trees are tolerant, while "studied environmental influence" is imprecise. Your data are indeed relevant to tolerance but this is a consequence of what you have discovered about environmental effects on different stages of the disease. I suggest rephrasing line 110 to, "we tested if different environmental variables favour important stages..." I also suggest deleting the last sentence of the paragraph (the key steps of the H.f. life cycle have already been characterised) and replacing it by something like, "We propose that healthy carriers, particularly tolerant genotypes of ash, could be favoured in locations where the environment promotes infection and reproduction of H.f. but not symptom development".
Clarified

Materials and methods

This is mostly fairly clear but some points could be clarified or made more precise.

Line 120: I suggest, "where ash dieback was first observed in 2009-10" (given that the disease may have been present for many years previously; note Wylder et al. 2018).
Done

Line 126: Does "tree cover" mean all trees or only ash? I believe you mean all species but please make this clear.
Done

Line 131: Was there any difference between saplings and "larger trees with low-lying branches"? Could it have influenced variation between plots?
We tried at one point to test whether the tree height could be a suitable co-variable and it did not stick out as significant. This is now added L132-133 without detail information.

Line 133: When you say, "without dieback symptoms", do you mean there were no dead twigs or shoots and you discounted lesions on leaves? (Noting that you go on to describe your rating system for saplings in terms of the proportion of dead wood.) If so, I suggest, "without dieback of shoots or twigs".
See later. Dieback just refers to perennial organ such as shoot and discount leaves (L136-7).

Important point: Lines 167-8: "we were mainly interested in the climatic conditions during rachis colonisation phase in June –October": does this mean you assumed that environmental conditions were not important during the overwintering stage (do we know what happens at that time - pseudosclerotia maturing, perhaps?) or in spring, when ascomata are developing? Similarly, lines 168-170: You used a standard set of conditions to produce apothecia in April and May, but surely local conditions during this time could be important in the life cycle of H.f.? Your decision to focus on conditions from June to October needs more justification than simply "we were interested".

We focused on the vegetation season because this is the time were inoculum production and leaf infection occurs and we presumed that it would be the main factor affecting latter rachis

colonisation. This is now indicated in the text (L173-175). Of course, conditions during winter could influence the production of the pseudosclérotiale plate and thus have some importance. But, this would have added a travel to plots scattered over France. We did not have the resources to do that and had to limit our ambition… Then for the fructification assay, what is tested here is the potential of rachises to produce apothecia if they are in suitable conditions. We could not test everything in one study!

The point about rating samplings over 1-6 years and the important point about replacing saplings (lines 154-158) is part of the study design. I therefore suggest moving those three sentences to the earlier paragraph where you describe the selection of saplings, e.g. to line 135. I agree with Reviewer 1 that it is not clear if you rated individual saplings over 1-6 years (I think that's what you did but it isn't totally obvious) or if you used a different selection of saplings from each site in each year. This is easily fixed by changing "Saplings were rated" to "Each sapling was rated" on line 154. I also agree with the reviewer that your description of how replacement saplings were chosen is not as clear as it should be. On line 157, when you say "Dead saplings were replaced", were they replaced by the closest appropriate sapling, or by another sapling from somewhere within the same plot, or what? And on line 158, I suggest, "tolerant or resistant to H.frax". You could also say that only 1.5% of saplings in total were replaced over the study period (as I understand from your response to the reviewers of the original version), which indicates that this doesn't affect the majority of the data.

Done. More saplings where replaced in fact, although it still represent a minority. We computed that the mean replacement rate was of 18%. This is because *H. fraxineus* induce a high mortality rate at the sapling stage and cannot be avoided. This is now indicated in the text (148-149).

Line 295 (and elsewhere): Did you use a procedure to avoid for Type I errors in multiple significance tests?

Altogether, we tested only 5 models (comparison of 3 periods in spring for leaf necrosis frequency and of 2 in autumn for shoot mortality) to find the most suitable periods and did not account for Type I error. After, all variables were entered in the models. We simplified the presentation, as it was unnecessary to add a step in frequentist method. In the revision, we compare 5 Bayesian models with the BIC and hope it is much more clear now (L315-319).

Results

I appreciate that you have taken the approach of reporting the results concisely and factually, with little interpretation, which is good practice. However, there are many points where the text is unclear or ambiguous. Please consider the following suggestions for improvement.

Line 337: "very few leaf necrosis": To be clear, did you score the existence or absence of necrosis on a leaf as a categorical variable with two states? Or did you score the number / proportion of leaflets with necrosis within a leaf, or the area of necrosis? If you just scored presence / absence, a point for the Discussion is whether or not this is an accurate measure of disease development on an ash sapling.
We scored the leaf necrosis as a present / absence of necrosis on a leaf without quantifying the proportion / number of leaflet with necrosis. This is now indicated in L151-2. Then, we are not sure adding a long discussion thread on the way to measure foliar lesion is adequate as the

manuscript is already complicated as you pointed out. We however added a sentence in the discussion L473-5. The number of leaflet affected may not be the real issues as lesions can be close/far from the stem or even directly on the rachis. It is a question of trade off. More detailed data is often desirable but usually come at the expenses of number of replicates. This is a very general debate...

Lines 339-340: This sentence doesn't make sense. Do you mean that the frequency of the presence of H.f. in leaves is similar to the visual estimate of the presence of leaf necrosis when necrosis is very frequent, but higher when necrosis frequency is low?

It is in fact the contrary. We deleted the sentence, as it appeared unclear. What was meant is that we make no mistake when we assess that H.f. induced abundant leaf necrosis and when we assess that H.f. induced little leaf necrosis, it is likely even more the case. The preceding sentence (L340-342) should indicate this clearly enough.

Lines 342-343: I don't understand how the percentage of leaves with necrosis can be similar on a plant with a dieback score of 0 or 1 ("complete absence of dead twigs" or "less than 10% dead twigs") and on one with a dieback score of 3 ("more than 50% of dead twigs"). Are you including the dead twigs in the assessment of % leaves with necrosis? If not (I can only assume you aren't), do you mean that the percent of leaves which are partly necrotic but still partly green is the same on trees with no shoot dieback or extensive shoot dieback? Or was the frequency of necrosis so high that almost all leaves had some necrosis, even on plants with little shoot dieback? At any rate, some major clarification or explanation of this point is required. Referring to my previous point, if the frequency of leaves with necrosis is high even on plants with a low dieback score, this indicates that the necrosis frequency has little power to discriminate healthier and sicker trees, and that a more quantitative score such as the frequency of necrotic leaflets or the area of necrosis would be more precise.
The term “dieback” just include shoot mortality. We added this in lines 136-7 to be clear. Indeed, defoliation by insects or leaf pathogens that are not chronic usually do not lead to dieback. This is what is occurring in our case. Then the rated shoots usually show no mortality in the autumn assessment in September. The shoot mortality occurs later, in December-January and mainly in February-April. So, no shoot mortality affect the frequency of leaf with necrosis. We thus indeed meant that the percent of leaves which are partly necrotic but still partly green is the same on trees with no shoot dieback or extensive shoot dieback. It has to be also pointed out that the bulk of leaf symptom observed is leaves which are partly necrotic but still partly green. Leaves can be occasionally completely necrotic, but this is not very common. We added that in the text (L345-347). Altogether, about 40-50% leaves have some partial necrosis as can be seen on figure 2. We hope that the paragraph is now easier to understand for readers not familiar with ash dieback. As we did no quantify leaf necrosis in more detail, we cannot prove that the “real” leaf necrosis severity on ash trees that show no or little shoot mortality is exactly the same as on those that show heavy shoot mortality. This was however our field impression. It remains highly speculative and not a major point in the manuscript. A point in discussion was however added L473-5.

Line 346: "the plot * dieback status interaction was not significant" - I appreciate that this may be an accurate report of a statistical test but it's one of several places where the biological relevance of the statistical result is unclear. Do you mean that the frequencies of different dieback scores were similar in different plots?
We rephrased the paragraph and hope it is now clearer

Lines 352-353: "The likelihood of shoot infection was significantly higher in [trees?] with severe dieback than in those with no dieback". As written, this seems trivial but do you mean that the likelihood of infection of new shoots was significantly higher in trees with more severe dieback?

First, we apologize, there was a mistake. What was meant is that the observed shoots had a mortality rate higher on saplings with dieback versus those not showing dieback. So it was not “shoot infection”. We rephrased the sentence and hope it is now clearer. Then it may be misleading to place it at the end after we report the overall dieback status of the sapling in 2015 and we moved the sentence.

Line 355: "Shoot mortality in spring was strongly related to leaf necrosis likelihood". Unclear: why 'likelihood of necrosis'? Why not just the amount of necrosis at the end of the previous summer? A similar comments applies to the sentences on lines 357-8 and 358-60.

The wording was changed for the 2 first sentences. However, I did not understand the problem with sentence L358-360.

Line 360: "leaf necrosis did not significantly depend on tree dieback status". This looks like the same point as on lines 342-3 but the statistics are different. Clarification needed.

L342-3 in the section “Ability of *H. fraxineus* to complete its cycle on ash saplings with different dieback severity” refers to a data set including only data from 2 plots (Gremecey and Seichamps) in 2014 autumn while the next paragraph (“Relationship between leaf necrosis in fall and subsequent shoot mortality”, L360) refers to the entire data set (55 plot*year combinations). We added a sentence in the first section to clarify that (L344).

Line 367: "affected by tree cover". In which direction? (I can see it in Fig. 3 but please say in the text.)

Done

Lines 394-5: "shoot mortality was strongly linked to leaf necrosis likelihood". (a) In which direction? (As for line 367.) (b) Why 'necrosis likelihood'? (As for line 355.)

Corrected

Line 395: "shoot mortality ... increased with increasing tree cover". This looks like the same point as you made on line 367. Clarification needed.

It is indeed the same phenomenon that was included in the plot level model as well as in the shoot level model because of its importance. It is here not that important to recall it and it was deleted.

Lines 415-6: "rachises producing apothecia ... not significantly related to ... leaf necrosis". In Table 4, there's a very large (negative) parameter relating apothecia to necrosis which isn't significant (P=0.3) but a much smaller (positive) parameter relating apothecia to rainfall, which *is* significant (P=0.006). Are you able to give a reason for this apparent disparity?

This is because in this analysis the variables were not standardized (mean=0, sd=1). So the large parameter value relating apothecia to leaf necrosis reflects that leaf necrosis frequency is in [0, 1] while the large parameter value relating apothecia to rainfall reflects that rainfall is in [40,120]. For consistency, we changed the analysis to show the result with standardized variables.

Discussion

Having written the Results section in a concise manner with little or no interpretation, it would greatly help readers to understand and evaluate the conclusions of your research if you referred to your figures and tables throughout the Discussion. The study is so complex with many explanatory variables which interact to affect three outcome variables differently (necrosis, mortality, apothecia), and have somewhat different effects at many locations, that it's very challenging to follow the Discussion without a clear guide as to which piece of data is relevant.

For example, your very first point in the Discussion, "healthy carriers exist": this requires the reader to piece together results from several tables and figures. There is some explanation in the next sentence but it would help readers greatly if each point in that sentence had references to the relevant data.
Done. We tried to refer to figures and tables throughout the discussion to help the readers.

A specific point is that your paper is of at least as much interest to plant pathologists as to ecologists but, unlike ecologist, few pathologists are familiar with bayesian statistics. Without more exposition of your conclusions, I frankly believe that most readers will find the Discussion very difficult to follow in detail. I'm familiar with bayesian methods but I myself found it pretty tough.
Looking back at my notes on the Discussion, most of them just say "Explain" - I think that gives you a flavour of what's needed!

This is a very difficult remark to take this into account as we have no ideas of where explanation is needed … We tried to refer to figure and table in the discussion section to help the reader to follow the discussion and hope that this help.

Specific points in the Discussion:

Line 438: Do you have any information which would allow you to extrapolate to other regions, beyond France?
We did not apply the model to other regions of Europe and thus have no precise information for elsewhere. I would expect that this might apply in many places on the southern limit of the disease, but in absence of detailed information… A sentence was added to say it might apply elsewhere (L446-7).

Line 446: Likewise, just in French conditions? From observation, I think this is true in the UK because I've very rarely seen apothecia even in early September. If there's hard evidence for this outside France, please give a reference.
The sentence was to explain was is observed in this study and thus refers to France. More information is available in other countries from spore traps. I am not aware of work in UK, but data exist in the Cech republic and Norway. Dvorak et al (2016) detect airborne inoculum in September-October and Timmermann et al (2011) detect some in September.

Line 492: You say that at low tree cover, crowns are exposed more frequently to adverse temperatures. Could there also be more wind or a dryer micro-environment, both of which are unfavourable to H.frax? (Extrapolating Chumanova's conclusions to the level of individual trees.)

we extended to other micro-climatic conditions known to be different under closed canopies (humidity)

Line 522-3 (sentence about microbiota): Interesting but rather speculative - omit?
done

Lines 543-5: I don't understand this sentence. I understand that infection can be promoted by rain but necrosis can be suppressed by high temperatures. But why does this explain why rainfall isn't correlated with symptoms, given that high summer rainfall usually involves lower temperatures in summer (but higher in winter)? Or doesn't the correlation between rainfall and lower temperature in summer apply so much in France?
In the study period, some years had adequate rainfall while summer temperature where very high. This is the general trend for many mid-latitude areas in Europe: the climate change with higher summer temperatures while summer rainfalls do not decrease. We now refer to Table 3 that show that this is what happened in the Champenoux area in 2019 (L560-3).

Lines 558-60: "H. fraxineus ability to infect ash leaves and reproduce on the rachises in the forest litter does not appears to limit the pathogen ability to establish throughout France. By contrast, the ability to induce shoot mortality is the limiting step." This is another point I don't understand because a key point of this paper is that reproduction of H.frax on fallen rachises is not closely connected with shoot mortality. Why then do you say that shoot mortality is a limiting step in the establishment of the pathogen? Especially because in Figure 7, you say that shoot mortality is a dead end for the pathogen.
Sorry, the wording was misleading. You are right. What we meant to say is that poor ability to induce shoot mortality explains the absence of disease report in southern France. We changed the sentence to clarify.

Tables 1 and 2: Please say that positive values indicate an association between the higher levels of the symptom and higher values of the environmental variable.
done

Figure 2C: On the y-axis, do you mean % shoots with *leaf* necrosis in spring 2015?
No, this is the % dead shoot in spring 2015. Corrected.

Reviewer #2:

L86: replace where with when
L210: "an engine" should probably be replaced by a better term, e.g. logging machinery?
Actually, the plot was partially destroyed in a house building operation (digging a pit for soil assessment). I do not really know what would be the good term.

L227: remove: "this is a common procedure frequently used in logistic regression."
Fig 2 could be improved by adding p.values
done

The quality of all figures in the latest submission seem to be very low (pixelated/unfocused/not sharp) they may need to be generated at higher resolution.

L370: please elaborate on "As no meso-climate effect was included in this model, this was

expected." in line 254 you state that "The Env.l [i] and Env.s [i] random factors were taken as proxies for the environment, combining meso/micro climate and plot conditions (host density, Infection history)." both Env factors seem to be in the model (table 1).
done

373-373: unclear sentence: "with increasing shoot mortality with increasing defoliation level"
clarified

380-381: BIC values for "shoot mortality vs climate" model are the same for both time frames. Is that correct?
clarified

401-404: improve/clarify English. Use "Moreover, it was not linked to ... " rather than "Moreover, it was
significantly linked neither to ..."
done

432: "carrier trees without crown dieback symptoms" would be better than "healthy carrier"
done

436-438: "...it should not be able to induce significant dieback in large part of southern France." I feel the conclusion is a bit too strong given the variation of modelling outcomes in fig S2. More nuanced wording reflecting the effect of parameters presented in Fig S2 is required.
done

454: change capital "High" --> "high" also be more specific and clearly state that high levels of "leaf necrosis" rather that "levels" are not required for rachis colonisation.
done

455: "this would explain the observed result." feels out of place
Clarified

459:"within soil reach" better description of the sampling position required.
Clarified

461: "more humid close to soil and thus more favourable to H. fraxineus infection." (reference missing)
511-523: could this also be due to natural selection of less virulent local strains of the pathogen?
As we removed the reference to microbiome evolution as “too speculative”, we feel that adding a reference to selection of less virulent strain would also be so. Then, no proof of virulence evolution in H. fraxineus between long ago colonised area and new foci was found by Lygis et al (2017, 10.1111/ppa.12635).

534-538: split in 2 sentences please.
Done

556-557: reword "was by now never reported" to "This is the first report of..."
Done

figure 7 is a great addition but could be improved. Changing red and blue colour (perhaps as a scale) to designate only positive (shades of red) and negative (shades of blue) effects of the studied parameters rather than different parts of disease cycle would convene the gist of the study more clearly. The different parts of the disease cycle could use black and white arrows instead.
We tried and feel it does not improve the figure (some factor such as tree genotype are not +/- and is is difficylt to label differently the different part of the cycle outside the arrows). We thus slicked to the original figure.

Similarly the Fig 7 caption could better describe the actual dynamics of the model and try to avoid ambiguous terms such as "moderate", "presumably" which distract from the point of the figure. I propose the following caption format: "Higher summer rainfall and higher spring temperatures are associated with higher rates of leaf necrosis leading go higher shot necrosis especially in high tree cover. Lower summer temperature .... "
The caption was adapted as suggested

Manuscript Click here to access/download;Manuscript;Inf_foliaire_S3.docx

Ability of the ash dieback pathogen to reproduce and to induce damage on its host are controlled by different environmental parameters

Benoit Marçais[1*], Arnaud Giraudel[1], Claude Husson[2]

[1] Université de Lorraine, INRAE-Grand-Est, UMR1136 Interactions Arbres/Microorganismes, F-54000 Nancy, France

[2] Département de la Santé des Forêts, Ministère de l'Agriculture et de la Souveraineté Alimentaire - DGAL , F-75015 Paris, France

* Corresponding author

E-mail: benoit.marcais@inrae.fr

Abstract

Ash dieback, induced by an invasive ascomycete, *Hymenoscyphus fraxineus*, has emerged in the late 1990s as a severe disease threatening ash populations in Europe. Future prospects for ash are improved by the existence of individuals with natural genetic resistance or tolerance to the disease and by limited disease impact in many environmental conditions where ~~ashes are frequent~~. Nevertheless, it was suggested that, even in those conditions, ash trees are infected and enable pathogen transmission. We studied the influence of climate and local environment on the ability of *H. fraxineus* to infect, be transmitted and cause damage on its host. We showed that healthy carrier, i.e. individuals showing no dieback but carrying *H. fraxineus*, exist and may play a significant role in ash dieback epidemiology. Environment strongly influenced *H. fraxineus* with different parameters being important depending on the life cycle stage. The ability of *H. fraxineus* to establish on ash leaves and to reproduce on the leaf debris in the litter (rachises) mainly depended on total precipitations in July-August and was not influenced by local tree cover. By contrast, damages to the host, and in particular shoot mortality was significantly reduced by high summer temperature in July-August and by high autumn average temperature. As a consequence, in many situations, ash trees are infected and enable *H. fraxineus* transmission while showing limited or even no damages. We also observed a decreasing trend of severity (leaf necrosis and shoot mortality likelihood) with the time of disease presence in a plot that could be significant for the future of ash dieback.

Author Summary

Ash dieback is an important tree disease induced by *Hymenoscyphus fraxineus* an invasive fungus. We studied the influence of climate and local environment on the ability of *H. fraxineus* to infect, be transmitted and cause damage on its host. We showed that healthy carriers, i.e. individuals showing no dieback but carrying *H. fraxineus*, exist and may play a significant role in ash dieback epidemiology. While the ability of *H. fraxineus* to establish and to reproduce on leaves mainly depended on summer rainfall, the damages it causes to the host depend on other parameters (high summer temperature in July-August, tree cover). As a consequence, in many situations, ash trees are infected and enable *H. fraxineus* transmission while showing limited or even no damages. This is in particular the case of non-forest situations such as hedges and isolated trees.

Keywords: *Hymenoscyphus fraxineus*, climate, healthy carrier, latent infection, apothecia production

Short title: Environmental influence on ash dieback

Introduction

The existence of healthy carriers, i.e. infected individual that remain asymptomatic, is not recognized as an important feature in plant epidemiology (Cunniffe *et al*, 2015). The existence of healthy carriers has especially been reported for weak pathogens or those that can induce damage only on stressed hosts (so-called latent pathogens, Sieber, 2007). This is known to be a significant feature for important stress-induced tree diseases such as *Diplodia* shoot blight, sooty bark disease of maples or *Botryosphaeria* cankers (Flowers *et al*, 2001, Marsberg *et al*, 2017, Kelnarová *et al*, 2017). However, high production of inoculum usually occurs only at symptoms onset. By contrast, the existence of healthy carriers is seldom reported for primary pathogens that do not need host stress to induce significant host damages. Tolerance has been reported as a mechanism used by plants to cope with infection (Pagán & García-Arenal, 2020). This mechanism postulates that some individuals will limit the impact of infection on their fitness while sustaining significant pathogen load and enabling pathogen multiplication. However, the presence of individual plants that provide important levels of inoculum while exhibiting limited or even no visible symptoms is seldom reported. Disease severity is usually assumed to correlate positively with the multiplication of the pathogen within the host (Alizon & Michalakis, 2015). Nevertheless, at a different level, when comparing different species, it has been shown that poor correlation may exist between vulnerability (reduction of fitness caused by the infection) and competence (ability to sustain the epidemic by providing large amounts of inoculum). A striking example is given by Sudden Oak Death that developed in California in the last decades: while this disease, induced by *Phytophthora ramorum*, affects primarily *Fagaceae*, killing *Quercus* and *Lithocarpus* species, the main inoculum producer is a laurel, *Umbellularia californica* which coexist in natural forest and does not appear to suffer much fitness reduction (so-called reservoir host, Davidson *et al*, 2005, DiLeo *et al*, 2009, Garbelotto *et al*, 2017).

Such a discrepancy between pathogen’s ability to establish in a stand and to produce inoculum and its ability to induce damages is an important difficulty for assessing the climatic niche of a pathogen. This assessment is a critical step to evaluate the risk associated with pathogen occurrence (Holdenrieder *et al*, 2004). The most widespread approach to develop species distribution models (SDM) is to relate the species known occurrence to environmental parameters, in particular climate, through statistical analyses (Elith & Leathwick, 2009). If large discrepancies exist between a pathogen presence and its ability to cause serious damage, this will lead to important under-reporting and to poor available data. By contrast, mechanistic SDM, which relies on the assessment of environment influence on the different stages of the pathogen cycle (Dormann *et al*, 2012) could be a suitable alternative to assess the climatic niche of a pathogen in such conditions.

Hymenoscyphus fraxineus is an invasive pathogen that has caused ash dieback throughout Europe since the late nineties and has jeopardized *Fraxinus excelsior* stands (Gross *et al*, 2014). *H. fraxineus* fulfils its life cycle on ash leaves. Those are infected by ascospores during late spring and summer and remain asymptomatic until late August when necrotic leaf lesions develop (Cross *et al*, 2017). *H. fraxineus* then colonises the leaf petiole and may infect young shoots, inducing dieback (Gross *et al*, 2014). Most of the inoculum production occurs on leaf debris in the forest litter (so-called rachis that encompasses the petiole and the main vein of the composite leave, on which fruit bodies i.e. apothecia develop, Gross *et al*, 2014). By contrast, very limited inoculum production occurs on bark of diseased shoots which is considered to represent a dead-end for *H. fraxineus* (Landolt *et al*, 2016). It has been suggested that, in some conditions, *H. fraxineus* may reproduce on ash leaves without inducing significant dieback. Some *F. excelsior* individuals are not affected by *H. fraxineus*, even under high inoculum

pressure (McKinney *et al*, 2011). These individuals do not sustain extensive lesions when artificially inoculated at the shoot level (McKinney *et al*, 2012). It was then suggested that their fallen leaves could nevertheless sustain the pathogen reproduction and that the mechanism involved in containing the disease might be tolerance instead of resistance (Landolt *et al*, 2016). However, no experimental data is yet available to sustain this claim. Another reported tolerance mechanism would be early abscission that could prevent transfer of the pathogen from infected leaves to shoots but still allow sexual reproduction on fallen rachises (Mc Kinney *et al*, 2011, Schwanda & Kirisits, 2016, Orton *et al*, 2019). Also, environmental conditions may influence differently dieback and colonisation of the rachises in the litter. Grosdidier *et al* (2020) showed that in open canopy conditions, the infection levels of ash rachis by *H. fraxineus* in the litter was similar compared to forest stands with a close canopy despite much lower levels of crown dieback. Those data would suggest that healthy carriers could exist in the *F. excelsior* population, with individuals able to be infected in specific conditions at the leaf level and thus able to produce inoculum while sustaining very limited to no dieback.

We thus examined whether ash trees could in some conditions behave as healthy carriers. More specifically, we tested if different environmental variables may favour important stages of ash dieback, namely the ability to colonise leaf rachises and reproduce on them and the ability to induce damage on ash trees, either leaf necrosis at the end of the summer or subsequently shoots mortality. We propose that ash behaving as healthy carriers could occur in locations where the environment promotes infection and reproduction of *H. fraxineus* but not symptom development..

Material and methods

Sampled plots

We selected 20 plots on a temperature gradient in France (Fig 1, Table S1) in order to have a range of summer temperature as large as possible within the range *of H. fraxineus* presence. The plots were usually studied several years in a row starting from 2013 in the area where ash dieback was first observed in 2009-10 (NE France) to 2020 in an area just recently affected by ash dieback (SW and NW France, in Brittany and Pyrenean Piedmont). The studied plots were either in forest settings or located in hedges. The ash species present was *Fraxinus excelsior* in all plots. A summary of observations of leaf necrosis and shoot mortality on the plots is given in table S1. Plots were characterized by their tree cover from aerial photographs (Orthophoto with 50cm pixels from IGN, http://professionnels.ign.fr). The tree cover polygon[illegible] within a radius of 100 m from each plot, including all tree species, were extracted and th[illegible] proportion of tree cover within that range (TC) was computed.

Survey procedure

In all studied plots, 20 ashes were marked. In each plot, we selected suitable saplings that were contiguous, marking all th[illegible] with two shoots available for rating. We usually selected saplings 2-4m high, although occasionally, larger trees with low-lying branches were selected. Preliminary analysis showed that the tree height did not influence leaf necrosis and shoot mortality likelihood. Saplings of different health conditions were usually present in the plots, with a proportion of saplings without dieback of shoots varying from 20 to 75% mostly depending on the length of ash dieback presence in the area. This proportion was adequate for the hypothesis we wanted to test. Dieback only refers to the mortality of perennial organs, here the shoots; symptoms occurring on the leaves are not included. The saplings were rated annually for their crown status according to five dieback scores: 0 for complete absence of dead twigs,

1 for less than 10% of dead twigs, 2 for 10 to 50% of dead twigs, 3 for more than 50 % of dead twigs and 4 for dead saplings (Grosdidier *et al*, 2020). Two annual shoots per individual were marked for rating of leaf and shoot infection. We selected the terminal shoot of the 2 lower branches that could be reached by an observer from the ground. As most sapling that were 2-4m high, this should not introduce any bias. Each sapling was rated for several years in row (1 to 6 years depending on the sites and on the year of first ash dieback report, Table S1). Dead twigs were replaced by the nearest shoot available from the lower part of the sapling. Dead saplings were replaced by the closest appropriate sapling, taking care to select saplings with ash dieback symptoms to avoid ending with only individuals potentially tolerant or resistant to *H. fraxineus*. On average, about 18% of the saplings were replaced over the study period, mostly saplings showing dieback as they suffered high mortality.

The necrosis induced by *H. fraxineus* on leaves was rated in September. The total number of leaves present on each marked annual shoot, the number of leaves with lesions (not taking into account the number of affected leaflet) and the number of leaf scars indicating defoliation were determined (altogether 4 to 27 leaves depending on the shoot). Lesions induced by *H. fraxineus* can be recognized as brown lesions starting at leaf margin and extending preferentially on leaf veins. Leaves presenting brown lesions only on the rachis were also rated as infected. Two leaves rated as presenting *H. fraxineus*-induced lesions were sampled for further analysis in the laboratory in 2018-20. Sampled leaves were stored individually in paper bags and kept at ambient temperature. In the laboratory, they were surface sterilized (5 min in 4% NaOCl and 1 min in 90% ethanol) and analysed by qPCR for the presence of *H. fraxineus* as described by Ioos et al. (2009). In the following year's vegetation season, the status of marked shoots was assessed as living or dead. Whenever the shoot had wilted because of girdling occurring below the annual shoot, we considered that it was not possible to determine whether infection had transferred from the annual shoot leaves to the stem and the data was counted as missing.

In October-November, 20 rachises that had just fallen to the ground were sampled by site in order to assess the ability of *H. fraxineus* to produced apothecia on them (called hereafter fructification assay). The rachises were sampled at the base of the marked saplings (one rachis per sapling). This was done in all sites of Champenoux (2016, 2017, 2019, 2020), Fréchencourt (2016, 2017), Lupé (2016), Roche-sur-Grane (2017) and in the 4 Pyrenean sites (Ol1, Ol2, Fe, Be, 2020). The rachises were placed in mesh bags and overwintered in a common garden in a forest site in Champenoux (6.34251, 48.75297, EPSG 4326) except for the rachises of the plots close to Pau that were overwintered close to the site of Sarrance (-0.59692, 43.00044, EPSG 4326). This was done to minimize travel; climatic conditions during the vegetation season conditions determine inoculum production and leaf infection and we supposed that it would be the main factors controlling colonisation of the rachises. In the following spring, in April or May depending on the year, the rachises were retrieved from the mesh bags and were placed for 6-8 weeks at 18-20°C in humid chambers in large plastics boxes in contact with moistened filter paper to assess their ability to produce apothecia. The moistened filter paper was kept wet throughout the incubation and was changed weekly. Whenever a rachis produced *H. fraxineus* apothecia, the rachis part covered with pseudosclerotial plate was removed and stored in paper bags for further analysis. The part without pseudosclerotial plate was left in the humid chamber. *H. fraxineus* apothecia can be recognized as 2-4 mm white apothecia with a dark base of the pedicels and smooth margin of the fruiting disk (Kowalski & Holdenrieder, 2009). The monitoring was stopped whenever no new production of apothecia was observed in a week (after 6-8 weeks incubation). Remaining rachises were then placed in separate bags. The amount of rachises parts producing / not producing apothecia was determined by measuring the

rachis length in 2016 and 2017 and by measuring their weight after drying 3d at 50°C in 2019 and 2020. The proportion of rachises producing apothecia was then computed.

In 2019 and 2020 in sites in Champenoux and in 2020 in Pyrenean sites, 10 additional rachises were collected per site at the same time at leaf fall to determine the frequency of *H. fraxineus* by qPCR. Individual rachises were kept separate. They were dried 3d at 50°C, cut in small 1-2 mm sections and grinded with a mortar. A subsample of 50 mg per rachis was then used for DNA extraction with DNAeasy Plant Mini kit (Qiagen). AP1 lysis buffer (800 µl), 4 µl RNase, two 3-mm and twenty 2-mm glass beads were added to the tube and the sample was grounded twice using a beadbeater (Tissue Lyser, Qiagen) at 30 Hz for 30 s. The DNA extraction was then conducted according to the manufacturer's recommendations. Total DNA was eluted in 150 µl AE buffer. The samples were then analysed by qPCR for the presence of *H. fraxineus* as described in Ioos et al. (2009).

Ability of *H. fraxineus* to complete its cycle on ash saplings with different dieback severity

The ability of *H. fraxineus* to complete its cycle on ash saplings with no dieback symptoms was studied in two of the plots, Gremecey and Seichamps. Ash saplings about 2-3 m high were selected during summer 2014 in order to have 10-13 saplings of two types in each stand: i. saplings with either no or only very few scattered small dead shoots (no dieback, crown status of 0 or 1), ii. saplings strongly affected, with top shoots killed by *H. fraxineus* (crown status of at least 3). The two stands were densely stocked naturally regenerated ash stands heavily and uniformly infected by *H. fraxineus* since at least 4 years. The saplings without dieback were in close vicinity to individuals with severe dieback, with usually several severely affected saplings within less than one meter from the healthy-looking sapling.

On each sapling, an annual shoot with 7-28 leaves was selected and marked at the end of July. The selected shoot infection by *H. fraxineus* was rated at the beginning of September 2014 with record of the number of leaves with necrosis and total number of leaves. To measure the ability of *H. fraxineus* to complete its cycle on rachises, we placed a net around the entire crown of each sapling in mid-September to collect all the leaves falling during the autumn. The nets containing the leaves were retrieved at the end of October and placed on the ground outdoors in a nursery close to both plots to overwinter. Unfortunately, logging occurred in the Seichamps plot during autumn and an engine destroyed many studied saplings. Only four saplings without dieback and one with severe die[illegible]k were available in that plot for the net harvest. In April 2015, the rachises were retrieved from the 27 nets (11+4 without dieback and 11+1 severely affected by *H. fraxineus*) and were placed in large plastic boxes in contact with moistened filter paper. The boxes were then closed and placed in the laboratory at room temperature. After a 5-week incubation, the rachises were examined for presence of *H. fraxineus* apothecia. The total length of each rachis was determined as well as the length between the two most distant apothecia of the rachis (hereafter called L.apo). As the part of rachises colonised by *H. fraxineus* was usually covered with many primordia of apothecium in the humid chamber, this is equivalent to the length of rachis colonised by *H. fraxineus*. The percentage of rachis colonisation on a sapling was determined as $\sum L.apo / \sum total\ rachis\ length$. The saplings were again assessed in summer 2015 to determine the global infection status and the presence of shoot infection on the marked shoot assessed in 2014 for leaf infection.

The likelihood of leaf necrosis in September 2014 and shoot infection in summer were assessed by logistic regression with the plot, the dieback status of the saplings and their interaction as independent variables. We used a quasibinomial distribution to analyse leaf necrosis likelihood because data showed a dispersion higher than expected for a binomial distribution (over dispersion). The percent rachis colonisation by *H. fraxineus* in spring 2015

was analysed by beta regression using the betareg package. The stand*dieback status interaction was assessed for leaf necrosis but not for shoot infection in 2015 and ability of rachises to produce apothecia as very few saplings then remained in Seichamps.

Data analysis

During survey, we noticed that some of the rated shoots were very vigorous with many rated leaves and that they were seldom infected by *H. fraxineus* whatever the general situation in the stand. These very vigorous shoots were usually sprouts from the collar of severely affected saplings. Preliminary analysis showed that shoots with more than 20 leaves had on average 5.9% necrotic leaves and no shoot mortality, compared to average values of 16.0 % of necrotic leaves and 21.6% shoot mortality for the total dataset. Shoots with more than 20 leaves were thus removed from the data set prior to analysis (represent only 1.5% of the data). Two different models were developed. Bayesian models were fitted with Jags 4.3.0 emulated with R4.1.0 (R2jags library).

Relationship between leaf necrosis and shoot mortality

The first model aimed at exploring the relationship between leaf necrosis and shoot mortality at the individual shoot level and how this may be controlled by tree health and site tree cover. Data were thus kept at the shoot level for this model. A hierarchical Bayesian model was developed to assess symptoms on shoot n of sapling i in year t:

$$\text{logit}(pl[n,i,t]) = \alpha_1 + \alpha_2 \times DS[i] + \alpha_3 \times TC[i] + Tree.l\ [i] + Env.l\ [i,t]$$

$$\text{logit}(ps[n,i,t]) = \beta_1 + \beta_2 \times DS[i] + \beta_3 \times TC[i] + \beta_4 \times DEF[n,i,t] + \beta_5 \times pl[n,i,t] + Tree.s\ [i] + Env.s\ [i,t]$$

with pl[n,i,t] and ps[n,i,t] the respective probability of leaf necrosis and shoot mortality, DS[i] the ash dieback status (either no dieback, when crown rating was of 0 or 1 or with dieback, when crown status was of 2 or 3), TC[i] the percent tree cover within a radius of 100 m, DEF[n,i,t] the proportion of missing leaves at the autumn rating, Tree.l [i] and Tree.s [i] individual tree random factors and Env.l [i,t] and Env.s [i,t], plot[i]*year[t] random factors. The Env.l [i] and Env.s [i] random factors were taken as proxies for the environment, combining meso/micro climate and plot conditions (host density, Infection history). The ash dieback status DS[i] was computed as the worst status observed on the sapling during the period of observation (from 2 to 7 years depending on the sapling). The defoliation level (DEF[n,i,t]) was assumed to influence the shoot mortality likelihood because Mc Kinney & al (2011) suggested that early defoliation may prevent the transfer of *H. fraxineus* from leaves to shoots and thus represent a tolerance mechanism.

Both pl[n,i,t] and ps[n,i,t] were assumed to follow binomial distribution. Non-informative priors were assigned to the model parameters according to a normal distribution N(0, 1e+05) or a uniform distribution U(0,100) for the standard deviation of the random factors Tree and Env. The model was fitted with a burn-in of 3000, for 50000 iterations with a thinning of 5. Three parallel chains with different initial parameter values were run and convergence was checked by a Gelman-Rubin test (R_{hat} were close to 1). The model was checked by looking at the plot of deviance residuals versus predicted values.

Relationship between leaf necrosis or shoot mortality and climate

The second developed hierarchical Bayesian model aimed at exploring the relationship between leaf necrosis or shoot mortality with climate. For that model, data were pooled at the plot * year level. Like in the previous model, shoot mortality was assumed to derive from former leaf infection and the proxy for leaf infection was taken as the leaf necrosis probability. Length of ash dieback presence in the area was included in the model to explain the likelihood of leaf

necrosis. The year of first report of ash dieback by the health survey system is available by 16 x 16 km quadrat over France from data of the health survey system (Département de la Santé des Forêts, Fig 1). The number of years since the report of ash dieback in the local quadrat was taken as a proxy for the length of the disease presence. It was considered an adequate proxy as local spread of *H. fraxineus* at the scale of a village was shown to be very fast (Grosdidier *et al*, 2020). Leaf necrosis likelihood was assumed to depend on spring and summer climatic conditions (total rainfall and average temperatures). It is known that *H. fraxineus* may not survive well during hot summers (Hauptmann *et al*, 2013). In particular, this pathogen is known to stop its growth at temperatures above 28°C and to have limited survival when temperature exceeds 36°C. To account for this, we used TX78, the average daily maximal temperature in July-August (°C). Shoot mortality was assumed to depend on autumn climatic conditions (total rainfalland average temperatures). The meteorological data used were Safran data from Météo-France. Safran data are computed on a 8 x 8 km grid over France (Quintana Seguı & al, 2008). The Safran point closest to each site was selected and daily air temperature and rainfall data were retrieved for the studied years. All predictors were normalised to obtain zero mean and one standard deviation. However, the odds ratio reported in the text are in the non-normalized scale.

The following hierarchical Bayesian model was fitted (Figueroa-Zúñiga & al, 2013):

$$logit(pl[i,t]) = \alpha_1 + \alpha_2 \times RA6[i,t] + \alpha_3 \times TM6[i,t] + \alpha_4 \times RA78[i,t] + \alpha_5 \times TX78[i,t] + \alpha_6 \times LDP[i,t] + Site.l\,[i]$$

$$logit(ps[i,t]) = \beta_1 + \beta_2 \times RA912[i,t] + \beta_3 \times TM912[i,t] + \beta_4 \times TC[i] + \beta 5 \times pl[i,t] + Site.s\,[i]$$

with pl[i,t] and ps[i,t] the respective likelihood of leaf necrosis and shoot mortality in plot i and year t, RA6[i,t] and TM6[i,t] the sum of rain and mean daily air temperature in June, RA78[i,t] and TX78[i,t] the sum of rain and average daily air maximal temperature in July-August (°C), LDP[i,t] the number of years of ash dieback presence in the area, RA912[i,t] and TM912[i,t], the sum of rain and mean daily air temperature in September, October, November and December and TC[i] the percent tree cover within a radius of 100m. Site.l [i] and Site.s [i] are site random factors, taken as a proxy for the site environment (micro climate, site conditions such as host density or Infection history).

While ps[i,t] was assumed to follow a binomial distribution, pl[i,t] was assumed to follow a beta distribution as the number of observed leaves per site was very high (from 200 to 400). Non-informative priors were assigned to the model parameters according to a normal distribution N(0, 1e+05) or a uniform distribution U(0,100) for the standard deviation of the random factors. The model was fitted with a burn-in of 10000, for 80000 iterations with a thinning of 10. Three parallel chains with different initial parameter values were run and convergence was checked by a Gelman-Rubin test (R_{hat} were close to 1).

The suitable periods for spring and autumn were selected in a preliminary analysis. The explored periods targeted either the period of ascospore production in spring or of the development of the shoot necrosis during the autumn. Several periods were tested: from 15 May to 15 June, for the 1 to 30 June or from the 15 June to 15 July for spring and September to October or September to December for autumn. The model showing the best DIC was selected.

The final model was checked by looking at the plot of deviance residuals versus predicted values and by a cross validation procedure. Data were randomly separated in 5 equal groups and the model was fitted using the data from 4 of the groups; predicted leaf necrosis frequency and shoot mortality were computed for the fifth group not used for the fit. The procedure was repeated five time and the observed value obtained by the procedure were plotted against the predicted values.

Relationship between climatic parameters and ability of *H. fraxineus* to produce apothecia in the fructification assay

The proportion of rachises producing apothecia in the fructification assay was analysed by beta regression using the glmmTMB library. The site was included as random factor. Fixed factors were the proportion of leaves with necrosis at the September rating, climatic parameters in the studied sites during the summer (total rainfall and mean of the daily maximal temperature in July and August) and climatic parameters during the overwintering period (average temperature and total rainfall at the overwintering locations from the beginning of October to the end of March).

Results

Data were available for 55 plot*year combinations for leaf necrosis frequency. Less data were available for shoot mortality (44 site*year combinations) because some plots were lost during winter (logging, 4 plots) or were not rated in spring (Table S1). Altogether, *H. fraxineus* was detected by the qPCR assay in 86% of the 63 tested leaves with necrosis. However, the result depended on the plot and year, with lower detection frequency in plots with very few leaf necrosis (75% of tested leaves with necrosis when overall leaf necrosis frequency was lower than 5%; 84% when it was between 5 and 15%; and 95% when it was higher than 15%).

Ability of *H. fraxineus* to complete its cycle on ash saplings with different dieback severity

This section refers to the specific study done in two of the plots, Gremecey and Seichamps in 2014-15. Most of leaf symptoms that were observed in early September 2014 were partial leaf necrosis with one or several leaflets with necrosis but most leaflets still green. No shoot mortality was observed in September 2014, the mortality occurring later, during the winter. The likelihood of leaf necrosis was significantly higher in Gremecey than in Seichamps (likelihood Chi-square= 4.55, p= 0.033, 38.0 ± 6.5 % in Gremecey and 20.6 ± 4.4 % in Seichamps).The percentage of leaves with necrosis was similar on saplings showing either no or severe dieback (dieback score of 0-1 versus 3, likelihood Chi-square of 0.7084, p= 0.400, Fig 2a). This was observed in the two studied plots as the plot * dieback status interaction had no significant effect on leaf necrosis frequency (likelihood Chi-square = 1.32, p= 0.251).

H. fraxineus was able to produce apothecia on rachis of both the saplings with no and severe dieback at the same rate (crown status of 0-1 versus at least 3, likelihood Chi-square = 1.19, p= 0.276, Fig 2b). The saplings with no dieback experienced limited mortality of the marked shoot while the shoot mortality was high for saplings showing dieback (likelihood Chi-square = 6.99, p= 0.008, Fig 2c). In summer 2015, the crown status of the saplings had not changed, i.e. only very minor signs of *H. fraxineus* infection were observed on saplings with no dieback symptoms in 2014.

Relationship between leaf necrosis in fall and subsequent shoot mortality

Shoot mortality in spring was strongly related to the frequency of leaves with necrosis at the end of the previous summer (Fig 3a, b, Table 1). It is noteworthy that a small proportion of shoots that showed no leaf necrosis in September were nevertheless dead in the next spring (Fig 3a). Shoot mortality was very different on ashes with different dieback status (Table 1, Fig 3a). The odds ratio for shoot mortality of an ash with dieback versus one without was 4.1 (95% credible interval [2.4, 7.0]). By contrast, leaf necrosis did not significantly depend on tree dieback status (Table 1, mean leaf necrosis frequency of 12.9 ± 3.8 % for ashes without dieback

and 16.3 ± 6.3 % for ashes with dieback). Both leaf necrosis and shoot mortality were strongly affected by the tree random factor with a similar range of standard deviation (Table 1). This means that, even after other variables of the model are taken into account (% tree cover, dieback status), some of the rated ashes consistently showed more leaf necrosis or shoot mortality across years. This effect was of the same magnitude for leaf necrosis and for shoot mortality.

Shoot mortality was also significantly increased at higher tree cover (Table 1, Fig 3b), with an odds ratio of 1.2 (95% credible interval [1.0, 1.3]) per 10% tree cover. By contrast, leaf necrosis was not affected by tree cover (Table 1). The environment random factor induced a level of variability similar for leaf necrosis and shoot mortality (Table 1). As no meso-climate effect was included in this model, high environment random factors were expected.

Early defoliation was significantly associated with shoot mortality, with shoot mortality increasing with the defoliation level (Table 1). Defoliation appeared to be a consequence of infection by *H. fraxineus* and significantly increased with leaf necrosis likelihood (likelihood Chisq of 58.3, $p < 0.0001$).

Relationship between leaf necrosis or shoot mortality and climate

The preliminary analysis showed that the best fit for foliar necrosis occurred for climatic conditions in June although the difference was minor (DIC of 99.8 compared to DIC of 100.9 for 15 May to 15 June and 113.7 for 15 June to 15 July). Likewise, for shoot mortality, the best fit occurred for climatic conditions in September to end of December (DIC of 99.8 compared to DIC of 103.5 for climatic conditions in September-October).

The model fitted adequately the data, with a proper residual distribution and an acceptable prediction of observed values in the cross-validation (Fig S1). The main parameter influencing leaf necrosis was TX78, the average of daily maximal temperature in July and August with a strong negative effect of high summer temperatures (Table 2, Fig 4a, odds ratio of 0.70, 95% credible interval CI [0.63, 0.84]). High average temperature in June increased the likelihood of leaf necrosis (Table 2, odds ratio of 1.33, CI [1.11, 1.52]). By contrast, rainfall either in June or in July-August was not related to leaf necrosis likelihood. Lastly, we observed a trend of decreasing leaf necrosis likelihood with the time of ash dieback in the area (Table 2, Fig 4c, odds ratio of 0.88, CI [0.83, 0.93]).

The shoot mortality was mainly linked to the average temperature in autumn, with mortality likelihood decreasing with increasing autumn temperatures (September to December, Table 2, Fig 4d, odds ratio of 0.69, CI [0.54, 0.86]). Rainfall in autumn was not related to shoot mortality (Table 2). As in the shoot level model, shoot mortality increased significantly with leaf necrosis frequency (Table 2). As a consequence, all parameters influencing leaf necrosis likelihood also influenced shoot mortality (Fig 4 e, f and g).

Ability of *H. fraxineus* to produce apothecia on overwintered rachises in the fructification assay

The presence of *H. fraxineus* in rachises at leaf fall was tested in 2020 and 2021 in part of the sites by qPCR. It showed striking differences between years and locations (Table 3). Moreover, it was not significantly linked to both the leaf necrosis frequency measured in September, two months before (Pearson R= 0.208, pvalue= 0.440) and the subsequent shoot mortality (Pearson R= 0.266, pvalue= 0.320). In Champenoux, although the frequency of leaf necrosis was low both in 2019 and [illegible]20, the proportion of rachis in which *H. fraxineus* was detected by qPCR was very different between the two years (Table 3). Another striking feature

was that the frequency of *H. fraxineus* detection by qPCR in rachises at leaf fall was almost ten-fold higher than the frequency of necrotic leaves.

The proportion of rachises producing apothecia in the fructification assay in laboratory conditions showed very high variability, with a range of 0.01 to 0.99. This strongly depended on the year with mean values of 0.26 ± 0.11 in 2016, 0.52 ± 0.17 in 2017, 0.79 ± 0.09 in 2019 and 0.37 ± 0.23 in 2020. The proportion of rachises producing apothecia was significantly correlated to the proportion of rachises in which *H. fraxineus* was detected by qPCR at leaf fall (Pearson R= 0.710, pvalue= 0.002).

The proportion of rachises producing apothecia in the fructification assay was not significantly related to the frequency of leaf necrosis in the previous September rating (Fig 5a). It was not significantly related to the tested climatic parameters during the overwintering period (Table 4). By contrast, it was significantly related to the rainfall during the previous summer (sum of rainfall in July-August, Table 4, Fig 5b). It was also not related to the % of tree cover (Table 4).

Climate suitability in France for the different stage of *H. fraxineus* life cycle

Maps were produced using the developed models (Table 2 and 4) for an average site (site random factor fixed at 0) and for years 2010 to 2020. *H. fraxineus* should be able to colonise leaves, be present on rachises shed in autumn and be able to produce apothecia in spring in most of France (Fig 6a). By contrast, its ability to produce damage should be much more limited, in particular in southern France (Fig 6b, c and d). However, its ability to cause damage to ash trees is strongly dependent on local site conditions: comparison of Fig 6c and Fig 6d show large difference of prediction shoot mortality likelihood depending on the % tree cover. Uncertainty in parameter estimation very significantly affected both the maps of necrotic leaves / shoot mortality probability (Fig S2).

Discussion

We showed that carrier trees without crown dieback symptoms exist and play a significant role in ash dieback epidemiology (here after referred to as “healthy carriers”). *H. fraxineus* is able to establish on ash leaves and to reproduce on the leaf debris in the litter (rachises) in situations where it induces very limited shoot mortality. This can be on ash trees that show no dieback while their neighbors show some (Fig 2 and 3a), in specific local environment (low tree cover, Fig 3b) or meso-climates (Fig 6) Climate strongly influence *H. fraxineus* with different parameters being important depending on the life cycle stage (Fig 4, Fig 5). As a consequence, while the pathogen might be able to establish over most France, it should to induce far less severe dieback in large parts of southern France (Fig 6). This is likely to occur in others area of southern Europe. Last, we observed a decreasing trend of severity (leaf necrosis and shoot mortality likelihood) with the time of disease presence (Fig 4 DH).

We were able to predict the severity of dieback in spring by observing leaf symptoms in the previous autumn. Leaf necrosis frequency in September was a good predictor of subsequent shoot mortality (Fig 3). Despite that, leaf necrosis was a very poor predictor of rachises infection at leaf fall and of the ability of *H. fraxineus* to produce apothecia, and therefore inoculum, on the rachises in spring (Fig 5, Fig 7). This may be because of the two-month delay between leaf necrosis measurement and rachises colonisation assessment. It has to be pointed out that very little inoculum production is observed September and October in French conditions (Grosdidier *et al*, 2018). Nevertheless, lesions present in the leaflets might have extended in the rachises. Another likely explanation is that *H. fraxineus* has the ability to

asymptomatically infect ash leaves in early summer and to behave as a latent pathogen for an extended period (Cross *et al*, 2017). These authors showed that leaf necrosis occurs only when *H. fraxineus* inoculum reaches a certain level in the leaf which happens at the same time as leaf senescence and of a change in the leaf microbiota composition. It would appear that the high *H. fraxineus* levels in leaves required for leaf necrosis also strongly promote shoot infection from the petiole. By contrast, high *H. fraxineus* levels may not be required for rachis colonisation at leaf fall in senescent tissue. This would explain that high rachis colonisation may be observed in situations with limited previous leaf necrosis.

Because high rachis colonisation may occur while symptom frequency, leaf necrosis or shoot mortality, is low (Table 3), *H. fraxineus* is able to complete its biological cycle in conditions where no significant dieback is observed and some ashes behave as healthy carriers (Fig 7).

This is the case at local level, on specific trees that remain without shoot mortality despite experiencing leaf necrosis and enabling the pathogen to complete its cycle on infected rachises (Fig 2). We did not find difference in leaf necrosis frequency between saplings showing dieback or not (Table 1). The measure of leaf infectio[illegible]ight not have been optimal as we only rated the presence of necrosis on the leaf without any measure of necrosis severity such as number of affected leaflets. Also, we examined only branches within reach to assess the disease severity, which may not be representative of disease severity higher in the crown. Leaf infection might be higher close to soil level as spore load in the air was shown to decrease from 1 to 3 m above soil level (Chandelier *et al*, 2014) and because microclimate is more humid close to the soil and thus more favourable to *H. fraxineus* infection. Our experimental design did not allow to determine whether the ash trees that did not show dieback did so for genetic reasons or because they were in microsites unfavorable to the disease or harbored foliar microbial competitors preventing *H. fraxineus* development. Resistance toward *H. fraxineus* has been widely documented in *F. excelsior* (McKinney *et al*, 2011, Semizer-Cuming *et al*, 2021) and thus remains a very likely explanation for at least part of the observed pattern, although different micro-climatic conditions can be another one. It does not appear that some of the studied trees remained without dieback because they avoided inoculum as they showed a frequency of leaf necrosis similar to trees that showed dieback symptoms in the same plots. In the two stands studied in more detail in 2014, the ash density was high and the saplings remaining without dieback were in close proximity to dying saplings, questioning the possibility that they were experiencing different environmental conditions. Nevertheless, we do have evidence that environmental conditions strongly influence the ability of *H. fraxineus* to infect the shoot from the leaf petiole. Indeed, although the likelihood of leaf necrosis did not depend on the tree cover, the subsequent likelihood of shoot mortality was much lower in hedges with a low tree cover compared to forest plots with a high tree cover (Fig 3b). We also showed that the colonisation of rachises at leaf fall and subsequent ability of *H. fraxineus* to complete its cycle on the colonised rachises does not depend on the tree cover (Table 3), being similar in forests conditions and in hedges in open landscape. Hence, trees with no dieback symptoms in infected stands behave as heathy carriers, allowing *H. fraxineus* to reproduce on their leaves as efficiently as on the leaves of neighboring trees that experience dieback. They should even be much better inoculum producers than trees affected by dieback as they retain full foliage and thus produce abundant infected rachises. This result is in agreement with the observation of Grosdidier *et al* (2020) that the density of infected rachises was similar in the litter beneath ash trees in hedges and in forest settings although the trees showed far less dieback symptoms in the latter situation. A possible explanation is that as the tree cover does not affect inoculum production, it does not affect infection frequency. However, at low tree cover, crowns are exposed more frequently to micro-climatic conditions adverse to *H. fraxineus* (hotter and dryer

conditions, Grosdidier *et al*, 2020), preventing the increase of the pathogen present as endophyte in leaves and thus later shoot mortality. This discrepancy between the ability to reproduce on the host and the ability to induce significant damages has been reported for other tree pathogens. For example, *Diplodia sapinea* mainly produce inoculum on pine cones and is widely present on cones in healthy pine stands; it however needs weakening of the trees to induce significant damages (Palmer *et al*, 1988, Blodgett *et al*, 1997).

At a larger scale, we showed that *H. fraxineus* may be able to fulfil its cycle in regions were climate does not allow significant dieback (Fig 6). The disease has been progressing very regularly in France from 2009 to 2016 with a very noticeable slowdown of the spread in southern France from 2017 to 2019 (Fig 1). The slowdown of the spread occurred in regions with climate unfavorable to ash dieback. In 2020, the Pyrenean area was colonised while no sign of the disease had been reported north in the Garonne valley (data from the Département de la Santé des Forêts). It is possible that the pathogen was introduced via infected planted ashes (Wylder *et al*, 2018). However, our results show that the spread without dieback symptoms of *H. fraxineus* in the Garonne valley is also a likely hypothesis. This may have been enhanced by the very hot summers of 2018-20, which were even less favorable to *H. fraxineus*-induced shoot mortality. The delay of 3-4 years observed until the first report of ash dieback in the Pyrenean piedmont is in line with the spread speed observed in France of about 60 km per year (Grosdidier, 2017).

The decreasing trend of leaf necrosis and shoot mortality likelihood with years of disease presence may be very significant but requires confirmation. It may well be a spurious result caused by the very dry and hot climate experienced in northern and eastern France in 2018-20, at the end of the study period. Climatic conditions were taken into account in our model and summer high temperature was one of the most important feature to explain symptoms severity. But the parameter we used (average daily maximal temperature) may not have accounted to entire effect of heat waves observed during this 2018-20 period. Another possible explanation is the progressive selection of more tolerant ashes in sites affected by ash dieback since a long time. Indeed, in the site followed for the longest period, less than 50% of the seedlings selected in 2015 were still alive in 2021. We tried to replace dead saplings by symptomatic saplings to avoid a progressive increase in tolerant ash frequency in our sample, but this was not always possible.

Early leaf senescence was hypothesized to be a mechanism of tolerance to *H. fraxineus*, the pathogen then being shed in the leaves before getting the chance to infect shoots (Mc Kinney *et al*, 2011, Schwanda & Kirisits, 2016). We were not able to confirm the hypothesis as early defoliation was associated with higher shoot mortality in our data. However, care has to be taken when interpreting this result. Mc Kinney *et al* (2011) reported that some ash trees show early leaf senescence as a genetically controlled trait, and thus show early defoliation, limiting the transfer of *H. fraxineus* from leaves to shoots. In our experiment, early defoliation was a result of infection, being tightly associated with high leaf necrosis frequency. In these conditions, transfer of the pathogen from leaves to shoots may not be hampered. This thus likely represent a different mechanism.

Climate appears to be a major driver of symptoms induction by *H. fraxineus*. High summer temperatures was expected to limit disease severity, as the pathogen has been shown to have low survival at temperature above 35°C (Hauptman *et al*, 2013). Moreover, the hot summers of the Rhône valley south of Lyon limit ash dieback severity in South-East France (Grosdidier *et al*, 2018). More surprising is the low correlation of spring and summer rainfall with disease severity as it has been associated with high disease impact through increased inoculum production (Dvořák *et al*, 2016, Skovsgaard *et al*, 2017, Chumanová *et al*, 2019).

Spring and summer rainfall may promote infection of the leaves while *H. fraxineus* remains as an asymptomatic endophyte especially if high summer temperatures prevent the heavy colonisation of ash leaves by the pathogen. This could explain why spring and summer precipitations were not well correlated ~~to~~ frequency of symptoms such as leaf necrosis and shoot mortality (Table 2). By contrast, summer rainfall was strongly associated with both high rachis infection and ability to produce apothecia in the following spring. For example, in 2019, very limited leaf necrosis occurred in the Champenoux area because of high summer temperatures although summer rainfall allowed good colonisation by *H. fraxineus* of the rachises shed during the autumn (Table 3). This might be the likely mechanism by which spring and summer rainfall favour high ash dieback impact. The period in spring with the best fit between climatic conditions and leaf necrosis likelihood was in June, although the difference with other tested periods was low. This may appear surprising as the period of ascospores production has often been shown to be in summer, in July to August (Chandelier *et al*, 2014; Dvorak *et al*, 2016; Hietala *et al*, 2013; Timmermann *et al*, 2011). However, many of those results correspond to central or northern Europe; in North-East France, the period of ascospores production was earlier, from mid-June to mid-July (Grosdidier *et al*, 2018) and ascomata have been observed as early as the first week of May in the Pyrenean sites during this work. Shoot mortality was associated with previous leaf necrosis, but also ~~to~~ subsequent mild autumn temperature. This is the first report of an association of shoot mortality with autumn climatic conditions and the mechanism involved remains to be elucidated.

H. fraxineus ability to infect ash leaves and reproduce on the rachises in the forest litter does not appear to limit the pathogen ability to establish throughout France. By contrast, the low ability to induce shoot mortality explains the absence of ~~the disease report~~ in many areas of southern France. Hence, the maps showing the shoot mortality likelihood match well with the ash dieback risk predicted by Goberville *et al* (2016). The statistical niche model produced by these authors was based on the distribution in Europe of this invasive pathogen still spreading and with a presence still limited to North-Easter France; it nevertheless well captured the potential climatic niche in France. This would confirm that south-eastern France is not favorable to ash dieback, as was suggested by Grosdidier *et al* (2018).

Acknowledgement

We wish to thank Olivier Caël and Anaïs Gillet for technical assistance in handling the analysis of rachises ability to produce apothecia and level of infection by *H. fraxineus*. The establishment and rating of the plots in the Charente area was done by Dominique Piou while those of plots from Brittany was done by Laurence Roche from the Département de la Santé des Forêts. We also thank Mireia Gomez-Gallego for her advices in the analysis and for reviewing the manuscript and the two anonymous reviewers that helped improve the manuscript. This work was funded by the French Forest Health Department, the French Ministry in charge of Agriculture and Forestry and by the project HOMED from the European Union's Horizon 2020 Program for Research & Innovation under grant agreement No 771271. The UMR1136 research unit is supported by a grant managed by the French National Research Agency (ANR) as part of the "*Investissements d'Avenir*" program (ANR-11-LABX-0002-01, Laboratory of Excellence ARBRE).

Reference

Alizon S, Michalakis Y. Adaptive virulence evolution: the good old fitness-based approach. Trends Ecol. Evol. 2015; 30:248–54.

Blodgett JT, Kruger EL, Stanosz GR. *Sphaeropsis sapinea* and Water Stress in a Red Pine Plantation in Central Wisconsin. Phytopathology, 1997; 87:429–434.

Chandelier A, Helson M, Dvorak M, Gischer F. Detection and quantification of airborne inoculum of *Hymenoscyphus pseudoalbidus* using real-time PCR assays. Plant Pathology. 2014; 63:1296–1305.

Chumanová E, Romportl D, Havrdová L, Zahradník D, Pešková V, Černý K. Predicting ash dieback severity and environmental suitability for the disease in forest stands. Scandinavian Journal of Forest Research. 2019; 34:254–266.

Cross H, Sønstebø JH, Nagy NE, Timmermann V, Solheim H, Børja I, et al. Fungal diversity and seasonal succession in ash leaves infected by the invasive ascomycete *Hymenoscyphus fraxineus*. New Phytol. 2017 213:1405–1417.

Cunniffe NJ, Koskella B, E. Metcalf CJ, Parnell S, Gottwald TR, Gilligan CA. Thirteen challenges in modelling plant diseases. Epidemics 2015; 10:6–10.

Davidson JM, Wickland AC, Patterson HA, Falk KR, Rizzo DM. Transmission of *Phytophthora ramorum* in Mixed-Evergreen Forest in California. Phytopathology 2005; 95:587–596.

DiLeo MV, Bostock RM, Rizzo DM. *Phytophthora ramorum* Does Not Cause Physiologically Significant Systemic Injury to California Bay Laurel, Its Primary Reservoir Host. Phytopathology 2009; 99:1307–1311.

Dvorak M, Rotkova G, Botella L. Detection of Airborne Inoculum of *Hymenoscyphus fraxineus* and *H. albidus* during Seasonal Fluctuations Associated with Absence of Apothecia. Forests 2013; 7:1.

Figueroa-Zúñiga JI, Arellano-Valle RB, Ferrari SLP. Mixed beta regression: A Bayesian perspective. Computational Statistics & Data Analysis 2013; 61:137–147.

Flowers J, Nuckles E, Hartman J, Vaillancourt L (2001). Latent Infection of Austrian and Scots Pine Tissues by *Sphaeropsis sapinea*. Plant Disease 2001: 85:1107–1112.

Garbelotto M, Schmidt D, Swain S, Hayden K, Lione G. The ecology of infection between a transmissive and a dead-end host provides clues for the treatment of a plant disease. Ecosphere 2017; 8:e01815.

Goberville E, Hautekèete N-C, Kirby RR, Piquot Y, Luczak C, Beaugrand G. Climate change and the ash dieback crisis. Scientific Reports 2016; 6:35303.

Grosdidier M. Épidémiologie de la chalarose du frêne, une maladie causée par l'agent pathogène *Hymenoscyphus fraxineus* [dissertation]. Nancy : Université de Lorraine (PhD, in french) ; 2017.

Grosdidier M, Ioos R., Husson C, Cael O., Scordia T, Marçais B. Tracking the invasion: dispersal of *Hymenoscyphus fraxineus* airborne inoculum at different scales. FEMS Microbiology Ecology, 2018; 94:fiy04.

Grosdidier M, Ioos R, Marçais B. Do higher summer temperatures restrict the dissemination of *Hymenoscyphus fraxineus* in France? For. Path. 2018; e12426.

Grosdidier M, Scordia T, Ioos R, Marçais B (2020) Landscape epidemiology of ash dieback. J. Ecol. 2020; 108:1789–1799.

Gross A, Holdenrieder O, Pautasso M, Queloz V, Sieber TN. *Hymenoscyphus pseudoalbidus*, the causal agent of European ash dieback. Molecular Plant Pathology 2014; 15:5–21.

Hauptman T, Piškur B, de Groot M, Ogris N, Ferlan N, Jurk D. Temperature effect on *Chalara fraxinea* : heat treatment of saplings as a possible disease control method. For. Path. 2013; 43:360–370.

Hietala AM, Timmermann V, Børja I, Solheim H. The invasive ash dieback pathogen *Hymenoscyphus pseudoalbidus* exerts maximal infection pressure prior to the onset of host leaf senescence. Fungal Ecol. 2013; 6:302–308.

Holdenrieder O, Marco P, Weisberg PJ, Lonsdale D. Tree diseases and landscape processes: the challenge of landscape pathology. Trends in Ecology & Evolution 2004; 19: 446–452.

Ioos R, Kowalski T, Husson C, Holdenrieder O. Rapid in planta detection of *Chalara fraxinea* by a real-time PCR assay using a dual-labelled probe. Eur. J.Plant Path. 2009; 125:329–335.

Kelnarová I, Černý K, Zahradník D, Koukol O, Sieber T. Widespread latent infection of *Cryptostroma corticale* in asymptomatic *Acer pseudoplatanus* as a risk for urban plantations. For. Path. 2017; 47:e12344.

Kowalski T, Holdenrieder O. The teleomorph of *Chalara fraxinea*, the causal agent of ash dieback. For. Path. 2009; 39:304–308.

Landolt J, Gross A, Holdenrieder O, Pautasso M. Ash dieback due to *Hymenoscyphus fraxineus* : what can be learnt from evolutionary ecology? Plant Path. 2016; 65:1056–1070.

McKinney LV, Nielsen LR, Hansen JK, Kjær ED. Presence of natural genetic resistance in *Fraxinus excelsior* (*Oleraceae*) to *Chalara fraxinea* (*Ascomycota*): an emerging infectious disease. *Heredity* 2011; 106:788–797.

McKinney LV, Thomsen IM, Kjaer ED, Nielsen LR. Genetic resistance to *Hymenoscyphus pseudoalbidus* limits fungal growth and symptom occurrence in *Fraxinus excelsior*. For. Path. 2012; 42:69–74.

Marçais B, Kosawang C, Laubray S, Kjaer E, Kirisits T. (2022). Ash dieback. In: Asiebu F, Kovalchuk, editors. Forest Microbiology Volume 2: Forest Tree Health. Academic Press, 2022. p. 215-237.

Marsberg A, Kemler M, Jami F, Nagel JH, Postma- Smidt A, Naidoo S et al.. *Botryosphaeria dothidea* : a latent pathogen of global importance to woody plant health. Molecular Plant Pathology 2017; 18:477–488.

Orton ES, Clarke M, Brasier CM, Webber JF, Brown JKM. A versatile method for assessing pathogenicity of *Hymenoscyphus fraxineus* to ash foliage. For. Path. 2019; 49:e12484.

Palmer M, Mcroberts R, Nicholls T. Sources of Inoculum of *Sphaeropsis sapinea* in Forest Tree Nurseries. Phytopathology 1988; 78:831–835.

Pagán I, García-Arenal F. Tolerance of Plants to Pathogens: A Unifying View. Annual Review of Phytopathology 2020; 58:77–96.

Quintana Seguı P, Le Moigne P, Durand Y, Martin E, Habets F, Baillon M et al. Analysis of near-surface atmospheric variables: Validation of the SAFRAN analysis over France, J. Appl. Meteorol. Climatol. 2008; 47:92–107.

Schwanda K, Kirisits T. Pathogenicity of *Hymenoscyphus fraxineus* towards leaves of three European ash species: *Fraxinus excelsior*, *F. angustifolia* and *F. ornus*. Plant Pathology 2016; 65:1071–1083.

Semizer-Cuming D, Chybicki IJ, Finkeldey R, Kjær ED. Gene flow and reproductive success in ash (*Fraxinus excelsior* L.) in the face of ash dieback: restoration and conservation. Annals of Forest Science 2021; 78:14.

Sieber TN. Endophytic fungi in forest trees: are they mutualists? Fungal Biology Reviews 2007; 21:75–89.

Skovsgaard JP, Wilhelm GJ, Thomsen IM, Metzler B, Kirisits T, Havrdová L et al. Silvicultural strategies for Fraxinus excelsior in response to dieback caused by *Hymenoscyphus fraxineus*. Forestry 2017; 90:455–472.

Timmermann V, Børja I, Hietala AM, Kirisits T, Solheim H. Ash dieback: pathogen spread and diurnal patterns of ascospore dispersal, with special emphasis on Norway. *EPPO Bull*. 2011 41:14–20.

Wylder B, Biddle M, King K, Baden R, Webber J. Evidence from mortality dating of *Fraxinus excelsior* indicates ash dieback (*Hymenoscyphus fraxineus*) was active in England in 2004–2005. Forestry: An International Journal of Forest Research 2018; 91:434–443.

Supporting Information:

Table S1. Studied sites

Fig S1. Cross-validation for the model at the plot level relating climatic and site parameters to leak necrosis and shoot mortality likelihood. A. Leaf necrosis, B. shoot mortality

Fig S2. Quantile 0.05 (Q5) and 0.95 (Q95) for the estimated likelihood of leaf necrosis (A, B) and shoot mortality (C, D). The map were computed using the parameter 0.05 and 0.95 quantiles obtained from the Bayesian procedure fit. The shoot mortality is computed for a forest situation (tree cover of 100%).

Figures captions

Fig 1. Location of the studied ash stands (black dot) relative to the year of first ash dieback report.

Data from the Département de la Santé des Forêts. Am, Fréchencourt, Be, Sarrance, Ch1-6, Champenoux (6 plots), Co, Colombier, Fe, Ance-Féas, Gr, Gremeçey, La, Landivisiau Lu, Lupé, Ol1-2, Oloron-Sainte-Marie (2 plots), Po, Pont-de-Buis, Ro, Roche-sur-Grane, Sa, Salignac-sur-Charente Sc, Scaër, Se, Seichamps). The region border shapefile can be uploaded at https://geoservices.ign.fr/telechargement.

Fig 2. Impact and ability of *H. fraxineus* to complete its cycle on ash saplings with either no or severe dieback.

(A) % leaves with necrosis in autumn 2014 (likelihood Chi-square of 0.7084, p= 0.400). (B) % of rachis length with presence of *H. fraxineus* apothecia in April 2015 (likelihood Chi-square = 1.19, p= 0.276). (C) % of rated shoot with bark infection in summer 2015 (likelihood Chi-square = 6.99, p= 0.008).

Fig 3. Modelled and observed shoot mortality for ash trees of different level of dieback

(A) Site with different dieback status (with dieback, score 0-1 or without, score of 2-3). (B) Sites with different tree cover. Points represent the observed data, pooled by increasing 10% leaf necrosis frequency. For tree cover, blue dots represent sites with 9-25 tree cover while red dots represent sites with 75-100 % tree cover. Points representing less than 10 shoots are not represented.

Fig. 4. Modelled and observed leaf necrosis and shoot mortality depending on climatic parameters and sites conditions.

The curves represent the predicted response for a given parameter, all other parameters being fixed at the average value, except for the number of year of ash dieback presence that is set to 1. The orange area (grey in printed version) represent the 95% confidence interval. Dots represent oberved values.

Fig. 5. Ability of *H. fraxineus* to produce apothecia on the ash rachises overwintered in common garden (fructification assay).

(A) Relation with leaf necrosis observed in the same plot two months before (pvalue= 0.304). (B) Relation with climate (pvalue= 0.006, Table 4).

Fig. 6. Map of climate suitability for *H. fraxineus* ability to produce apothecia on the ash rachises or symptoms on leaf and shoot of ash trees.

Safran meteorological data, 2010-20. (A). Proportion of ash rachises collected in fall producing apothecia in laboratory conditions after overwintering in a common garden (fructification assay). (B) Leaf necrosis likelihood. (C) Shoot mortality likelihood in open canopy (20% tree cover). (D) Shoot mortality likelihood in forest conditions (100% tree cover).

Fig.7. Hypothesised impact of studied environmental parameters on *H. fraxineus* life cycle.

Higher summer rainfall and higher spring temperatures are associated with higher rates of leaf necrosis leading to higher shot necrosis especially in high tree cover. Higher summer temperatures are associated with low leaf necrosis frequency at the end of the summer, but not to low colonisation of rachises at leaf-fall during autumn. Low autumn temperatures are associated with high shoot mortality (better transfer from leaves to shoot or lesion development in shoots?). The part of the cycle needed for inoculum production is indicated in blue while red arrows and text refers to symptoms development that does not lead to inoculum production. Cycle adapted from Marçais *et al* (2022).

Table 1. Relationship between leaf necrosis and shoot mortality at the shoot level. Parameters of the hierarchical Bayesian model

Variable	Leaf necrosis [95% credible interval]	Shoot mortality [95% credible interval]
Sapling dieback status (DS)	0.27 [-0.01, 0.55]	**1.27 [0.82, 1.75]**
Site tree cover (TC)	-0.01 [-1.14, 1.16]	**1.40 [0.07, 2.23]**
Defoliation at September rating (%,DEF)	-	**1.53 [0.38, 2.68]**
Probability of necrotic leaves (pl)	-	**1.75 [0.24, 3.27]**
Tree random factor (sd, Tree.l and Tree.s)	0.93 [0.82, 1.06]	0.89 [0.55, 1.24]
Site*year random factor (sd, Env.l and Env.s)	0.95 [0.65, 1.32]	1.22 [0.97, 1.54]
Deviance	4615 [4532, 4698]	

NOTE. Parameter with a 95% credible interval that does not contain 0 are considered significant and are marked in bold. Positive values indicate an association between high levels of both the symptom and of the environmental variable.

Table 2. Relationship between leaf necrosis or shoot mortality and climate parameter (plot level). Parameters of the hierarchical Bayesian model

Variable	Symptom	Parameter [95% credible interval]
Rain June (RA6, α_2)	Leaf necrosis	0.04 [-0.16, 0.25]
Temperature June (TM6, α_3)	Leaf necrosis	**0.69 [0.37, 0.99]**
Rain Junly-August (RA78 (α_4)	Leaf necrosis	-0.20 [-0.52, 0.11]
Average daily maximal temperature July-August (TX78, α5)	Leaf necrosis	**-1.11 [-1.50, -0.69]**
N year ash dieback presence (LDP, α6)	Leaf necrosis	**-0.37 [-0.58, -0.17]**
Site random factor (Site.l, sd)	Leaf necrosis	0.21 [0.01, 0.49]
Rain September-December (RA912, β_2)	Shoot mortality	-0.06 [-0.43, 0.29]
Temperature September-December (TM912, β_3)	Shoot mortality	**-0.83 [-1.30, -0.39]**
Site tree cover (TC, β_4)	Shoot mortality	**0.77 [0.07, 1.52]**
Probability of necrotic leaves (pl, β_5)	Shoot mortality	**4.55 [4.38, 8.12]**
Site random factor (Site.s, sd)	Shoot mortality	1.21 [0.68, 2.01]
Deviance	3745 [3667, 3826]	

NOTE. Parameter with a 95% credible interval that does not contain 0 are considered significant and are marked in bold. The variables are standardized to have zero mean and one standard deviation with the exception of pl, the leaf necrosis likelihood. Positive values indicate an association between high levels of both the symptom and of the environmental variable.

Table 3. Colonisation of rachises at leaf fall in November

	Year	Frequency of necrotic leaves in September	Frequency of *H. fraxineu*s detection on rachises in November (qPCR)
Champenoux	2019	0.09 ± 0.03	0.87 ± 0.19
	2020	0.05 ± 0.02	0.35 ± 0.12
Pyrenean piedmont	2020	0.12 ± 0.05	0.60 ± 0.18

Table 4. Influence of climatic parameters on the ability of *H. fraxineus* to produce apothecia in the fructification assay

Variable	Parameter	P~~value~~
Leaf necrosis frequency	-0.44	0.297
Rain in July-August (RA78)	1.23	<0.001
Mean maximal daily temperature in July-August (TX78)	-0.29	0.353
Rain in October-March (RA10-3 ow)	0.21	0.478
Mean temperature in October-March (TM10-3 ow)	0.55	0.089
Site tree cover (%, TC)	0.11	0.549
Site random factor (sd)	0.0003	-

Note: RA10-3 ow and TM10-3 ow, sum of rain and mean of daily temperatures at the overwintering sites from October to March.

Figure

Click here to access/download;Figure;Fig1.tiff

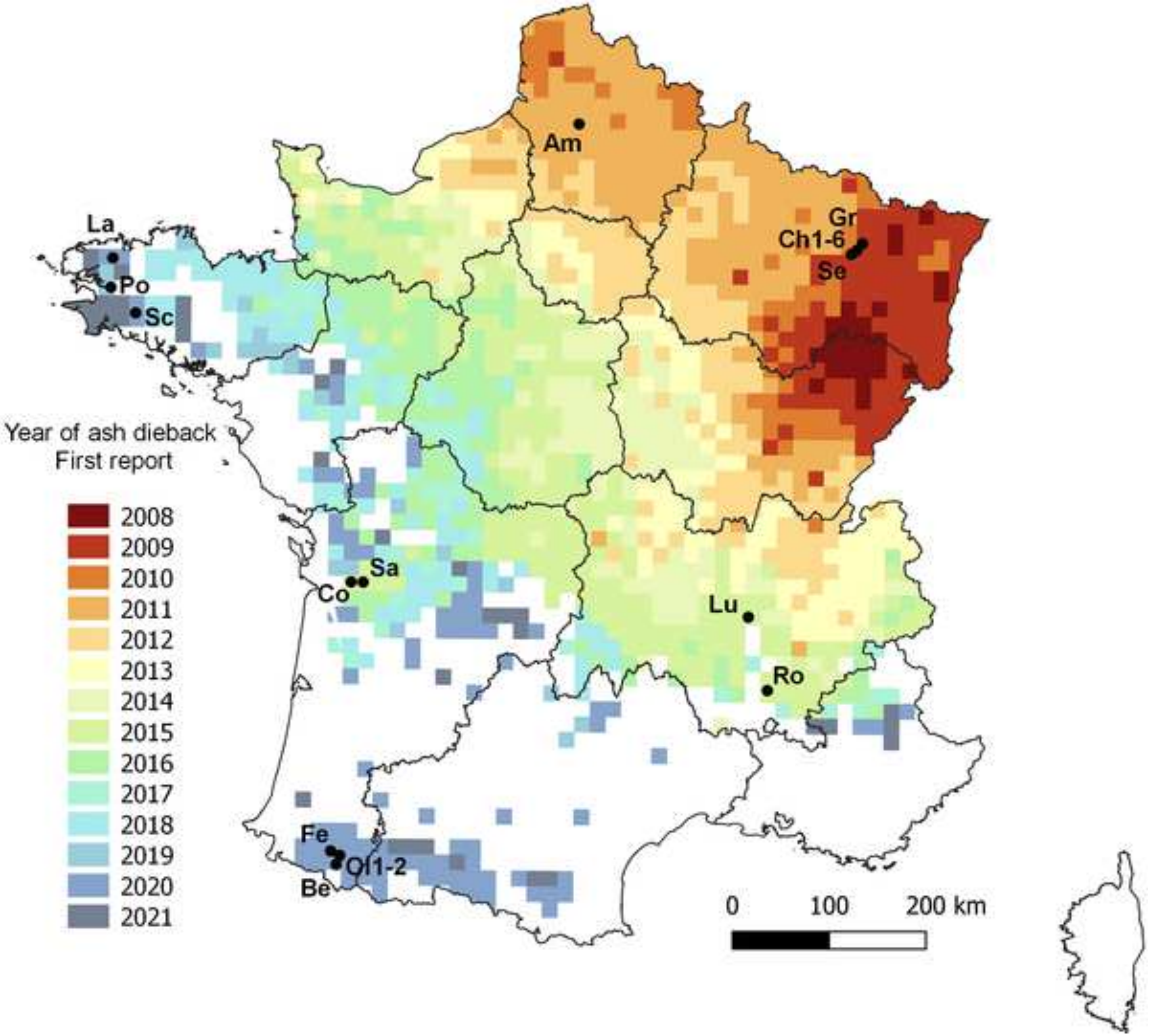

Figure

Click here to access/download;Figure;Fig2.tiff

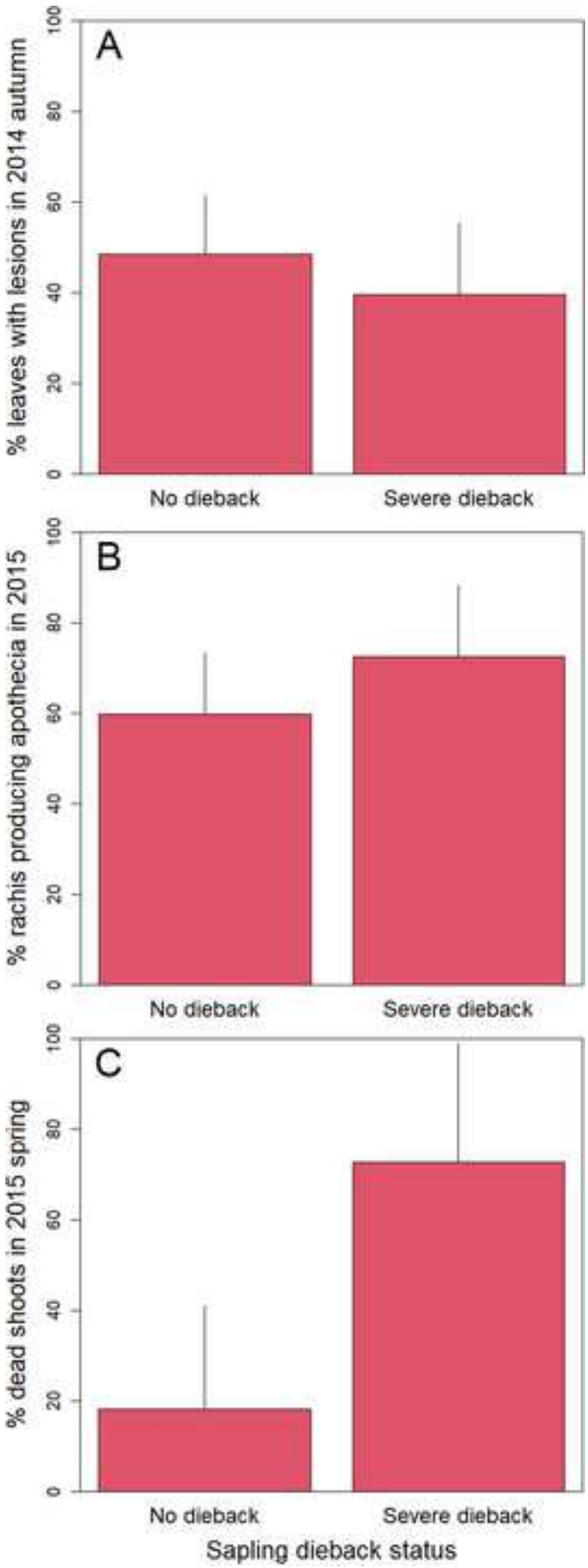

Figure

Click here to access/download;Figure;Fig3.tiff

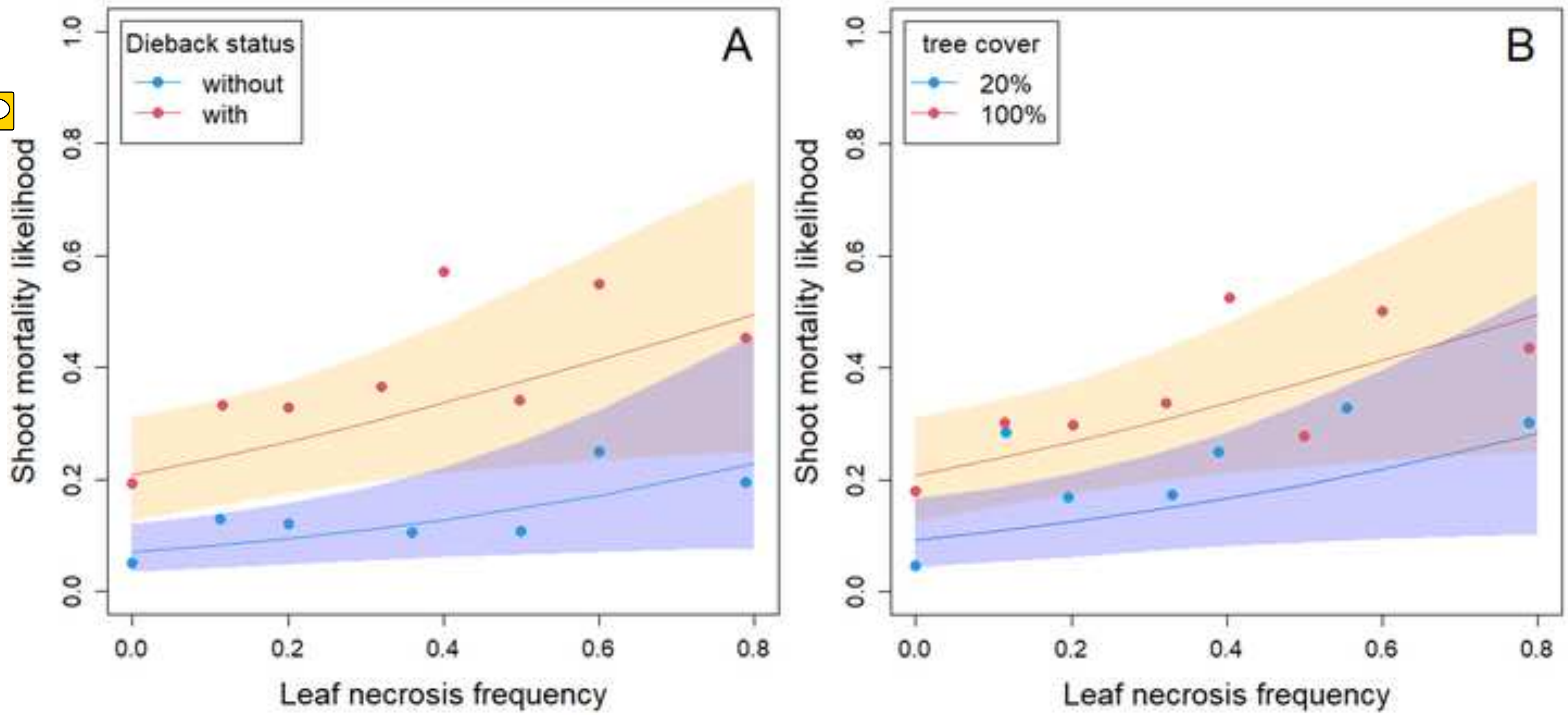

Figure Click here to access/download;Figure;Fig4.tiff

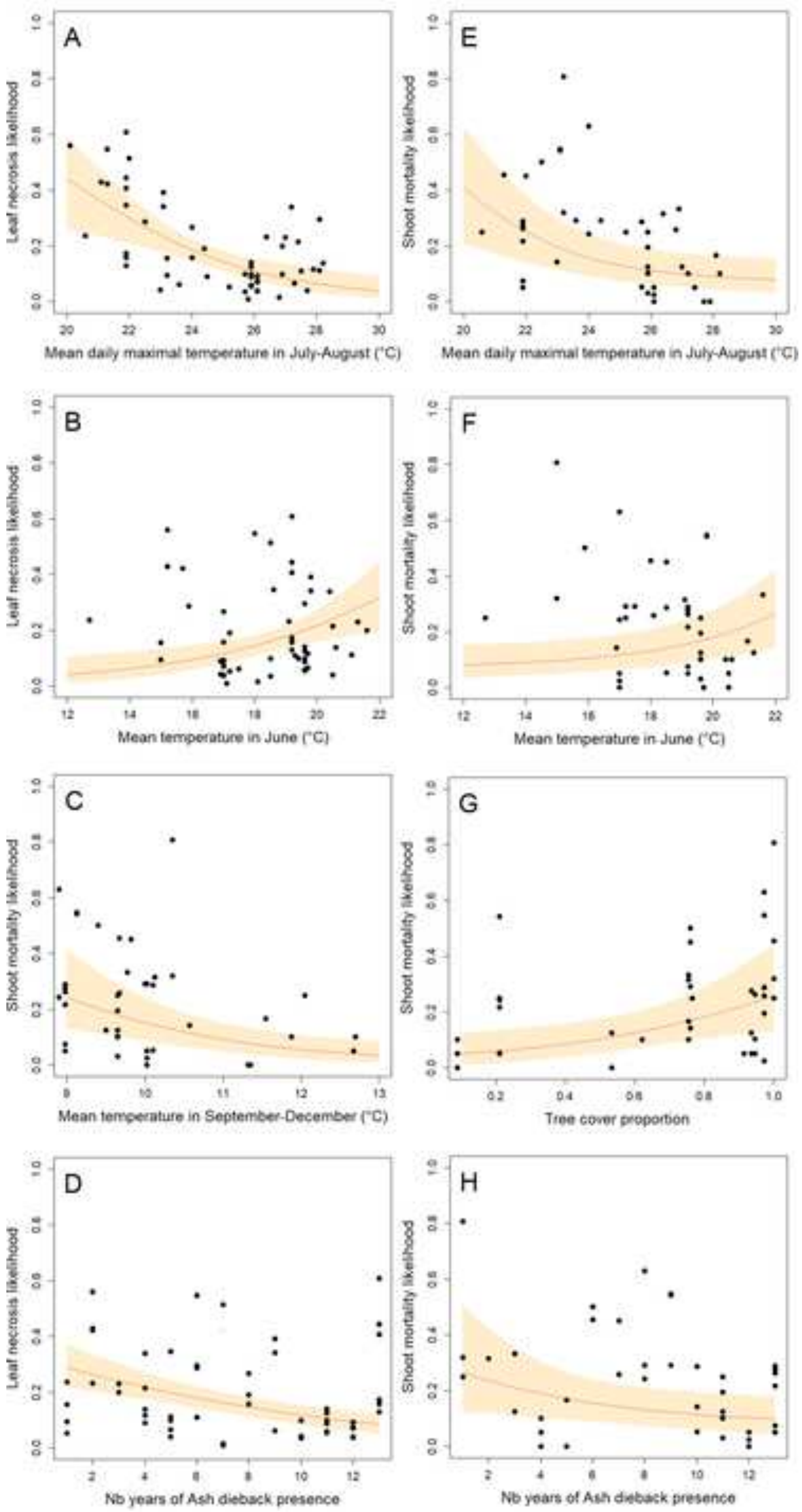

Click here to access/download;Figure;Fig5.tiff

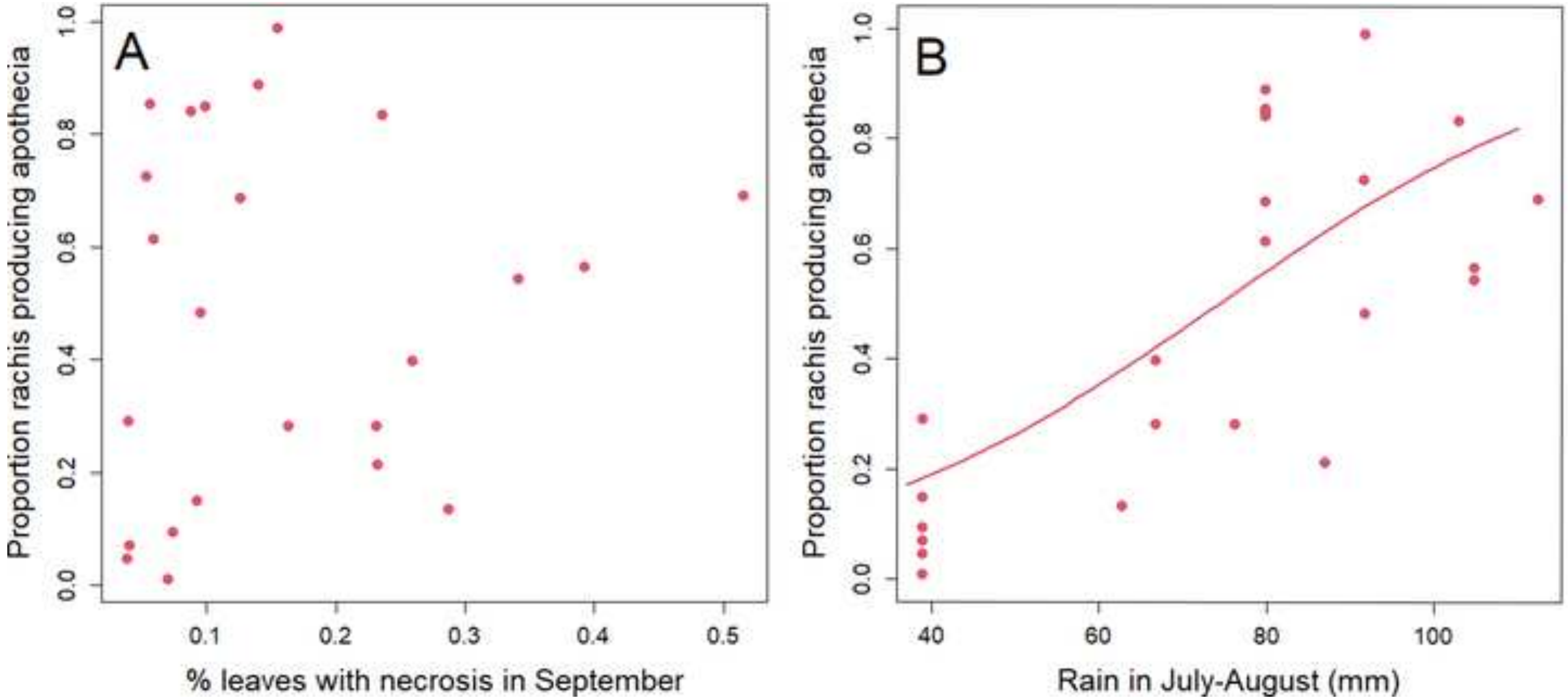

 Click here to access/download;Figure;Fig6.tiff

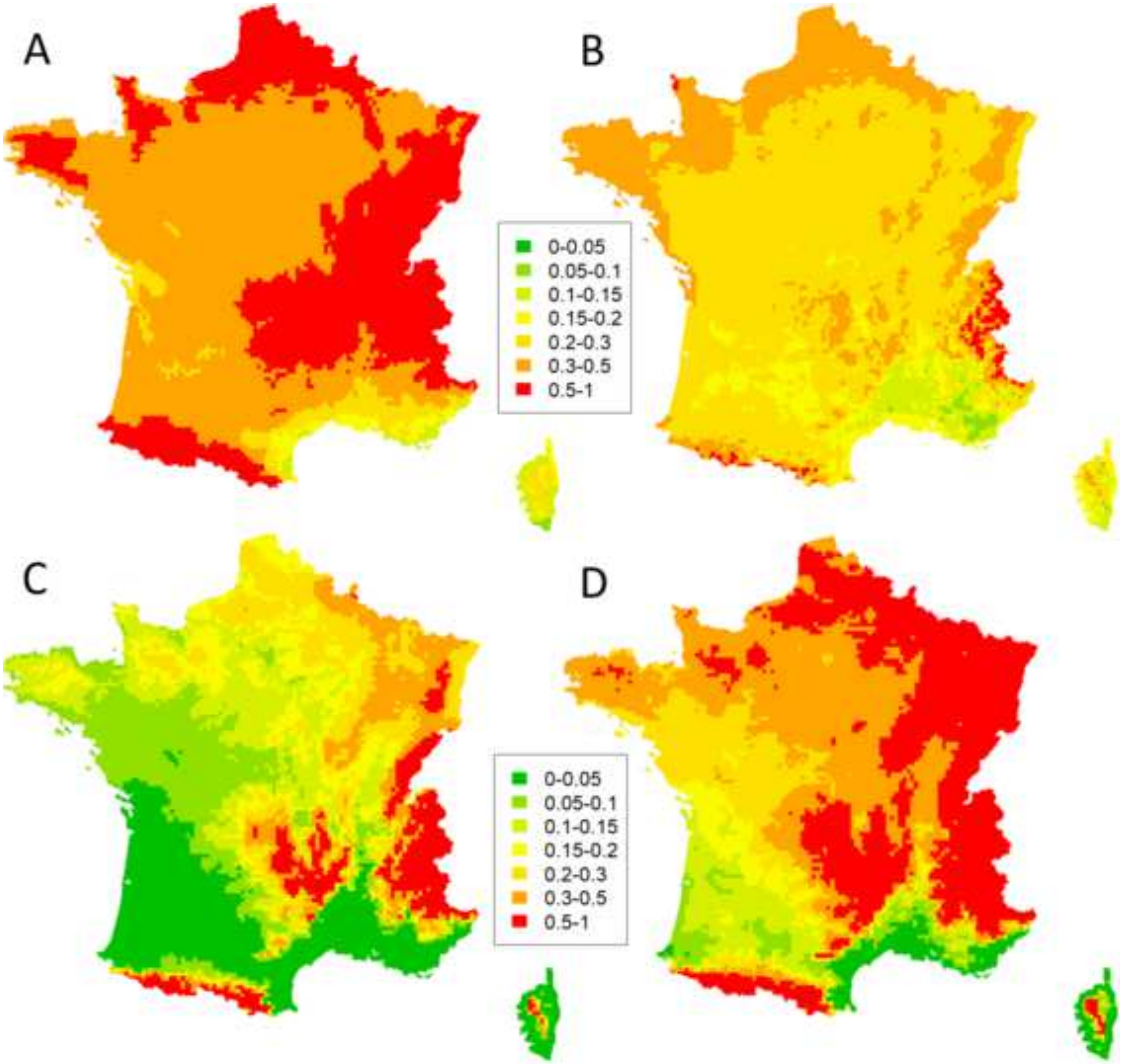

Figure
Click here to access/download;Figure;Fig7.tiff

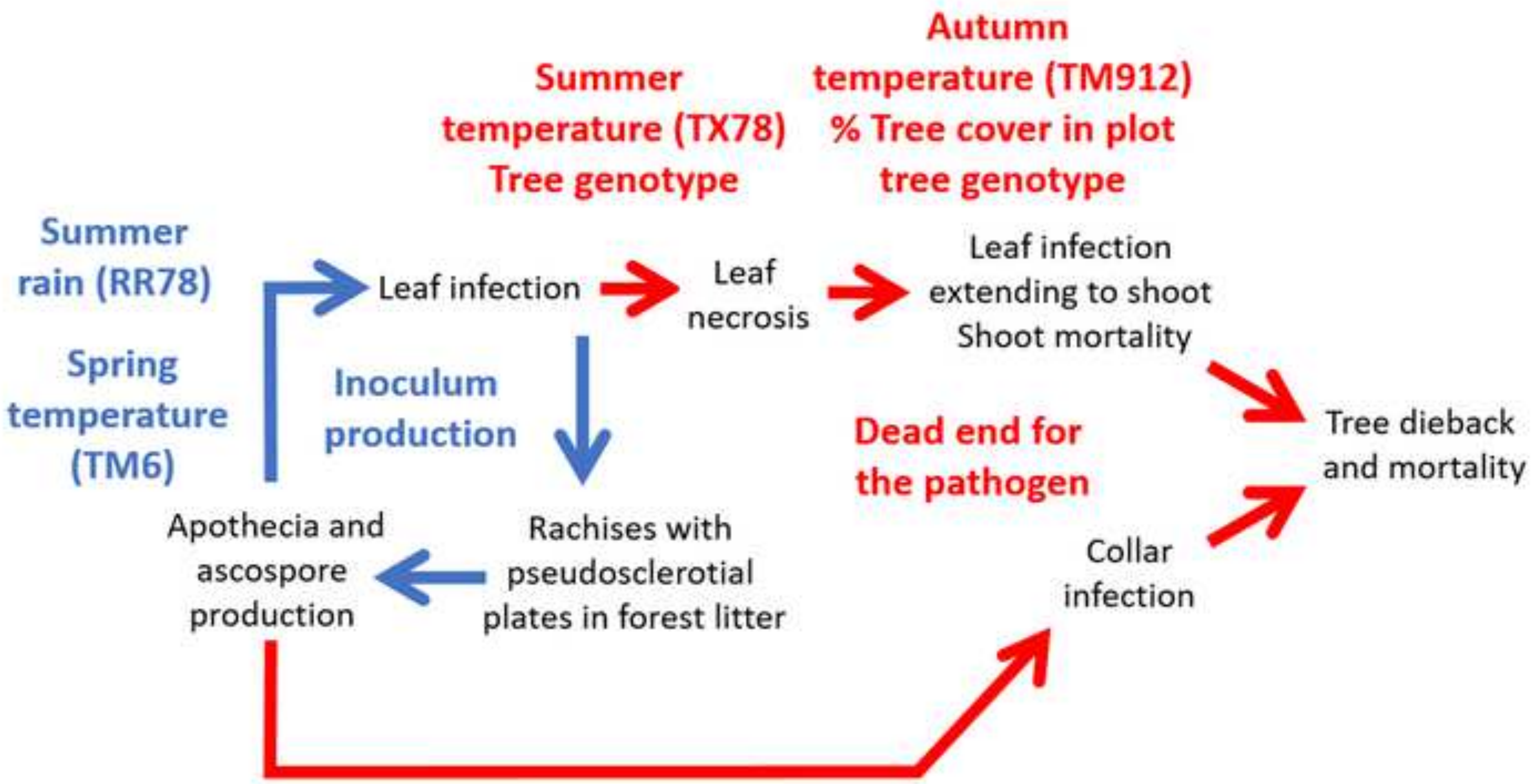

Click here to access/download
Supporting Information
Supplemental_data.docx

Ability of the ash dieback pathogen to reproduce and to induce damage on its host are controlled by different environmental parameters

Benoit Marçais[1*], Arnaud Giraudel[1], Claude Husson[2]

[1] Université de Lorraine, INRAE-Grand-Est, UMR1136 Interactions Arbres/Microorganismes, F-54000 Nancy, France

[2] Département de la Santé des Forêts, Ministère de l'Agriculture et de la Souveraineté Alimentaire - DGAL , F-75015 Paris, France

* Corresponding author

E-mail: benoit.marcais@inrae.fr

Abstract

Ash dieback, induced by an invasive ascomycete, *Hymenoscyphus fraxineus*, has emerged in the late 1990s as a severe disease threatening ash populations in Europe. Future prospects for ash are improved by the existence of individuals with natural genetic resistance or tolerance to the disease and by limited disease impact in many environmental conditions where ashes are frequent. Nevertheless, it was suggested that, even in those conditions, ash trees are infected and enable pathogen transmission. We studied the influence of climate and local environment on the ability of *H. fraxineus* to infect, be transmitted and cause damage on its host. We showed that healthy carrier, i.e. individuals showing no dieback but carrying *H. fraxineus*, exist~~s~~ and may play a significant role in ash dieback epidemiology. Environment strongly influenced *H. fraxineus* with different parameters being important depending on the life cycle stage. The ability of *H. fraxineus* to establish on ash leaves and to reproduce on the leaf debris in the litter (rachises) mainly depended on total precipitations in July-August and was not influenced by local tree cover. By contrast, damages to the host, and in particular shoot mortality was significantly reduced by high summer temperature in July-August and by high autumn average temperature. As a consequence, in many situations, ash trees are infected and enable *H. fraxineus* transmission while showing limited or even no damages. We also observed a decreasing trend of severity (leaf necrosis and shoot mortality likelihood) with the time of disease presence in a plot that could be significant for the future of ash dieback.

Author Summary

Ash dieback is an important tree disease induced by *Hymenoscyphus fraxineus* an invasive fungus. We studied the influence of climate and local environment on the ability of *H. fraxineus* to infect, be transmitted and cause damage on its host. We showed that healthy carriers, i.e. individuals showing no dieback but carrying *H. fraxineus*, exist~~s~~ and may play a significant role in ash dieback epidemiology. While the ability of *H. fraxineus* to establish and to reproduce on leaves mainly depended on summer rainfall, the damages it causes to the host depend on other parameters (high summer temperature in July-August, tree cover). As a consequence, in many situations, ash trees are infected and enable *H. fraxineus* transmission while showing limited or even no damages. This is in particular the case of non-forest situations such as hedges and isolated trees. ~~We also observed a decreasing trend in disease severity with the time of disease presence in a plot that could be significant for the future of Ash dieback.~~

Keywords: *Hymenoscyphus fraxineus*, climate, healthy carrier, latent infection, apothecia production

Short title: Environmental~~Climate~~ influence on ash dieback

Introduction

The existence of healthy carriers, i.e. infected individual that remain asymptomatic, is not recognized as an important feature in plant epidemiology (Cunniffe *et al*, 2015). The existence of healthy carriers has especially been reported for weak pathogens or those that can induce damage only on stressed hosts (so-called latent pathogens, Sieber, 2007). This is known to be a significant feature for important stress-induced tree diseases such as *Diplodia* shoot blight, sooty bark disease of maples or *Botryosphaeria* cankers (Flowers *et al*, 2001, Marsberg *et al*, 2017, Kelnarová *et al*, 2017). However, high production of inoculum usually occurs only at symptoms onset. By contrast, the existence of healthy carriers is seldom reported for primary pathogens that do not need host stress to induce significant host damages. Tolerance has been reported as a mechanism used by plants to cope with infection (Pagán & García-Arenal, 2020). This mechanism postulates that some individuals will limit the impact of infection on their fitness while sustaining significant pathogen load and enabling pathogen multiplication. However, the presence of individual plants that provide important levels of inoculum while exhibiting limited or even no visible symptoms is seldom reported. Disease severity is usually assumed to correlate positively with the multiplication of the pathogen within the host (Alizon & Michalakis, 2015). Nevertheless, at a different level, when comparing different species, it has been shown that poor correlation may exist between vulnerability (reduction of fitness caused by the infection) and competence (ability to sustain the epidemic by providing large amounts of inoculum). A striking example is given by Sudden Oak Death that developed in California in the last decades: while this disease, induced by *Phytophthora ramorum*, affects primarily *Fagaceae*, killing *Quercus* and *Lithocarpus* species, the main inoculum producer is a laurel, *Umbellularia californica* which coexist in natural forest and does not appear to suffer much fitness reduction (so-called reservoir host, Davidson *et al*, 2005, DiLeo *et al*, 2009, Garbelotto *et al*, 2017).

Such a discrepancy between pathogen's ability to establish in a stand and to produce inoculum and its ability to induce damages is an important difficulty for assessing the climatic niche of a pathogen. This assessment is a critical step to evaluate the risk associated with pathogen occurrence (Holdenrieder *et al*, 2004). The most widespread approach to develop species distribution models (SDM) is to relate the species known occurrence to environmental parameters, in particular climate, through statistical analyses (Elith & Leathwick, 2009). If large discrepancies exist between a pathogen presence and its ability to cause serious damage, this will lead to important under-reporting and to poor available data. By contrast, mechanistic SDM, which relies on the assessment of environment influence on the different stages of the pathogen cycle (Dormann *et al*, 2012) could be a suitable alternative to assess the climatic niche of a pathogen in such conditions.

Hymenoscyphus fraxineus is an invasive pathogen that has caused ash dieback throughout Europe since the late nineties and has jeopardized *Fraxinus excelsior* stands (Gross *et al*, 2014). *H. fraxineus* fulfils its life cycle on ash leaves. Those are infected by ascospores during late spring and summer and remain asymptomatic until late August ~~where~~ when necrotic leaf lesions develop (Cross *et al*, 2017). *H. fraxineus* then colonises the leaf petiole and may infect young shoots, inducing dieback (Gross *et al*, 2014). Most of the inoculum production occurs on leaf debris in the forest litter (so-called rachis that encompasses the petiole and the main vein of the composite leave, on which fruit bodies i.e. apothecia develop, Gross *et al*, 2014). By contrast, very limited inoculum production occurs on bark of diseased shoots which is considered to represent a dead-end for *H. fraxineus* (Landolt *et al*, 2016). It has been suggested that, in some conditions, *H. fraxineus* may reproduce on ash leaves without inducing significant dieback. Some *F. excelsior* individuals are not affected by *H. fraxineus*, even under high inoculum

pressure (McKinney *et al*, 2011). These individuals do not sustain extensive lesions when artificially inoculated at the shoot level (McKinney *et al*, 2012). It was then suggested that their fallen leaves could nevertheless sustain the pathogen reproduction and that the mechanism involved in containing the disease might be tolerance instead of resistance (Landolt *et al*, 2016). However, no experimental data is yet available to sustain this claim. Another reported tolerance mechanism would be early abscission that could prevent transfer of the pathogen from infected leaves to shoots but still allow sexual reproduction on fallen rachises (Mc Kinney *et al*, 2011, Schwanda & Kirisits, 2016, Orton *et al*, 2019). Also, environmental conditions may influence differently dieback and colonisation of the rachises in the litter. Grosdidier *et al* (2020) showed that in open canopy conditions, the infection levels of ash rachis by *H. fraxineus* in the litter was similar compared to forest stands with a close canopy despite much lower levels of crown dieback. Those data would suggest that healthy carriers could exist in the *F. excelsior* population, with individuals able to be infected in specific conditions at the leaf level and thus ~~allow~~ able to produce inoculum while sustaining very limited to no dieback.

We thus examined whether ash trees could in some conditions behave as healthy carriers. More specifically, we tested if different environmental variables may favour important stages~~we studied environmental influence on important stages~~ of ash dieback, namely the ability to colonise leaf rachises and reproduce on them and the ability to induce damage on ash trees, either leaf necrosis at the end of the summer or subsequently shoots mortality. We propose that ash behaving as healthy carriers could occur in locations where the environment promotes infection and reproduction of *H. fraxineus* but not symptom development.~~The aim was to provide basis for a latter development of a mechanistic SDM by documenting the key steps of *H. fraxineus* life cycle~~.

Material and methods

Sampled plots

We selected 20 plots on a temperature gradient in France (Fig 1, Table S1) in order to have a range of summer temperature as large as possible within the range *of H. fraxineus* presence. The plots were usually studied several years in a row starting from 2013 in the area ~~affected by~~where ash dieback was first observed in~~since~~ 2009-10 (NE France) to 2020 in an area just recently affected by ash dieback (SW and NW France, in Brittany and Pyrenean Piedmont). The studied plots were either in forest settings or located in hedges. The ash species present was *Fraxinus excelsior* in all plots. A summary of observations of leaf necrosis and shoot mortality on the plots is given in table S1. Plots were characterized by their tree cover from aerial photographs (Orthophoto with 50cm pixels from IGN, http://professionnels.ign.fr). The tree cover polygon within a radius of 100 m from each plot, including all tree species, were extracted and the proportion of tree cover within that range (TC) was computed.

Survey procedure

In all studied plots, 20 ashes were marked. In each plot, we selected suitable saplings that were contiguous, marking all those with two shoots available for rating. We usually selected saplings 2-4m high, although occasionally, larger trees with low-lying branches were selected. Preliminary analysis showed that the tree height did not influence leaf necrosis and shoot mortality likelihood. Saplings of different health conditions were usually present in the plots, with a proportion of saplings without dieback ~~symptoms~~ varying from 20 to 75% mostly depending on the length of ash dieback presence in the area.~~;~~ T~~t~~his proportion was adequate for the hypothesis we wanted to test. Dieback only refers to the mortality of perennial organs, here

the shoots; symptoms occurring on the leaves are not included. The saplings were rated annually for their crown status according to five dieback scores: 0 for complete absence of dead twigs, 1 for less than 10% of dead twigs, 2 for 10 to 50% of dead twigs, 3 for more than 50 % of dead twigs and 4 for dead saplings ~~dead~~ (Grosdidier *et al*, 2020). Two annual shoots per individual were marked for rating of ~~the~~ leaf ~~/~~ and shoot infection ~~in autumn~~. We selected the terminal shoot of the 2 lower branches that could be reached by an observer from the ground. As most sapling that were 2-4m high, this should not introduce any bias. Each sapling was rated~~Saplings were rated~~ for several years in row (1 to 6 years depending on the sites and on the year of first ash dieback report, Table S1). Dead twigs were replaced by the nearest shoot available from the lower part of the sapling. Dead saplings were replaced by the closest appropriate sapling, taking care to select saplings with ash dieback symptoms to avoid ending with only individuals potentially tolerant or resistant to *H. fraxineus*. On average, about 18% of the saplings were replaced over the study period, mostly saplings showing dieback as they suffered high mortality.

The necrosis induced by *H. fraxineus* on leaves was rated in September. The total number of leaves present on each marked annual shoot, the number of leaves with lesions (not taking into account the number of affected leaflet) and the number of leaf scars indicating defoliation were determined (altogether 4 to 27 leaves depending on the shoot). Lesions induced by *H. fraxineus* can be recognized as brown lesions starting at leaf margin and extending preferentially on leaf veins. Leaves presenting brown lesions only on the rachis were also rated as infected. Two leaves rated as presenting *H. fraxineus*-induced lesions were sampled for further analysis in the laboratory in 2018-20. Sampled leaves were stored individually in paper bags and kept at ambient temperature. In the laboratory, they were surface sterilized (5 min in 4% NaOCl and 1 min in 90% ethanol) and analysed by qPCR for the presence of *H. fraxineus* as described by Ioos et al. (2009). In the following year's vegetation season, the status of marked shoots was assessed as living or dead. Whenever the shoot had wilted because of girdling occurring below the annual shoot, we considered that it was not possible to determine whether infection had transferred from the annual shoot leaves to the stem and the data was counted as missing. ~~Saplings were rated for several year in row (1 to 6 years depending on the sites and on year of first ash dieback report, Table S1). Dead twigs were replaced by the nearest shoot available from the lower part of the sapling. Dead saplings were replaced taking care to select saplings with ash dieback symptoms to avoid ending with only individuals potentially tolerant to *H. fraxineus*.~~

In October-November, 20 rachises that had just fallen to the ground were sampled by site in order to assess the ability of *H. fraxineus* to produced apothecia on them (called hereafter fructification assay). The rachises were sampled at the base of the marked saplings (one rachis per sapling). This was done in all sites of Champenoux (2016, 2017, 2019, 2020), Fréchencourt (2016, 2017), Lupé (2016), Roche-sur-Grane (2017) and in the 4 Pyrenean sites (Ol1, Ol2, Fe, Be, 2020). The rachises were placed in mesh bags and overwintered in a common garden in a forest site in Champenoux (6.34251, 48.75297, EPSG 4326) except for the rachises of the plots close to Pau that were overwintered close to the site of Sarrance (-0.59692, 43.00044, EPSG 4326). This was done to minimize travel; ~~as we were mainly interested in the~~ climatic conditions during ~~rachis colonisation phase in June - October~~the vegetation season conditions determine inoculum production and leaf infection and we supposed that it would be the main factors controlling colonisation of the rachises. In the following spring, in April or May depending on the year, the rachises were retrieved from the mesh bags and were placed for 6-8 weeks at 18-20°C in humid chambers in large plastics boxes in contact with moistened filter paper to assess their ability to produce apothecia. The moistened filter paper was kept wet throughout the incubation and was changed weekly. Whenever a rachis produced *H. fraxineus* apothecia, the

rachis part covered with pseudosclerotial plate was removed and stored in paper bags for further analysis. The part without pseudosclerotial plate was left in the humid chamber. *H. fraxineus* apothecia can be recognized as 2-4 mm white apothecia with a dark base of the pedicels and smooth margin of the fruiting disk (Kowalski & Holdenrieder, 2009). The monitoring was stopped whenever no new production of apothecia was observed in a week (after 6-8 weeks incubation). Remaining rachises were then placed in ~~a~~ separate bags. The amount of rachises parts producing / not producing apothecia was determined by measuring the rachis length in 2016 and 2017 and by measuring their weight after drying 3d at 50°C in 2019 and 2020. The proportion of rachises producing apothecia was then computed.

In 2019 and 2020 in sites ~~of~~ in Champenoux and in 2020 in Pyrenean sites, 10 additional rachises were collected per site at the same time at leaf fall to determine the frequency of *H. fraxineus* by qPCR. Individual rachises were kept separate. They were dried 3d at 50°C, cut in small 1-2 mm sections and grinded with a mortar. A subsample of 50 mg per rachis was then used for ~~a~~ DNA extraction with DNAeasy Plant Mini kit (Qiagen). AP1 lysis buffer (800 µl), 4 µl RNase, two 3-mm and twenty 2-mm glass beads were added to the tube and the sample was grounded twice using a beadbeater (Tissue Lyser, Qiagen) at 30 Hz for 30 s. The DNA extraction was then conducted according to the manufacturer's recommendations. Total DNA was eluted in 150 µl AE buffer. The samples were then analysed by qPCR for the presence of *H. fraxineus* as described in Ioos et al. (2009).

Ability of *H. fraxineus* to complete its cycle on ash saplings with different dieback severity
~~**Ability of *H. fraxineus* to complete its cycle on ash saplings showing either severe or no dieback symptoms**~~

The ability of *H. fraxineus* to complete its cycle on ash saplings with no dieback symptoms was studied in two of the plots, Gremecey and Seichamps. Ash saplings about 2-3 m high were selected during summer 2014 in order to have 10-13 saplings of two types in each stand: i. saplings with either no or only very few scattered small dead shoots (no dieback, crown status of 0 or 1), ii. saplings strongly affected, with top shoots killed by *H. fraxineus* (crown status of at least 3). The two stands were densely stocked naturally regenerated ash stands heavily and uniformly infected by *H. fraxineus* since at least 4 years. The saplings without dieback were in close vicinity to individuals with severe dieback, with usually several severely affected saplings within less than one meter from the healthy-looking sapling.

On each sapling, an annual shoot with 7-28 leaves was selected and marked at the end of July. The selected shoot infection by *H. fraxineus* was rated at the beginning of September 2014 with record of the number of leaves with necrosis and total number of leaves. To measure the ability of *H. fraxineus* to complete its cycle on rachises, we placed a net around the entire crown of each sapling in mid-September to collect all the leaves falling during the autumn. The nets containing the leaves were retrieved at the end of October and placed on the ground outdoors in a nursery close to both plots to overwinter. Unfortunately, logging occurred in the Seichamps plot during ~~the~~ autumn and an engine destroyed many studied saplings. Only four saplings without dieback and one with severe dieback were available in that plot for the net harvest. In April 2015, the rachises were retrieved from the 27 nets (11+4 without dieback and 11+1 severely affected by *H. fraxineus*) and were placed in large plastic~~s~~ boxes in contact with moistened filter paper. The boxes were then closed and placed in the laboratory at room temperature. After a 5-week incubation, the rachises were examined for presence of *H. fraxineus* apothecia. The total length of each rachis was determined as well as the length between the two most distant apothecia of the rachis (hereafter called L.apo). As the part of rachises colonised by *H. fraxineus* was usually covered with many primordia of apothecium in the humid chamber, this is equivalent to the length of rachis colonised by *H. fraxineus*. The

percentage of rachis colonisation on a sapling was determined as $\sum$ *L.apo* / $\sum$ *total rachis length*. The saplings were again assessed in summer 2015 to determine the global infection status and the presence of shoot infection on the marked shoot assessed in 2014 for leaf infection.

The likelihood of leaf necrosis in September 2014 and shoot infection in summer were assessed by logistic regression with the plot, the dieback status of the saplings and their interaction as independent variables. We used a quasibinomial distribution to analyse leaf necrosis likelihood because data showed a dispersion higher than expected for a binomial distribution (over dispersion)~~; this is a common procedure frequently used in logistic regression~~. The percent rachis colonisation by *H. fraxineus* in spring 2015 was analysed by beta regression using the betareg package. The stand*dieback status interaction was assessed for leaf necrosis but not for shoot infection in 2015 and ability of rachises to produce apothecia as very few saplings then remained in Seichamps.

Data analysis

During survey, we noticed that some of the rated shoots were very vigorous with many rated leaves and that they were seldom infected by *H. fraxineus* whatever the general situation in the stand. These very vigorous shoots were usually sprouts from the collar of severely affected saplings. Preliminary analysis showed that shoots with more than 20 leaves had on average 5.9% necrotic leaves and no shoot mortality, compared to average values of 16.0 % of necrotic leaves and 21.6% shoot mortality for the total dataset. Shoots with more than 20 leaves were thus removed from the data set prior to analysis (represent only 1.5% of the data). Two different models were developed. Bayesian models were fitted with Jags 4.3.0 emulated with R4.1.0 (R2jags library).

Relationship between leaf necrosis and shoot mortality

The first model aimed at exploring the relationship between leaf necrosis and shoot mortality at the individual shoot level and how this may be controlled by tree health and site tree cover. Data were thus kept at the shoot level for this model. A hierarchical Bayesian model was developed to assess symptoms on shoot n of sapling i in year t:

$$\mathrm{logit}(pl[n,i,t]) = \alpha_1 + \alpha_2 \times DS[i] + \alpha_3 \times TC[i] + Tree.l\,[i] + Env.l\,[i,t]$$

$$\mathrm{logit}(ps[n,i,t]) = \beta_1 + \beta_2 \times DS[i] + \beta_3 \times TC[i] + \beta_4 \times DEF[n,i,t] + \beta_5 \times pl[n,i,t] + Tree.s\,[i] + Env.s\,[i,t]$$

with pl[n,i,t] and ps[n,i,t] the respective probability of leaf necrosis and shoot mortality, DS[i] the ash dieback status (either no dieback, when crown rating was of 0 or 1 or with dieback, when crown status was of 2 or 3), TC[i] the percent tree cover within a radius of 100 m, DEF[n,i,t] the proportion of missing leaves at the autumn rating, Tree.l [i] and Tree.s [i] individual tree random factors and Env.l [i,t] and Env.s [i,t], plot[i]*year[t~~i~~] random factors. The Env.l [i] and Env.s [i] random factors were taken as proxies for the environment, combining meso/micro climate and plot conditions (host density, Infection history). The ash dieback status DS[i] was computed as the worst status observed on the sapling during the period of observation (from 2 to 7 years depending on the sapling). The defoliation level (DEF[n,i,t]) was assumed to influence the shoot mortality likelihood because Mc Kinney & al (2011) suggested that early defoliation may prevent the transfer of *H. fraxineus* from leaves to shoots and thus represent a tolerance mechanism.

Both pl[n,i,t] and ps[n,i,t] were assumed to follow binomial distribution. Non-informative priors were assigned to the model parameters according to a normal distribution N(0, 1e+05) or a uniform distribution U(0,100) for the standard deviation of the random factors Tree and Env. The model was fitted with a burn-in of 3000, for 50000 iterations with a thinning of 5.

Three parallels chains with different initial parameter values were run and convergence was checked by a Gelman-Rubin test (R_{hat} were close to 1). The model was checked by looking at the plot of deviance residuals versus predicted values.

Relationship between leaf necrosis or shoot mortality and climate

The second developed hierarchical Bayesian model aimed at exploring the relationship between leaf necrosis or shoot mortality with climate. For that model, data were pooled at the plot * year level. Like in the previous model, shoot mortality was assumed to derive from former leaf infection and the proxy for leaf infection was taken as the leaf necrosis probability. Length of ash dieback presence in the area was included in the model to explain the likelihood of leaf necrosis. The year of first report of ash dieback by the health survey system is available by 16 x 16 km quadrat over France from data of the health survey system (Département de la Santé des Forêts, Fig 1). The number of years since the report of ash dieback in the local quadrat was taken as a proxy for the length of the disease presence. It was considered an adequate proxy as local spread of *H. fraxineus* at the scale of a village was shown to be very fast (Grosdidier *et al*, 2020). Leaf necrosis likelihood was assumed to depend on spring and summer climatic conditions (total rainfall and average temperatures). It is known that *H. fraxineus* may not survive well during hot summers (Hauptmann *et al*, 2013). In particular, this pathogen is known to stop its growth at temperatures above 28°C and to have limited survival when temperature exceeds 36°C. To account for this, we used TX78, the average daily maximal temperature in July-August (°C). Shoot mortality was assumed to depend on autumn climatic conditions (total rainfalland average temperatures). The meteorological data used were Safran data from Météo-France. Safran data are computed on a 8 x 8 km grid over France (Quintana Seguı & al, 2008). The Safran point closest to each site was selected and daily air temperature and rainfall data were retrieved for the studied years. All predictors were normalised to obtain zero mean and one standard deviation. However, the odds ratio reported in the text are in the non-normalized scale.

In a first step, the suitable periods for spring and autumn were selected by a preliminary analysis. For leaf necrosis a beta regression with a logit link function was used, as the number of observed leaves per site was very high (from 200 to 400). For shoot mortality, a logistic regression with a logit link was used. The site was included as a random factor in both models as well as the frequency of leaf necrosis in the site*year for shoot mortality. The model were fitted using the glmmTMB library. The explored climate variables targeted either the ascospore production in late spring or the development of shoot necrosis during the autumn. As the adequate period for ascospore production or for the development of shoot necrosis is not known precisely, several periods were tested: from 15 May to 15 June, for the 1 to 30 June or from the 15 June to 15 July for spring and September to October or September to December for autumn. For both models, climatic parameters included mean temperature and total rainfall. All predictors were normalised to obtain zero mean and one standard deviation. However, the odds ratio reported in the text are in the non-normalized scale.

In a second step, tThe following hierarchical Bayesian model was fitted (Figueroa-Zúñiga & al, 2013):

$$\text{logit}(pl[i,t]) = \alpha_1 + \alpha_2 \times RA6[i,t] + \alpha_3 \times TM6[i,t] + \alpha_4 \times RA78[i,t] + \alpha_5 \times TX78[i,t] + \alpha_6 \times LDP[i,t] + Site.l\ [i]$$

$$\text{logit}(ps[i,t]) = \beta_1 + \beta_2 \times RA912[i,t] + \beta_3 \times TM912[i,t] + \beta_4 \times TC[i] + \beta 5 \times pl[i,t] + Site.s\ [i]$$

with pl[i,t] and ps[i,t] the respective likelihood of leaf necrosis and shoot mortality in plot i and year t, RA6[i,t] and TM6[i,t] the sum of rain and mean daily air temperature in June, RA78[i,t] and TX78[i,t] the sum of rain and average daily air maximal temperature in July-August (°C), LDP[i,t] the number of years of ash dieback presence in the area, RA912[i,t] and TM912[i,t], the

sum of rain and mean daily air temperature in September, October, November and December and TC[i] the percent tree cover within a radius of 100m. Site.l [i] and Site.s [i] are site random factors, taken as a proxy for the site environment (micro climate, site conditions such as host density or Infection history).

While ps[i,t] was assumed to follow a binomial distribution, pl[i,t] was assumed to follow a beta distribution as the number of observed leaves per site was very high (from 200 to 400). Non-informative priors were assigned to the model parameters according to a normal distribution N(0, 1e+05) or a uniform distribution U(0,100) for the standard deviation of the random factors. The model was fitted with a burn-in of 10000, for 80000 iterations with a thinning of 10. Three parallel~~s~~ chains with different initial parameter values were run and convergence was checked by a Gelman-Rubin test (R_{hat} were close to 1).

The suitable periods for spring and autumn were selected in a preliminary analysis. The explored periods targeted either the period of ascospore production in spring or of the development of the shoot necrosis during the autumn. Several periods were tested: from 15 May to 15 June, for the 1 to 30 June or from the 15 June to 15 July for spring and September to October or September to December for autumn. The model showing the best DIC was selected.

The final model was checked by looking at the plot of deviance residuals versus predicted values and by a cross validation procedure. Data were randomly separated in 5 equal groups and the model was fitted using the data from 4 of the groups; predicted leaf necrosis frequency and shoot mortality were computed for the fifth group not used for the fit. The procedure was repeated five time and the observed value obtained by the procedure were plotted against the predicted values.

Relationship between climatic parameters and ability of *H. fraxineus* to produce apothecia in the fructification assay

The proportion of rachises producing apothecia in the fructification assay was analysed by beta regression using the glmmTMB library. The site was included as random factor. Fixed factors were the proportion of leaves with necrosis at the September rating, climatic parameters in the studied sites during the summer (total rainfall and mean of the daily maximal temperature in July and August) and climatic parameters during the overwintering period (average temperature and total rainfall at the overwintering locations from the beginning of October to the end of March).

Results

~~Altogether, d~~Data were available for 55 plot*year combinations for leaf necrosis frequency. Less data were available for shoot mortality (44 site*year combinations) because some plots were lost during winter (logging, 4 plots) or were not rated in spring (Table S1). Altogether, *H. fraxineus* was detected by the qPCR assay in 86% of the 63 tested leaves with necrosis. However, the result depended on the plot and year, with lower detection frequency in plots with very few leaf necrosis (75% of tested leaves with necrosis when overall leaf necrosis frequency was lower than 5%; 84% when it was between 5 and 15%; and 95% when it was higher than 15%). ~~So, the assessment of leaf necrosis frequency is correct when leaf necrosis is very frequent, but may be slightly overestimated when it is very low.~~

Ability of *H. fraxineus* to complete its cycle on ash saplings with different dieback severity

This section refers to the specific study done in two of the plots, Gremecey and Seichamps in 2014-15. Most of leaf symptoms that were observed in early September 2014 were partial leaf necrosis with one or several leaflet with necrosis but most leaflets still green. No shoot

mortality was observed in September 2014, the mortality occurring later, during the winter. The likelihood of leaf necrosis was significantly higher in Gremecey than in Seichamps (likelihood Chi-square= 4.55, p= 0.033, 38.0 ± 6.5 % in Gremecey and 20.6 ± 4.4 % in Seichamps).The percentage of leaves with necrosis ~~in early September 2014~~ was similar on saplings showing either no or severe dieback (dieback score of 0-1 versus 3, likelihood Chi-square of 0.7084, p= 0.400, Fig 2a). This was observed in the two studied plots as ~~The likelihood of leaf necrosis was significantly higher in Gremecey than in Seichamps (likelihood Chi-square= 4.55, p= 0.033, 38.0 ± 6.5 % in Gremecey and 20.6 ± 4.4 % in Seichamps) but~~ the plot * dieback status interaction ~~was~~ had no~~t~~ significant effect on leaf necrosis frequency (likelihood Chi-square = 1.32, p= 0.251).

H. fraxineus was able to produce apothecia on rachis of both the saplings with no ~~or~~ and severe dieback at the same rate (crown status of 0-1 versus at least 3, likelihood Chi-square = 1.19, p= 0.276, Fig 2b). The saplings with no dieback experienced limited mortality of the marked shoot while the shoot mortality was high for saplings showing dieback (likelihood Chi-square = 6.99, p= 0.008, Fig 2c). In summer 2015, the crown status of the saplings had not changed, i.e. only very minor signs of *H. fraxineus* infection ~~was~~ were observed on saplings with no dieback symptoms in 2014. ~~The likelihood of shoot infection was significantly higher in with severe dieback than in those with no dieback (likelihood Chi-square = 6.99, p= 0.008, Fig 2c).~~

Relationship between leaf necrosis in fall and subsequent shoot mortality

Shoot mortality in spring was strongly related to the frequency of ~~leaf~~ leaves with necrosis ~~likelihood~~ at the end of the previous summer (Fig 3a, b, Table 1). It is noteworthy that a small proportion of shoots that showed no leaf necrosis in September ~~had~~ were nevertheless ~~a small likelihood to die~~dead in the next spring (Fig 3a). Shoot mortality was very different on ashes with different dieback status (Table 1, Fig 3a). The odds ratio for shoot mortality of an ash with dieback versus one without was 4.1 (95% credible interval [2.4, 7.0]). By contrast, leaf necrosis did not significantly depend on tree dieback status (Table 1, mean leaf necrosis frequency of 12.9 ± 3.8 % for ashes without dieback and 16.3 ± 6.3 % for ashes with dieback). Both leaf necrosis and shoot mortality were strongly affected by the tree random factor with a similar range of standard deviation (Table 1). This means that, even after other variables of the model are taken into account (% tree cover, dieback status), some of the rated ashes consistently showed more leaf necrosis or shoot mortality across years. This effect was of the same magnitude for leaf necrosis and for shoot mortality.

Shoot mortality was also significantly ~~affected~~ increased at higher~~by~~ tree cover (Table 1, Fig 3b), with an odds ratio of 1.2 (95% credible interval [1.0, 1.3]) per 10% tree cover. By contrast, leaf necrosis was not affected by tree cover (Table 1). The environment random factor induced a level of variability similar for leaf necrosis and shoot mortality (Table 1). As no meso-climate effect was included in this model, high environment random factors were ~~this was~~ expected.

Early defoliation was significantly associated with shoot mortality, with ~~increasing~~ shoot mortality ~~with~~ increasing with the defoliation level (Table 1). Defoliation appeared to be a consequence of infection by *H. fraxineus* and significantly increased with leaf necrosis likelihood (likelihood Chisq of 58.3, p< 0.0001).

Relationship between leaf necrosis or shoot mortality and climate

The preliminary analysis showed that the best fit for foliar necrosis occurred for climatic conditions in June although the difference was minor (~~BIC~~ DIC of ~~103~~ 99.8 compared to D~~B~~IC

of ~~-~~100.9~~2~~ for 15 May to 15 June and ~~-97~~113.7 for 15 June to 15 July). Likewise, for shoot mortality, the best fit occurred for climatic conditions in September to end of December (~~BIC~~ DIC of ~~223~~ 99.8 compared to ~~BIC~~ DIC of ~~233~~ 103.5 for climatic conditions in September-October).

The model fitted adequately the data, with a proper residual distribution and an acceptable prediction of observed values in the cross-validation (Fig S1). The main parameter influencing leaf necrosis was TX78, the average of daily maximal temperature in July and August with a strong negative effect of high summer temperatures (Table 2, Fig 4a, odds ratio of 0.70, 95% credible interval CI [0.63, 0.84]). High average temperature in June increased the likelihood of leaf necrosis (Table 2, odds ratio of 1.33, CI [1.11, 1.52]). By contrast, rainfall either in June or in July-August was not related to leaf necrosis likelihood. Lastly, we observed a trend of decreasing leaf necrosis likelihood with the time of ash dieback in the area (Table 2, Fig 4c, odds ratio of 0.88, CI [0.83, 0.93]).

The shoot mortality was mainly linked to the average temperature in autumn, with mortality likelihood decreasing with increasing autumn temperatures (September to December, Table 2, Fig 4d, odds ratio of 0.69, CI [0.54, 0.86]). Rainfall in autumn was not related to shoot mortality (Table 2). As in the shoot level model, shoot mortality ~~was strongly linked~~increased significantly with ~~to~~ leaf necrosis frequency~~likelihood~~ ~~and increased with increasing tree cover~~ (Table 2~~, Fig. 4g~~). As a consequence, all parameters influencing leaf necrosis likelihood also influenced shoot mortality (Fig 4 e, f and g).

Ability of *H. fraxineus* to produce apothecia on overwintered rachises in the fructification assay

The presence of *H. fraxineus* in rachises at leaf fall was tested in 2020 and 2021 in part of the sites by qPCR. It showed striking differences between years and locations (Table 3). Moreover, it was not significantly linked to both ~~neither to~~ the leaf necrosis frequency measured in September, two months before (Pearson R= 0.208, pvalue= 0.440) ~~nor to~~and the subsequent shoot mortality (Pearson R= 0.266, pvalue= 0.320). In Champenoux, although the frequency of leaf necrosis was low both in 2019 and 2020, the proportion of rachis in which *H. fraxineus* was detected by qPCR was very different between the two years (Table 3). Another striking feature was that the frequency of *H. fraxineus* detection by qPCR in rachises at leaf fall was almost ten-fold higher than the frequency of necrotic leaves.

The proportion of rachises producing apothecia in the fructification assay in laboratory conditions showed very high variability, with a range of 0.01 to 0.99. This strongly depended on the year with mean values of 0.26 ± 0.11 in 2016, 0.52 ± 0.17 in 2017, 0.79 ± 0.09 in 2019 and 0.37 ± 0.23 in 2020. The proportion of rachises producing apothecia was significantly correlated to the proportion of rachises in which *H. fraxineus* was detected by qPCR at leaf fall (Pearson R= 0.710, pvalue= 0.002).

The proportion of rachises producing apothecia in the fructification assay was not significantly related to the frequency of leaf necrosis in the previous September rating (Fig 5a). It was not significantly related to the tested climatic parameters during the overwintering period (Table 4). By contrast, it was significantly related to the rainfall during the previous summer (sum of rainfall in July-August, Table 4, Fig 5b). It was also not related to the % of tree cover (Table 4).

Climate suitability in France for the different stage of *H. fraxineus* life cycle

Maps were produced using the developed models (Table 2 and 4) for an average site (site random factor fixed at 0) and for years 2010 to 2020. *H. fraxineus* should be able to colonise leaves, be present on rachises shed in autumn and be able to produce apothecia in spring in most of France (Fig 6a). By contrast, its ability to produce damage should be much more limited, in particular in southern France (Fig 6b, c and d). However, its ability to cause damage to ash trees is strongly dependent on local site conditions: comparison of Fig 6c and Fig 6d show large difference of prediction shoot mortality likelihood depending on the % tree cover. Uncertainty in parameter estimation very significantly affected both the maps of necrotic leaves / shoot mortality probability (Fig S2).

Discussion

We showed that carrier trees without crown dieback symptoms~~healthy carrier~~ exist and play a significant role in ash dieback epidemiology (here after referred to as "healthy carriers"). *H. fraxineus* is able to establish on ash leaves and to reproduce on the leaf debris in the litter (rachises) in situations where it induces very limited shoot mortality. This can be on ash trees that show no dieback while their neighbors show some (Fig 2 and 3a), ~~either~~ in specific local environment (low tree cover, Fig 3b) or meso-climates (Fig 6) ~~or specific trees where it induce very limited shoot mortality.~~ Climate strongly influence *H. fraxineus* with different parameters being important depending on the life cycle stage (Fig 4, Fig 5). As a consequence, while the pathogen might be able to establish over most France, it should ~~not be able~~ to induce far less severe~~significant~~ dieback in large parts of southern France (Fig 6). This is likely to occur in others area of southern Europe. Last, we observed a decreasing trend of severity (leaf necrosis and shoot mortality likelihood) with the time of disease presence (Fig 4 DH).

We were able to predict the severity of dieback in spring by observing leaf symptoms in the previous autumn. Leaf necrosis frequency in September was a good predictor of subsequent shoot mortality (Fig 3). Despite that, leaf necrosis was a very poor predictor of rachises infection at leaf fall and of the ability of *H. fraxineus* to produce apothecia, and therefore inoculum, on the rachises in spring (Fig 5, Fig 7). This may be because of the two-month delay between leaf necrosis measurement and rachises colonisation assessment. It has to be pointed out that very little inoculum production is observed September and October in French conditions (Grosdidier *et al,* 2018). Nevertheless, lesions present in the leaflets might have extended in the rachises. Another likely explanation is that *H. fraxineus* has the ability to asymptomatically infect ash leaves in early summer and to behave as a latent pathogen for an extended period (Cross *et al*, 2017). These authors showed that leaf necrosis occurs only when *H. fraxineus* inoculum reaches a certain level in the leaf which happens at the same time as leaf senescence and of a change in the leaf microbiota composition. It would appear that the high *H. fraxineus* levels in leaves required for leaf necrosis also strongly promote shoot infection from the petiole. By contrast, high *H. fraxineus* levels may not be required for rachis colonisation at leaf fall in senescent tissue.~~;~~ T~~t~~his would explain that high rachis colonisation may be observed in situations with limited previous leaf necrosis.~~the observed result~~. ~~Also, we examined only branches within reach to assess the disease severity, which may not be representative of disease severity higher in the crown. However, spore load in the air was shown to decrease from 1 to 3 m above soil level (Chandelier *et al*, 2014) and it is not likely that limited leaf infection occurs within soil reach while much higher infection is present in the tree crown, especially because microclimate is more humid close to soil and thus more favourable to *H. fraxineus* infection.~~

Because high rachis colonisation may occur while ~~of this difference between~~ symptom frequency~~induction~~, leaf necrosis or shoot mortality, is low (Table 3)~~and rachis colonisation~~, *H. fraxineus* is able to complete its biological cycle in conditions where no significant dieback is observed and some ashes behave as healthy carriers (Fig 7).

This is the case at local level, on specific trees that ~~retain healthy crown~~remain without shoot mortality despite experiencing leaf necrosis and enabling the pathogen to complete its cycle on infected rachises (Fig 2). We did not find difference in leaf necrosis frequency between saplings showing dieback or not (Table 1). The measure of leaf infection might not have been optimal as we only rated the presence of necrosis on the leaf without any measure of necrosis severity such as number of affected leaflets. Also, we examined only branches within reach to assess the disease severity, which may not be representative of disease severity higher in the crown. Leaf infection might be higher close to soil level as spore load in the air was shown to decrease from 1 to 3 m above soil level (Chandelier *et al*, 2014) and because microclimate is more humid close to the soil and thus more favourable to *H. fraxineus* infection. Our experimental design did not allow to determine whether the~~se~~ trees that did not show dieback did so for genetic reasons or because they were in microsite unfavorable to the disease or ~~because~~ harbored foliar microbial competitors preventing *H. fraxineus* development. Resistance toward *H. fraxineus* has been widely documented in *F. excelsior* (McKinney *et al*, 2011, Semizer-Cuming *et al*, 2021) and thus remains a very likely explanation for at least part of the observed pattern, although different micro-climatic conditions can be another one. It does not appear that some of the studied trees remained ~~healthy~~ without dieback because they avoided inoculum as they showed a frequency of leaf necrosis similar to trees that showed dieback symptoms in the same plots. In the two stands studied in more detail in 2014, the ash density was high and the saplings remaining ~~healthy~~ without dieback were in close proximity to dying saplings, questioning the possibility that they were experiencing different environmental conditions. Nevertheless, we do have evidence that environmental conditions strongly influence~~d~~ the ability of *H. fraxineus* to infect the shoot from the leaf petiole. Indeed, although the likelihood of leaf necrosis did not depend on the tree cover, the subsequent likelihood of shoot mortality was much lower in hedges with a low tree cover compared to forest plots with a high tree cover (Fig 3b). We also showed that the colonisation of rachises at leaf fall and subsequent ability of *H. fraxineus* to complete its cycle on the colonised rachises does not depend on the tree cover (Table 3), being similar in forests conditions and in hedges in open landscape. Hence, trees with no dieback symptoms in infected stands behave as heathy carriers, allowing *H. fraxineus* to reproduce on their leaves as efficiently as on the leaves of neighboring trees that experience dieback. They should even be much better inoculum producers than trees affected by dieback as they retain full foliage and thus produce abundant infected rachises. This result is in agreement with the observation of Grosdidier *et al* (2020) that the density of infected rachises was similar in the litter beneath ash trees in hedges and in forest settings although the trees showed far less dieback symptoms in the latter situation. A possible explanation is that as the tree cover does not affect inoculum production, it does not affect infection frequency. However, at low tree cover, crowns are exposed more frequently to ~~temperature~~ micro-climatic conditions adverse to *H. fraxineus* (hotter and dryer conditions, Grosdidier *et al*, 2020), preventing the increase of the pathogen present as endophyte in leaves and thus later shoot mortality. This discrepancy between the ability to reproduce on the host and the ability to induce significant damages has been reported for other tree pathogens. For example, *Diplodia sapinea* mainly produce inoculum on pine cones and is widely present on cones in healthy pine stands; it however needs weakening of the trees to induce significant damages (Palmer *et al*, 1988, Blodgett *et al*, 1997).

At a larger scale, we showed that *H. fraxineus* may be able to fulfil its cycle in regions were climate does not allow significant dieback (Fig 6). The disease has been progressing very regularly in France from 2009 to 2016 with a very noticeable slowdown of the spread in southern France from 2017 to 2019 (Fig 1~~b~~). The slowdown of the spread occurred in regions with climate unfavorable to ash dieback. In 2020, the Pyrenean area was colonised while no sign of the disease had been reported north in the Garonne valley (data from the Département de la Santé des Forêts). It is possible that the pathogen was introduced via infected planted ashes (Wylder *et al*, 2018). However, our results show that the spread without dieback symptoms of *H. fraxineus* in the Garonne valley is also a likely hypothesis. This may have been enhanced by the very hot summers of 2018-20, which were even less favorable to *H. fraxineus*-induced shoot mortality. The delay of 3-4 years observed until the first report of ash dieback in the Pyrenean piedmont is in line with the spread speed observed in France of about 60 km per year (Grosdidier, 2017).

The decreasing trend of leaf necrosis and shoot mortality likelihood with years of disease presence may be very significant but requires confirmation. It may well be a spurious result caused by the very dry and hot climate experienced in northern and eastern France in 2018-20, at the end of the study period. Climatic conditions were taken into account in our model and summer high temperature was one of the most important feature to explain symptoms severity. But the parameter we used (average daily maximal temperature) may not have accounted to entire effect of heat waves observed during this 2018-20 period. Another possible explanation is the progressive selection of more tolerant ashes in sites affected by ash dieback since a long time. Indeed, in the site followed for the longest period, less than 50% of the seedlings selected in 2015 were still alive in 2021. We tried to replace dead saplings by symptomatic saplings to avoid a progressive increase in tolerant ash frequency in our sample, but this was not always possible. ~~Alternatively, other environmental features might have changed such as the microbiota associated with leaves or rachises in the litter (Desprez-Loustau *et al*, 2019).~~

Early leaf senescence was hypothesized to be a mechanism of tolerance to *H. fraxineus*, the pathogen then being shed in the leaves before getting the chance to infect shoots (Mc Kinney *et al*, 2011, Schwanda & Kirisits, 2016). We were not able to confirm the hypothesis~~,~~ as early defoliation ~~being~~ was associated with higher shoot mortality in our data. However, care~~s~~ has~~ve~~ to be taken when interpreting this result. Mc Kinney *et al* (2011) reported that some ash trees show early leaf senescence as a genetically controlled trait, and thus show early defoliation, limiting the transfer of *H. fraxineus* from leaves to shoots. In our experiment, early defoliation was a result of infection, being tightly associated with high leaf necrosis frequency. In these conditions, transfer of the pathogen from leaves to shoots may not be hampered. This thus likely represent a different mechanism.

Climate appears to be a major driver of symptoms induction by *H. fraxineus*. ~~It was expected that h~~High summer temperatures ~~would be associated with~~was expected to limit~~ed~~ disease severity, as the pathogen has been shown to have low survival at temperature above 35°C (Hauptman *et al*, 2013). Moreover, ~~and to be limited in South-East of France by~~ the hot summers of the Rhône valley south of Lyon limit ash dieback severity in South-East France (~~Hauptman *et al*, 2013,~~ Grosdidier *et al*, 2018). More surprising is the low correlation of spring and summer rainfall with disease severity as it has been associated with high disease impact through increased inoculum production (Dvořák *et al*, 2016, Skovsgaard *et al*, 2017, Chumanová *et al*, 2019). Spring and summer rainfall may promote infection of the leaves~~,~~ while~~but~~ *H. fraxineus* ~~can then~~ remains as an asymptomatic endophyte especially if high summer temperatures prevent the heavy colonisation of ash leaves by the pathogen. This could explain why spring and summer precipitations were not well correlated to ~~the induction~~frequency of symptoms such as leaf necrosis and shoot mortality (Table 2). By

contrast, summer rainfall was strongly associated with both high rachis~~es~~ infection and ability to produce apothecia in the following spring. For example, in 2019, very limited leaf necrosis occurred in the Champenoux area because of high summer temperatures although summer rainfall allowed good colonisation by *H. fraxineus* of the rachises shed during the autumn (Table 3). This might be the likely mechanism by which spring and summer rainfall favour high ash dieback impact. The period in spring with the best fit between climatic conditions and leaf necrosis likelihood was in June, although the difference with other tested periods was low. This may appear surprising as the period of ascospores production has often been shown to be in summer, in July to August (Chandelier *et al*, 2014; Dvorak *et al*, 2016; Hietala *et al*, 2013; Timmermann *et al*, 2011). However, many of those results correspond to central or northern Europe; in North-East France, the period of ascospores production was earlier, from mid-June to mid-July (Grosdidier *et al*, 2018) and ascomata have been observed as early as the first week of May in the Pyrenean sites during this work. Shoot mortality was associated with previous leaf necrosis, but also to subsequent mild autumn temperature. This is the first report of an~~This~~ association of shoot mortality with autumn climatic conditions ~~was by now never reported in the literature~~ and the mechanism involved remains to be elucidated.

H. fraxineus ability to infect ash leaves and reproduce on the rachises in the forest litter does not appear~~s~~ to limit the pathogen ability to establish throughout France. By contrast, the low ability to induce shoot mortality ~~is~~ explains the absence of the disease report in many areas of southern France~~the limiting step~~. Hence, the maps showing the shoot mortality likelihood match well with the ash dieback risk predicted by Goberville *et al* (2016). The statistical niche model produced by these authors was based on the distribution in Europe of this invasive pathogen still spreading and with a presence still limited to North-Easter France; it nevertheless well captured the potential climatic niche in France. This would confirm that south-eastern France is not favorable to ash dieback, as was suggested by Grosdidier *et al* (2018).

Acknowledgement

We wish to thank Olivier Caël and Anaïs Gillet for technical assistance in handling the analysis of rachises ability to produce apothecia and level of infection by *H. fraxineus*. The establishment and rating of the plots in the Charente area was done by Dominique Piou while those of plots from Brittany was done by Laurence Roche from the Département de la Santé des Forêts. We also thank Mireia Gomez-Gallego for her advices in the analysis and for reviewing the manuscript and the two anonymous reviewers that helped improve the manuscript. This work was funded by the French Forest Health Department, the French Ministry in charge of Agriculture and Forestry and by the project HOMED from the European Union's Horizon 2020 Program for Research & Innovation under grant agreement No 771271. The UMR1136 research unit is supported by a grant managed by the French National Research Agency (ANR) as part of the "*Investissements d'Avenir*" program (ANR-11-LABX-0002-01, Laboratory of Excellence ARBRE).

Reference

Alizon S, Michalakis Y. Adaptive virulence evolution: the good old fitness-based approach. Trends Ecol. Evol. 2015; 30:248–54.

Blodgett JT, Kruger EL, Stanosz GR. *Sphaeropsis sapinea* and Water Stress in a Red Pine Plantation in Central Wisconsin. Phytopathology, 1997; 87:429–434.

Chandelier A, Helson M, Dvorak M, Gischer F. Detection and quantification of airborne inoculum of *Hymenoscyphus pseudoalbidus* using real-time PCR assays. Plant Pathology. 2014; 63:1296–1305.

Chumanová E, Romportl D, Havrdová L, Zahradník D, Pešková V, Černý K. Predicting ash dieback severity and environmental suitability for the disease in forest stands. Scandinavian Journal of Forest Research. 2019; 34:254–266.

Cross H, Sønstebø JH, Nagy NE, Timmermann V, Solheim H, Børja I, et al. Fungal diversity and seasonal succession in ash leaves infected by the invasive ascomycete *Hymenoscyphus fraxineus*. New Phytol. 2017 213:1405–1417.

Cunniffe NJ, Koskella B, E. Metcalf CJ, Parnell S, Gottwald TR, Gilligan CA. Thirteen challenges in modelling plant diseases. Epidemics 2015; 10:6–10.

Davidson JM, Wickland AC, Patterson HA, Falk KR, Rizzo DM. Transmission of *Phytophthora ramorum* in Mixed-Evergreen Forest in California. Phytopathology 2005; 95:587–596.

~~Desprez-Loustau M-L, Hamelin FM, Marçais B. The ecological and evolutionary trajectory of oak powdery mildew in Europe. In Fenton A, Tompkins D editors. Wildlife Disease Ecology. Cambridge University Press; 2019. p. 429-57.~~

DiLeo MV, Bostock RM, Rizzo DM. *Phytophthora ramorum* Does Not Cause Physiologically Significant Systemic Injury to California Bay Laurel, Its Primary Reservoir Host. Phytopathology 2009; 99:1307–1311.

Dvorak M, Rotkova G, Botella L. Detection of Airborne Inoculum of *Hymenoscyphus fraxineus* and *H. albidus* during Seasonal Fluctuations Associated with Absence of Apothecia. Forests 2013; 7:1.

Figueroa-Zúñiga JI, Arellano-Valle RB, Ferrari SLP. Mixed beta regression: A Bayesian perspective. Computational Statistics & Data Analysis 2013; 61:137–147.

Flowers J, Nuckles E, Hartman J, Vaillancourt L (2001). Latent Infection of Austrian and Scots Pine Tissues by *Sphaeropsis sapinea*. Plant Disease 2001: 85:1107–1112.

Garbelotto M, Schmidt D, Swain S, Hayden K, Lione G. The ecology of infection between a transmissive and a dead-end host provides clues for the treatment of a plant disease. Ecosphere 2017; 8:e01815.

Goberville E, Hautekèete N-C, Kirby RR, Piquot Y, Luczak C, Beaugrand G. Climate change and the ash dieback crisis. Scientific Reports 2016; 6:35303.

Grosdidier M. Épidémiologie de la chalarose du frêne, une maladie causée par l'agent pathogène *Hymenoscyphus fraxineus* [dissertation]. Nancy : Université de Lorraine (PhD, in french) ; 2017.

Grosdidier M, Ioos R., Husson C, Cael O., Scordia T, Marçais B. Tracking the invasion: dispersal of *Hymenoscyphus fraxineus* airborne inoculum at different scales. FEMS Microbiology Ecology, 2018; 94:fiy04.

Grosdidier M, Ioos R, Marçais B. Do higher summer temperatures restrict the dissemination of *Hymenoscyphus fraxineus* in France? For. Path. 2018; e12426.

Grosdidier M, Scordia T, Ioos R, Marçais B (2020) Landscape epidemiology of ash dieback. J. Ecol. 2020; 108:1789–1799.

Gross A, Holdenrieder O, Pautasso M, Queloz V, Sieber TN. *Hymenoscyphus pseudoalbidus*, the causal agent of European ash dieback. Molecular Plant Pathology 2014; 15:5–21.

Hauptman T, Piškur B, de Groot M, Ogris N, Ferlan N, Jurk D. Temperature effect on *Chalara fraxinea* : heat treatment of saplings as a possible disease control method. For. Path. 2013; 43:360–370.

Hietala AM, Timmermann V, Børja I, Solheim H. The invasive ash dieback pathogen *Hymenoscyphus pseudoalbidus* exerts maximal infection pressure prior to the onset of host leaf senescence. Fungal Ecol. 2013; 6:302–308.

Holdenrieder O, Marco P, Weisberg PJ, Lonsdale D. Tree diseases and landscape processes: the challenge of landscape pathology. Trends in Ecology & Evolution 2004; 19: 446–452.

Ioos R, Kowalski T, Husson C, Holdenrieder O. Rapid in planta detection of *Chalara fraxinea* by a real-time PCR assay using a dual-labelled probe. Eur. J.Plant Path. 2009; 125:329–335.

Kelnarová I, Černý K, Zahradník D, Koukol O, Sieber T. Widespread latent infection of *Cryptostroma corticale* in asymptomatic *Acer pseudoplatanus* as a risk for urban plantations. For. Path. 2017; 47:e12344.

Kowalski T, Holdenrieder O. The teleomorph of *Chalara fraxinea*, the causal agent of ash dieback. For. Path. 2009; 39:304–308.

Landolt J, Gross A, Holdenrieder O, Pautasso M. Ash dieback due to *Hymenoscyphus fraxineus* : what can be learnt from evolutionary ecology? Plant Path. 2016; 65:1056–1070.

McKinney LV, Nielsen LR, Hansen JK, Kjær ED. Presence of natural genetic resistance in *Fraxinus excelsior* (*Oleraceae*) to *Chalara fraxinea* (*Ascomycota*): an emerging infectious disease. *Heredity* 2011; 106:788–797.

McKinney LV, Thomsen IM, Kjaer ED, Nielsen LR. Genetic resistance to *Hymenoscyphus pseudoalbidus* limits fungal growth and symptom occurrence in *Fraxinus excelsior*. For. Path. 2012; 42:69–74.

Marçais B, Kosawang C, Laubray S, Kjaer E, Kirisits T. (2022). Ash dieback. In: Asiebu F, Kovalchuk, editors. Forest Microbiology Volume 2: Forest Tree Health. Academic Press, 2022. p. 215-237.

Marsberg A, Kemler M, Jami F, Nagel JH, Postma- Smidt A, Naidoo S et al.. *Botryosphaeria dothidea* : a latent pathogen of global importance to woody plant health. Molecular Plant Pathology 2017; 18:477–488.

Orton ES, Clarke M, Brasier CM, Webber JF, Brown JKM. A versatile method for assessing pathogenicity of *Hymenoscyphus fraxineus* to ash foliage. For. Path. 2019; 49:e12484.

Palmer M, Mcroberts R, Nicholls T. Sources of Inoculum of *Sphaeropsis sapinea* in Forest Tree Nurseries. Phytopathology 1988; 78:831–835.

Pagán I, García-Arenal F. Tolerance of Plants to Pathogens: A Unifying View. Annual Review of Phytopathology 2020; 58:77–96.

Quintana Seguı P, Le Moigne P, Durand Y, Martin E, Habets F, Baillon M et al. Analysis of near-surface atmospheric variables: Validation of the SAFRAN analysis over France, J. Appl. Meteorol. Climatol. 2008; 47:92–107.

Schwanda K, Kirisits T. Pathogenicity of *Hymenoscyphus fraxineus* towards leaves of three European ash species: *Fraxinus excelsior*, *F. angustifolia* and *F. ornus*. Plant Pathology 2016; 65:1071–1083.

Semizer-Cuming D, Chybicki IJ, Finkeldey R, Kjær ED. Gene flow and reproductive success in ash (*Fraxinus excelsior* L.) in the face of ash dieback: restoration and conservation. Annals of Forest Science 2021; 78:14.

Sieber TN. Endophytic fungi in forest trees: are they mutualists? Fungal Biology Reviews 2007; 21:75–89.

Skovsgaard JP, Wilhelm GJ, Thomsen IM, Metzler B, Kirisits T, Havrdová L et al. Silvicultural strategies for Fraxinus excelsior in response to dieback caused by *Hymenoscyphus fraxineus*. Forestry 2017; 90:455–472.

Timmermann V, Børja I, Hietala AM, Kirisits T, Solheim H. Ash dieback: pathogen spread and diurnal patterns of ascospore dispersal, with special emphasis on Norway. *EPPO Bull*. 2011 41:14–20.

Wylder B, Biddle M, King K, Baden R, Webber J. Evidence from mortality dating of *Fraxinus excelsior* indicates ash dieback (*Hymenoscyphus fraxineus*) was active in England in 2004–2005. Forestry: An International Journal of Forest Research 2018; 91:434–443.

Supporting Information:

Table S1. Studied sites

Fig S1. Cross-validation for the model at the plot level relating climatic and site parameters to leak necrosis and shoot mortality likelihood. A. Leaf necrosis, B. shoot mortality

Fig S2. Quantile 0.05 (Q5) and 0.95 (Q95) for the estimated likelihood of leaf necrosis (A, B) and shoot mortality (C, D). The map were computed using the parameter 0.05 and 0.95 quantiles obtained from the Bayesian procedure fit. The shoot mortality is computed for a forest situation (tree cover of 100%).

Figures captions

Fig 1. Location of the studied ash stands (black dot) relative to the year of first ash dieback report.

Data from the Département de la Santé des Forêts. Am, Fréchencourt, Be, Sarrance, Ch1-6, Champenoux (6 plots), Co, Colombier, Fe, Ance-Féas, Gr, Gremeçey, La, Landivisiau Lu, Lupé, Ol1-2, Oloron-Sainte-Marie (2 plots), Po, Pont-de-Buis, Ro, Roche-sur-Grane, Sa, Salignac-sur-Charente Sc, Scaër, Se, Seichamps). The region border shapefile can be uploaded at https://geoservices.ign.fr/telechargement.

Fig 2. Impact and ability of *H. fraxineus* to complete its cycle on ash saplings with either no or severe dieback.

(A) % leaves with necrosis in autumn 2014 (likelihood Chi-square of 0.7084, p= 0.400). (B) % of rachis length with presence of *H. fraxineus* apothecia in April 2015 (likelihood Chi-square = 1.19, p= 0.276). (C) % of rated shoot with bark infection in summer 2015 (likelihood Chi-square = 6.99, p= 0.008).

Fig 3. Modelled and observed shoot mortality for ~~Ash~~ ash trees of different level of dieback

(A) Site with different dieback status (with dieback, score 0-1 or without, score of 2-3). (B) Sites with different tree cover. Points represent the observed data, pooled by increasing 10% leaf necrosis frequency. For tree cover, blue dots represent sites with 9-25 tree cover while red dots represent sites with 75-100 % tree cover. Points representing less than 10 shoots are not represented.

Fig. 4. Modelled and observed leaf necrosis and shoot mortality depending on climatic parameters and sites conditions.

The curves represent the predicted response for a given parameter, all other parameters being fixed at the average value, except for the ~~Number~~ number of year of ash dieback presence that is set to 1. The orange area (grey in printed version) represent the 95% confidence interval. ~~The d~~Dots represent oberved values.

Fig. 5. Ability of *H. fraxineus* to produce apothecia on the ash rachises overwintered in common garden (fructification assay).

(A) Relation with leaf necrosis observed in the same plot two months before (pvalue= 0.304). (B) Relation with climate (pvalue= 0.006, Table 4).

Fig. 6. Map of climate suitability for *H. fraxineus* ability to produce apothecia on the ash rachises or symptoms on leaf and shoot of ~~Ash~~ ash trees.

Safran meteorological data, 2010-20. (A). Proportion of ~~Ash~~ ash rachises collected in fall producing apothecia in laboratory conditions after overwintering in a common garden (fructification assay). (B) Leaf necrosis likelihood. (C) Shoot mortality likelihood in open canopy (20% tree cover). (D) Shoot mortality likelihood in forest conditions (100% tree cover).

Fig.7. Hypothesised impact of studied environmental parameters on *H. fraxineus* life cycle.

Higher summer rainfall and higher spring temperatures are associated with higher rates of leaf necrosis leading to higher shot necrosis especially in high tree cover.~~High summer rainfall and spring temperature are associated with high presence of the pathogen in leaves in summer, presumably throughout enhanced apothecia production and leaf infection~~. ~~Moderate~~ Higher summer temperatures ~~is~~ are associated with low leaf necrosis frequency at the end of the summer, but ~~does~~ not ~~affect the~~to low colonisation of rachises ~~that fall in the forest litter~~at leaf-fall during autumn. ~~Moderate~~ Low autumn temperatures are~~is~~ associated with high shoot mortality~~, presumably because~~ (better transfer from leaves to shoot or lesion development in shoots ?)~~are favoured~~. The part of the cycle needed for inoculum production is indicated in blue while red arrows and text refers to symptoms development that does not lead to inoculum production. Cycle adapted from Marçais *et al* (2022).

Table 1. Relationship between leaf necrosis and shoot mortality at the shoot level. Parameters of the hierarchical Bayesian model

Variable	Leaf necrosis [95% credible interval]	Shoot mortality [95% credible interval]
Sapling dieback status (DS)	0.27 [-0.01, 0.55]	**1.27 [0.82, 1.75]**
Site tree cover (TC)	-0.01 [-1.14, 1.16]	**1.40 [0.07, 2.23]**
Defoliation at September rating (%,DEF)	-	**1.53 [0.38, 2.68]**
Probability of necrotic leaves (pl)	-	**1.75 [0.24, 3.27]**
Tree random factor (sd, Tree.l and Tree.s)	0.93 [0.82, 1.06]	0.89 [0.55, 1.24]
Site*year random factor (sd, Env.l and Env.s)	0.95 [0.65, 1.32]	1.22 [0.97, 1.54]
Deviance	4615 [4532, 4698]	

NOTE. Parameter with a 95% credible interval that does not contain 0 are considered significant and are marked in bold. Positive values indicate an association between high levels of both the symptom and of the environmental variable.

Table 2. Relationship between leaf necrosis or shoot mortality and climate parameter (plot level). Parameters of the hierarchical Bayesian model

Variable	Symptom	Parameter [95% credible interval]
Rain June (RA6, α_2)	Leaf necrosis	0.04 [-0.16, 0.25]
Temperature June (TM6, α_3)	Leaf necrosis	**0.69 [0.37, 0.99]**
Rain Junly-August (RA78 (α_4)	Leaf necrosis	-0.20 [-0.52, 0.11]
Average daily maximal temperature July-August (TX78, α5)	Leaf necrosis	**-1.11 [-1.50, -0.69]**
N year ash dieback presence (LDP, α6)	Leaf necrosis	**-0.37 [-0.58, -0.17]**
Site random factor (Site.l, sd)	Leaf necrosis	0.21 [0.01, 0.49]
Rain September-December (RA912, β_2)	Shoot mortality	-0.06 [-0.43, 0.29]
Temperature September-December (TM912, β_3)	Shoot mortality	**-0.83 [-1.30, -0.39]**
Site tree cover (TC, β_4)	Shoot mortality	**0.77 [0.07, 1.52]**
Probability of necrotic leaves (pl, β_5)	Shoot mortality	**4.55 [4.38, 8.12]**
Site random factor (Site.s, sd)	Shoot mortality	1.21 [0.68, 2.01]
Deviance	3745 [3667, 3826]	

NOTE. Parameter with a 95% credible interval that does not contain 0 are considered significant and are marked in bold. The variables are standardized to have zero mean and one standard deviation with the exception of pl, the leaf necrosis likelihood. Positive values indicate an association between high levels of both the symptom and of the environmental variable.

Table 3. Colonisation of rachises at leaf fall in November

	Year	Frequency of necrotic leaves in September	Frequency of *H. fraxineus* detection on rachises in November (qPCR)
Champenoux	2019	0.09 ± 0.03	0.87 ± 0.19
	2020	0.05 ± 0.02	0.35 ± 0.12
Pyrenean piedmont	2020	0.12 ± 0.05	0.60 ± 0.18

Table 4. Influence of climatic parameters on the ability of *H. fraxineus* to produce apothecia in the fructification assay

Variable	Parameter	Pvalue
Leaf necrosis frequency	-~~4.79~~0.44	0.~~304~~297
Rain in July-August (RA78)	~~1.41~~1.23	<0.001~~6~~
Mean maximal daily temperature in July-August (TX78)	-0.~~32~~29	0.~~411~~353
Rain in October-March (RA10-3 ow)	0.~~26~~21	0.~~613~~478
Mean temperature in October-March (TM10-3 ow)	0.~~46~~55	0.~~112~~089
Site tree cover (%, TC)	0.~~43~~11	0.~~444~~549
Site random factor (sd)	0.0003~~1~~	-

Note: RA10-3 ow and TM10-3 ow, sum of rain and mean of daily temperatures at the overwintering sites from October to March.
