# Supplementary material for: Ability of the ash dieback pathogen to reproduce and to induce damage on its host are controlled by different environmental parameters
Source: PLoS Pathog. 2023 Apr 20;19(4):e1010558. doi: 10.1371/journal.ppat.1010558 (PMC10153702; doi:10.1371/journal.ppat.1010558)
Supplement: Attachment — Submitted filename: Response_reviewers22.docx [file ppat.1010558.s006.docx]

Response to the reviewers :

Main points

- For tolerance versus Resistance. I feel we have been quite careful in the use of the terms. I personally agree with Landolt et al (2016) that individual ash that do not show dieback can be said to be tolerant. We showed that i. they appear to support similar pathogen load (similar leaf necrosis), ii. They do not restrict the pathogen ability to multiply (similar ability to produce apothecia on rachises). We nevertheless tried not using the term to qualify the ash we studied because we did not show that the phenomenon was controlled genetically (well developed in discussion, L465-477). The term tolerance is still present in introduction, for definition and then in the context of Landolt et al (2016) citation (L97-99). We here refer to their own word and I thus believe it is correct, especially because it is explicitly specified that no hard data exist. The sentence referring to Landolt et al (2013) was modified as asked by reviewer 2. Tolerance is then used in two other locations (material and method and discussion). Usually, it is to deal with the early leaf abscission that might prevent transfer of the pathogen from leaves to shoot. This is an accepted mechanism of tolerance for ash dieback.

- We agree that the « asymptomatic » word was very misleading to refer to the saplings showing no dieback symptoms, as it ended up dealing with asymptomatic individuals with necrotic leaves… We followed the suggestion of reviewer 1 referring to the symptom: we thus now have saplings with either no (crown rating of 0-1) or severe dieback (2-3) and we tried to stick to the terminology throughout the text. Likewise, we removed all the “decline”. “Asymptomatic” is still present, but in other context in introduction (diseases other than ash dieback) and in discussion (leaves with endophytic presence of *H. fraxineus* in literature).

- The fructification test used in the comparison of sapling with/without dieback symptoms was questioned. We believe that the test is correct. This is dealing with rachises in humid chamber. In these conditions, we do not have 2-10 apothecia per rachis easy to count, but usually several hundreds of apothecium primordia with only some that end up forming well developed apothecia. Deciding when a primordia become an formed apothecia is not straitforward. Thus, measuring the length between the farthest apothecia / primordia is equivalent to measuring the length of fruiting rachis and we considered it adequate. This is now indicated in the material and method (L216-220). For latter fructification tests, we end up taking the part of the rachis with pseudosclerotial plate instead, a more direct measure. Thus what we measure is the part of the rachis that is colonised and able to produce apothecia, but not the potential number of apothecia that may be formed. This now better explained (L173-179). Then, the number of rachis sampled for the test was also questioned and it was asked to clarify the sampling procedure. The sampling procedure was to collect one rachis at the base of each sapling, so 20 rachis per site*year. This is now indicated in the material and method (L162-164). The proportion of rachis that produce apothecia varies from 1 to 95% depending on the sites*year! With that range of variation, we feel confident 20 rachises per site*year is enough to have adequate results. The variability per climate condition remains in an acceptable range (see Fig 5). Then, no reference is given for the fructification test because it was developed independently of any others works, even if others did similar work in the meantime. Rachises were overwintered in common sites because we were mostly interested in the summer climatic conditions and it was easier to handle. Last, for the minor comment on decomposition of rachis with/without pseudosclerotial plate, no publish data really document this. In fact, we have unpublished data that suggests that rachis with/without pseudosclerotial plate decompose at the same rate.

- Data discarded and missing data. Questions were raised on the fact that we discarded data on very vigorous shoots (with more than 20 leaves, L234-240). This does not represent healthy vigorous saplings but instead sapling that were very severely affected and produce sprout from the collar after being almost killed. Then it is only 30 shoots located in 8 sites out of 1992 observed (1.5%). This is now indicated in the text (L240). We believe this improve the data as these very vigorous shoots on sapling with heavy dieback behaved strangely, but we checked that it almost does not modify the results if we do not discard them. Missing data occurred for shoot mortality (indicated in table S1). A big part of it is caused by logging activity on the sites (Seichamps 2015, Lupé 2021, Colombier and Salignac 2020). For the Bretagne sites, because of poor labeling procedure in autumn, we were not enable to find back the marked shoots in spring. For the last missing site (Roche-sur-Grane 2021), it was decided that the cost of traveling was not worth given that plenty of data was already available on shoot mortality in the hot dry conditions that the site experienced in 2020. We are confident that the missing data do not weaken our data set in a strong way. The climatic conditions well cover the range of possible climate conditions in France (We do have a sampling on 20 sites and across 9 years). Then the Bayesian procedure used is well adapted to cope with missing data.

Other remarks

- Most of the minor modifications asked by the reviewers were introduced and only the most important or those not introduced will be commented.
- Inoculum production in shoot (L87-91). We kept our wording. While we agree that conidia produced on shoot can be neglected as inoculum source, apothecia are occasionally produced on small shoot killed by *H. fraxineus*. The mechanism is usually without much consequence but explains how infected seedlings planted without leaves may spread the pathogen.
- For early leaf abscission as a mechanism of tolerance in Ash dieback. We added a sentence dealing with this mechanism in the introduction (L92-94). We agree with reviewer 2 that early defoliation in our experiment may not be related to the genetic trait of early leaf senescence. We tried to explain better (L525-534) as reviewer 1 reported the paragraph as unclear.
- Leaf lesions. Reviewer 2 report that leaf lesions shown in Gross et al 2014 can be small necrotic spots (L145-147). We disagree. While in one of the picture, small necrotic spots are present on the picture, both the arrows and the detail view of a leaf do point on lesion on margins and/or lesions extending along veins as typical *H. fraxineus* leaf lesions. Then our early qPCR tests clearly showed that such small necrotic spots were seldom associated with *H. fraxineus*.
- Figure 1 was changed as asked with plot added on the map of ash dieback progress in France and information on the temperature gradient transferred to table S1. Column were better labelled in Table S1.
- Sapling sampling. The procedure is now clarified. In fact, it was not completely random as if the starting point was random, the saplings were sampled in a contiguous patch afterward. We did not sample according to health status because the proportion of sapling with / without dieback was anyhow adequate to test our hypothesis (L130-134). In many plot, ash dieback was not present since a long time and many individuals healthy the first year anyhow exhibited strong dieback the second. Likewise, we replace dead saplings by some showing dieback and not randomly selected saplings to ensure this balanced proportion between saplings with / without dieback and keep the possibility to test our hypothesis on existence of healthy carrier.
- We attempted to better explain how to interpret different statistical features as asked by reviewer 2. This is not always easy as it could require lengthy development… For quasibinomial distribution and over dispersion, it would be completely out of the scope of the manuscript to go above what we added (L226-228) as this is a really common and standard practice in logistic regression. Not being statistician, we do not feel comfortable giving lengthy explanation and a quick search in Wikipedia would probably bring much more meaningful information than whatever we can add. Briefly, it says that there are significant variables influencing the result that are not included in the model, which is hardly a surprise for data collected *in natura*! To account for that, the quasibinomial model adds a scale factor that results in increased variance of the estimated model parameters and this reduces the likelihood that they are different from 0, making the test more stringent. We also added explanation in the note of hierarchical Bayesian model tables to explain that parameters with credible interval that does not contain 0 are considered significant. We added explanation on the biological interpretation of tree random factors (L364-367). We hope this clarifies the manuscript.
  Then, at several point, reviewers asked why we didn’t include interactions (between dieback status and tree cover, or between rain and temperature). The models are already rather complex and we did not want to increase that complexity to a too high degree.
- Normalization of variable. This is a standard practice in ecology for a number of reason. First, on a practical point of view, models converge better and with less autocorrelation in the MCMC chains. Then, it enable to directly compare the importance of variable in influencing the response as they are in the variable sd unit. I understand that it may not be practical if you want to interpret it in terms of 1°C increase foliar necrosis by X%. But then, in figures 3-5, the scale is for non-normalized variables which enable to assess that. To improve, we now report the odds ratio ant credible interval in it non-normalize scale for climatic variables (L383-394).
- We added a figure 7 to describe how we suppose feature of H. fraxineus cycle are affected by environment.
