# Supplementary material for: Ability of the ash dieback pathogen to reproduce and to induce damage on its host are controlled by different environmental parameters
Source: PLoS Pathog. 2023 Apr 20;19(4):e1010558. doi: 10.1371/journal.ppat.1010558 (PMC10153702; doi:10.1371/journal.ppat.1010558)
Supplement: Attachment — Submitted filename: Second_commentaire1122.docx [file ppat.1010558.s007.docx]

We want to thank you and reviewers for the constructive remarks on the manuscript and we introduced most of them. We tried to remove the typos mistakes as much as possible and hope there isn’t much left.

In particular, reviewer 1 pointed out that material and method did not really enable to understand if the same saplings were rated over several years and how that was taken into account in the analysis. We agree this was a major problem. First, we introduced the changes you suggested for the saplings selection and rating over years making now clear that the majority of saplings were rated several years. The overall replacement rate is 18% but it has to be pointed out that the saplings replaced are mostly saplings experiencing dieback. The saplings that show no dieback do not die frequently and usually stay in the sample for all the study period. Then, in the data analysis, we realized that it was not indicated that the dieback status of saplings that is taken into account is the worst dieback rating observed on each sapling during the study period. This is now indicated L260-2. Related to that, the way the model was specified was also not correct (L252-253). The respective probability of leaf necrosis and shoot mortality pl[i] and ps[i] should be indexed on n, the shoot, i the sapling and t the year. This is now done and it can be seen that the ash dieback status DS[i] does not depend on the year t. I hope this clarify properly the methodology.

Abstract:
Line 14: I suggest, "genetic resistance or tolerance to the disease".
Done

Author summary

Having read your paper thoroughly, I think the last sentence of the Author Summary is one of the most speculative points in the paper and could be omitted. It's potentially misleading for a non-specialist reader because in the paragraph starting on line 511, you acknowledge that this could be an artefact because of a recent run of dry summers in France. If you wish to use the space to mention another important conclusion instead, please do.
Done

Short title: Perhaps, "Environmental influences on ash dieback"? You consider environmental variables other than climate.
Done


Introduction

Line 91: "very limited inoculum production occurs on diseased shoots". This is slightly confusing because "shoots" includes the leaves on those shoots. Maybe, "occurs on the bark of diseased shoots" - is that what you mean?
Done

Line 97: I suggest "fallen leaves" to avoid giving the impression that the pathogens undergoes (sexual) reproduction on green leaves.
Done

Line 100: I suggest something like, "could prevent transfer of the pathogen from infected leaves to shoots but still allow sexual reproduction on fallen rachises". Also see lines 255-258.
Done

Lines 109-114: I feel the first sentence of this paragraph suggests that the emphasis of the paper is on discovering if ash trees are tolerant, while "studied environmental influence" is imprecise. Your data are indeed relevant to tolerance but this is a consequence of what you have discovered about environmental effects on different stages of the disease. I suggest rephrasing line 110 to, "we tested if different environmental variables favour important stages..." I also suggest deleting the last sentence of the paragraph (the key steps of the H.f. life cycle have already been characterised) and replacing it by something like, "We propose that healthy carriers, particularly tolerant genotypes of ash, could be favoured in locations where the environment promotes infection and reproduction of H.f. but not symptom development".
Clarified


Materials and methods

This is mostly fairly clear but some points could be clarified or made more precise.

Line 120: I suggest, "where ash dieback was first observed in 2009-10" (given that the disease may have been present for many years previously; note Wylder et al. 2018).
Done

Line 126: Does "tree cover" mean all trees or only ash? I believe you mean all species but please make this clear.
Done

Line 131: Was there any difference between saplings and "larger trees with low-lying branches"? Could it have influenced variation between plots?

We tried at one point to test whether the tree height could be a suitable co-variable and it did not stick out as significant. This is now added L132-133 without detail information.

Line 133: When you say, "without dieback symptoms", do you mean there were no dead twigs or shoots and you discounted lesions on leaves? (Noting that you go on to describe your rating system for saplings in terms of the proportion of dead wood.) If so, I suggest, "without dieback of shoots or twigs".
See later. Dieback just refers to perennial organ such as shoot and discount leaves (L136-7).

**Important point:** Lines 167-8: "we were mainly interested in the climatic conditions during rachis colonisation phase in June –October": does this mean you assumed that environmental conditions were not important during the overwintering stage (do we know what happens at that time - pseudosclerotia maturing, perhaps?) or in spring, when ascomata are developing? Similarly, lines 168-170: You used a standard set of conditions to produce apothecia in April and May, but surely local conditions during this time could be important in the life cycle of H.f.? Your decision to focus on conditions from June to October needs more justification than simply "we were interested".

We focused on the vegetation season because this is the time were inoculum production and leaf infection occurs and we presumed that it would be the main factor affecting latter rachis colonisation. This is now indicated in the text (L173-175). Of course, conditions during winter could influence the production of the pseudosclérotiale plate and thus have some importance. But, this would have added a travel to plots scattered over France. We did not have the resources to do that and had to limit our ambition… Then for the fructification assay, what is tested here is the potential of rachises to produce apothecia if they are in suitable conditions. We could not test everything in one study!

The point about rating samplings over 1-6 years and the important point about replacing saplings (lines 154-158) is part of the study design. I therefore suggest moving those three sentences to the earlier paragraph where you describe the selection of saplings, e.g. to line 135. I agree with Reviewer 1 that it is not clear if you rated individual saplings over 1-6 years (I think that's what you did but it isn't totally obvious) or if you used a different selection of saplings from each site in each year. This is easily fixed by changing "Saplings were rated" to "Each sapling was rated" on line 154. I also agree with the reviewer that your description of how replacement saplings were chosen is not as clear as it should be. On line 157, when you say "Dead saplings were replaced", were they replaced by the closest appropriate sapling, or by another sapling from somewhere within the same plot, or what? And on line 158, I suggest, "tolerant or resistant to H.frax". You could also say that only 1.5% of saplings in total were replaced over the study period (as I understand from your response to the reviewers of the original version), which indicates that this doesn't affect the majority of the data.

Done. More saplings where replaced in fact, although it still represent a minority. We computed that the mean replacement rate was of 18%. This is because *H. fraxineus* induce a high mortality rate at the sapling stage and cannot be avoided. This is now indicated in the text (148-149).

Line 295 (and elsewhere): Did you use a procedure to avoid for Type I errors in multiple significance tests?

Altogether, we tested only 5 models (comparison of 3 periods in spring for leaf necrosis frequency and of 2 in autumn for shoot mortality) to find the most suitable periods and did not account for Type I error. After, all variables were entered in the models. We simplified the presentation, as it was unnecessary to add a step in frequentist method. In the revision, we compare 5 Bayesian models with the BIC and hope it is much more clear now (L315-319).


Results

I appreciate that you have taken the approach of reporting the results concisely and factually, with little interpretation, which is good practice. However, there are many points where the text is unclear or ambiguous. Please consider the following suggestions for improvement.

Line 337: "very few leaf necrosis": To be clear, did you score the existence or absence of necrosis on a leaf as a categorical variable with two states? Or did you score the number / proportion of leaflets with necrosis within a leaf, or the area of necrosis? If you just scored presence / absence, a point for the Discussion is whether or not this is an accurate measure of disease development on an ash sapling.

We scored the leaf necrosis as a present / absence of necrosis on a leaf without quantifying the proportion / number of leaflet with necrosis. This is now indicated in L151-2. Then, we are not sure adding a long discussion thread on the way to measure foliar lesion is adequate as the manuscript is already complicated as you pointed out. We however added a sentence in the discussion L473-5. The number of leaflet affected may not be the real issues as lesions can be close/far from the stem or even directly on the rachis. It is a question of trade off. More detailed data is often desirable but usually come at the expenses of number of replicates. This is a very general debate...

Lines 339-340: This sentence doesn't make sense. Do you mean that the frequency of the presence of H.f. in leaves is similar to the visual estimate of the presence of leaf necrosis when necrosis is very frequent, but higher when necrosis frequency is low?

It is in fact the contrary. We deleted the sentence, as it appeared unclear. What was meant is that we make no mistake when we assess that H.f. induced abundant leaf necrosis and when we assess that H.f. induced little leaf necrosis, it is likely even more the case. The preceding sentence (L340-342) should indicate this clearly enough.

Lines 342-343: I don't understand how the percentage of leaves with necrosis can be similar on a plant with a dieback score of 0 or 1 ("complete absence of dead twigs" or "less than 10% dead twigs") and on one with a dieback score of 3 ("more than 50% of dead twigs"). Are you including the dead twigs in the assessment of % leaves with necrosis? If not (I can only assume you aren't), do you mean that the percent of leaves which are partly necrotic but still partly green is the same on trees with no shoot dieback or extensive shoot dieback? Or was the frequency of necrosis so high that almost all leaves had some necrosis, even on plants with little shoot dieback? At any rate, some major clarification or explanation of this point is required. Referring to my previous point, if the frequency of leaves with necrosis is high even on plants with a low dieback score, this indicates that the necrosis frequency has little power to discriminate healthier and sicker trees, and that a more quantitative score such as the frequency of necrotic leaflets or the area of necrosis would be more precise.

The term “dieback” just include shoot mortality. We added this in lines 136-7 to be clear. Indeed, defoliation by insects or leaf pathogens that are not chronic usually do not lead to dieback. This is what is occurring in our case. Then the rated shoots usually show no mortality in the autumn assessment in September. The shoot mortality occurs later, in December-January and mainly in February-April. So, no shoot mortality affect the frequency of leaf with necrosis. We thus indeed meant that the percent of leaves which are partly necrotic but still partly green is the same on trees with no shoot dieback or extensive shoot dieback. It has to be also pointed out that the bulk of leaf symptom observed is leaves which are partly necrotic but still partly green. Leaves can be occasionally completely necrotic, but this is not very common. We added that in the text (L345-347). Altogether, about 40-50% leaves have some partial necrosis as can be seen on figure 2. We hope that the paragraph is now easier to understand for readers not familiar with ash dieback. As we did no quantify leaf necrosis in more detail, we cannot prove that the “real” leaf necrosis severity on ash trees that show no or little shoot mortality is exactly the same as on those that show heavy shoot mortality. This was however our field impression. It remains highly speculative and not a major point in the manuscript. A point in discussion was however added L473-5.

Line 346: "the plot * dieback status interaction was not significant" - I appreciate that this may be an accurate report of a statistical test but it's one of several places where the biological relevance of the statistical result is unclear. Do you mean that the frequencies of different dieback scores were similar in different plots?

We rephrased the paragraph and hope it is now clearer

Lines 352-353: "The likelihood of shoot infection was significantly higher in [trees?] with severe dieback than in those with no dieback". As written, this seems trivial but do you mean that the likelihood of infection of new shoots was significantly higher in trees with more severe dieback?
First, we apologize, there was a mistake. What was meant is that the observed shoots had a mortality rate higher on saplings with dieback versus those not showing dieback. So it was not “shoot infection”. We rephrased the sentence and hope it is now clearer. Then it may be misleading to place it at the end after we report the overall dieback status of the sapling in 2015 and we moved the sentence.

Line 355: "Shoot mortality in spring was strongly related to leaf necrosis likelihood". Unclear: why 'likelihood of necrosis'? Why not just the amount of necrosis at the end of the previous summer? A similar comments applies to the sentences on lines 357-8 and 358-60.
The wording was changed for the 2 first sentences. However, I did not understand the problem with sentence L358-360.

Line 360: "leaf necrosis did not significantly depend on tree dieback status". This looks like the same point as on lines 342-3 but the statistics are different. Clarification needed.

L342-3 in the section “Ability of *H. fraxineus* to complete its cycle on ash saplings with different dieback severity” refers to a data set including only data from 2 plots (Gremecey and Seichamps) in 2014 autumn while the next paragraph (“Relationship between leaf necrosis in fall and subsequent shoot mortality”, L360) refers to the entire data set (55 plot*year combinations). We added a sentence in the first section to clarify that (L344).

Line 367: "affected by tree cover". In which direction? (I can see it in Fig. 3 but please say in the text.)

Done

Lines 394-5: "shoot mortality was strongly linked to leaf necrosis likelihood". (a) In which direction? (As for line 367.) (b) Why 'necrosis likelihood'? (As for line 355.)

Corrected

Line 395: "shoot mortality ... increased with increasing tree cover". This looks like the same point as you made on line 367. Clarification needed.

It is indeed the same phenomenon that was included in the plot level model as well as in the shoot level model because of its importance. It is here not that important to recall it and it was deleted.

Lines 415-6: "rachises producing apothecia ... not significantly related to ... leaf necrosis". In Table 4, there's a very large (negative) parameter relating apothecia to necrosis which isn't significant (P=0.3) but a much smaller (positive) parameter relating apothecia to rainfall, which *is* significant (P=0.006). Are you able to give a reason for this apparent disparity?
This is because in this analysis the variables were not standardized (mean=0, sd=1). So the large parameter value relating apothecia to leaf necrosis reflects that leaf necrosis frequency is in [0, 1] while the large parameter value relating apothecia to rainfall reflects that rainfall is in [40,120]. For consistency, we changed the analysis to show the result with standardized variables.

Discussion

Having written the Results section in a concise manner with little or no interpretation, it would greatly help readers to understand and evaluate the conclusions of your research if you referred to your figures and tables throughout the Discussion. The study is so complex with many explanatory variables which interact to affect three outcome variables differently (necrosis, mortality, apothecia), and have somewhat different effects at many locations, that it's very challenging to follow the Discussion without a clear guide as to which piece of data is relevant.

For example, your very first point in the Discussion, "healthy carriers exist": this requires the reader to piece together results from several tables and figures. There is some explanation in the next sentence but it would help readers greatly if each point in that sentence had references to the relevant data.

Done. We tried to refer to figures and tables throughout the discussion to help the readers.

A specific point is that your paper is of at least as much interest to plant pathologists as to ecologists but, unlike ecologist, few pathologists are familiar with bayesian statistics. Without more exposition of your conclusions, I frankly believe that most readers will find the Discussion very difficult to follow in detail. I'm familiar with bayesian methods but I myself found it pretty tough.
Looking back at my notes on the Discussion, most of them just say "Explain" - I think that gives you a flavour of what's needed!

This is a very difficult remark to take this into account as we have no ideas of where explanation is needed … We tried to refer to figure and table in the discussion section to help the reader to follow the discussion and hope that this help.

Specific points in the Discussion:

Line 438: Do you have any information which would allow you to extrapolate to other regions, beyond France?
We did not apply the model to other regions of Europe and thus have no precise information for elsewhere. I would expect that this might apply in many places on the southern limit of the disease, but in absence of detailed information… A sentence was added to say it might apply elsewhere (L446-7).

Line 446: Likewise, just in French conditions? From observation, I think this is true in the UK because I've very rarely seen apothecia even in early September. If there's hard evidence for this outside France, please give a reference.
The sentence was to explain was is observed in this study and thus refers to France. More information is available in other countries from spore traps. I am not aware of work in UK, but data exist in the Cech republic and Norway. Dvorak et al (2016) detect airborne inoculum in September-October and Timmermann et al (2011) detect some in September.

Line 492: You say that at low tree cover, crowns are exposed more frequently to adverse temperatures. Could there also be more wind or a dryer micro-environment, both of which are unfavourable to H.frax? (Extrapolating Chumanova's conclusions to the level of individual trees.)
we extended to other micro-climatic conditions known to be different under closed canopies (humidity)

Line 522-3 (sentence about microbiota): Interesting but rather speculative - omit?
done

Lines 543-5: I don't understand this sentence. I understand that infection can be promoted by rain but necrosis can be suppressed by high temperatures. But why does this explain why rainfall isn't correlated with symptoms, given that high summer rainfall usually involves lower temperatures in summer (but higher in winter)? Or doesn't the correlation between rainfall and lower temperature in summer apply so much in France?
In the study period, some years had adequate rainfall while summer temperature where very high. This is the general trend for many mid-latitude areas in Europe: the climate change with higher summer temperatures while summer rainfalls do not decrease. We now refer to Table 3 that show that this is what happened in the Champenoux area in 2019 (L560-3).

Lines 558-60: "H. fraxineus ability to infect ash leaves and reproduce on the rachises in the forest litter does not appears to limit the pathogen ability to establish throughout France. By contrast, the ability to induce shoot mortality is the limiting step." This is another point I don't understand because a key point of this paper is that reproduction of H.frax on fallen rachises is not closely connected with shoot mortality. Why then do you say that shoot mortality is a limiting step in the establishment of the pathogen? Especially because in Figure 7, you say that shoot mortality is a dead end for the pathogen.
Sorry, the wording was misleading. You are right. What we meant to say is that poor ability to induce shoot mortality explains the absence of disease report in southern France. We changed the sentence to clarify.

Tables 1 and 2: Please say that positive values indicate an association between the higher levels of the symptom and higher values of the environmental variable.
done

Figure 2C: On the y-axis, do you mean % shoots with *leaf* necrosis in spring 2015?
No, this is the % dead shoot in spring 2015. Corrected.

Reviewer #2:

L86: replace where with when
L210: "an engine" should probably be replaced by a better term, e.g. logging machinery?
Actually, the plot was partially destroyed in a house building operation (digging a pit for soil assessment). I do not really know what would be the good term.

L227: remove: "this is a common procedure frequently used in logistic regression."
Fig 2 could be improved by adding p.values
done


The quality of all figures in the latest submission seem to be very low (pixelated/unfocused/not sharp) they may need to be generated at higher resolution.

L370: please elaborate on "As no meso-climate effect was included in this model, this was expected." in line 254 you state that "The Env.l [i] and Env.s [i] random factors were taken as proxies for the environment, combining meso/micro climate and plot conditions (host density, Infection history)." both Env factors seem to be in the model (table 1).
done

373-373: unclear sentence: "with increasing shoot mortality with increasing defoliation level"
clarified

380-381: BIC values for "shoot mortality vs climate" model are the same for both time frames. Is that correct?
clarified

401-404: improve/clarify English. Use "Moreover, it was not linked to ... " rather than "Moreover, it was
significantly linked neither to ..."
done

432: "carrier trees without crown dieback symptoms" would be better than "healthy carrier"
done

436-438: "...it should not be able to induce significant dieback in large part of southern France." I feel the conclusion is a bit too strong given the variation of modelling outcomes in fig S2. More nuanced wording reflecting the effect of parameters presented in Fig S2 is required.
done

454: change capital "High" --> "high" also be more specific and clearly state that high levels of "leaf necrosis" rather that "levels" are not required for rachis colonisation.
done

455: "this would explain the observed result." feels out of place
Clarified

459:"within soil reach" better description of the sampling position required.
Clarified

461: "more humid close to soil and thus more favourable to H. fraxineus infection." (reference missing)
511-523: could this also be due to natural selection of less virulent local strains of the pathogen?
As we removed the reference to microbiome evolution as “too speculative”, we feel that adding a reference to selection of less virulent strain would also be so. Then, no proof of virulence evolution in H. fraxineus between long ago colonised area and new foci was found by Lygis et al (2017, 10.1111/ppa.12635).

534-538: split in 2 sentences please.
Done

556-557: reword "was by now never reported" to "This is the first report of..."
Done

figure 7 is a great addition but could be improved. Changing red and blue colour (perhaps as a scale) to designate only positive (shades of red) and negative (shades of blue) effects of the studied parameters rather than different parts of disease cycle would convene the gist of the study more clearly. The different parts of the disease cycle could use black and white arrows instead.
We tried and feel it does not improve the figure (some factor such as tree genotype are not +/- and is is difficylt to label differently the different part of the cycle outside the arrows). We thus slicked to the original figure.

Similarly the Fig 7 caption could better describe the actual dynamics of the model and try to avoid ambiguous terms such as "moderate", "presumably" which distract from the point of the figure. I propose the following caption format: "Higher summer rainfall and higher spring temperatures are associated with higher rates of leaf necrosis leading go higher shot necrosis especially in high tree cover. Lower summer temperature .... "
The caption was adapted as suggested
