# Supplementary material for: Ability of the ash dieback pathogen to reproduce and to induce damage on its host are controlled by different environmental parameters
Source: PLoS Pathog. 2023 Apr 20;19(4):e1010558. doi: 10.1371/journal.ppat.1010558 (PMC10153702; doi:10.1371/journal.ppat.1010558)
Supplement: Attachment — Submitted filename: Response_editeur23.docx [file ppat.1010558.s009.docx]

Dear editor,

Thank you for your suggestions, which we felt helped to improve the manuscript. We made all the suggested corrections. In particular, we realized that the word likelihood was present throughout the text and not only in figures and corrected that also. We changed the number of significant digits for pvalues to 2 throughout the text. Indeed, I also often struggle with students who put too much significant digits and agree that for pvalues, more than two is just both not wise and useful.

My best

Benoit Marçais
